# Benefits and Harms of Edible Vegetable Oils and Fats Fortified with Vitamins A and D as a Public Health Intervention in the General Population: A Systematic Review of Interventions

**DOI:** 10.3390/nu15245135

**Published:** 2023-12-18

**Authors:** Éva Szabó, Ildikó Csölle, Regina Felső, Daniela Kuellenberg de Gaudry, Patrick Nyamemba Nyakundi, Kazahyet Ibrahim, Maria-Inti Metzendorf, Tamás Ferenci, Szimonetta Lohner

**Affiliations:** 1 Cochrane Hungary, Clinical Centre of the University of Pécs, Medical School, University of Pécs, 7623 Pécs, Hungary; szabo.eva.dr@pte.hu (É.S.); csolle.ildiko@pte.hu (I.C.); felso.regina@pte.hu (R.F.); dkuellenberg@gmail.com (D.K.d.G.); 2 Department of Biochemistry and Medical Chemistry, Medical School, University of Pécs, 7624 Pécs, Hungary; 3 National Laboratory for Human Reproduction, University of Pécs, 7624 Pécs, Hungary; 4 Doctoral School of Health Sciences, Faculty of Health Sciences, University of Pécs, 7621 Pécs, Hungary; info.nyamemba@gmail.com; 5 Genomics and Bioinformatics Core Facility, Szentágothai Research Centre, University of Pécs, 7624 Pécs, Hungary; 6 Department of Public Health Medicine, Medical School, University of Pécs, 7624 Pécs, Hungary; kazahyet.i@gmail.com; 7 Institute of General Practice, Medical Faculty of the Heinrich-Heine-University Düsseldorf, 40225 Düsseldorf, Germany; maria-inti.metzendorf@med.uni-duesseldorf.de; 8 Physiological Controls Research Center, Obuda University, 1034 Budapest, Hungary; ferenci.tamas@nik.uni-obuda.hu; 9 Department of Statistics, Corvinus University of Budapest, 1093 Budapest, Hungary

**Keywords:** food fortification, edible vegetable oil, serum 25-OH vitamin D, serum retinol, vitamin A, vitamin D, systematic review

## Abstract

This systematic review aims to assess whether edible vegetable oils and fats fortified with vitamin A and/or D are effective and safe in improving vitamin intake and ameliorating deficiency states in the general population. In November 2022, we systematically searched MEDLINE, Cochrane CENTRAL, Scopus, Global Index Medicus, ClinicalTrials.gov, and WHO ICTRP (International Clinical Trials Registry Platform) for randomized controlled trials (RCT) and non-randomized studies of interventions (NRSI) investigating the fortification of edible vegetable oils and fats with either vitamin A or vitamin D or both as compared to the same vegetable oils and/or fats without vitamin A and D fortification or no interventions, in the general population, without age restriction. We assessed the methodological quality of included RCTs using Cochrane’s risk of bias tool 2.0 and of NRSIs using ROBINS-I tool. We performed random-effects meta-analysis and assessed certainty of evidence using GRADE. We included eight studies. Available evidence showed no significant effect of fortification with vitamin A on serum retinol levels (RCTs: MD 0.35 µmol/L, 95% CI −0.43 to 1.12; two trials; 514 participants; low-certainty evidence; CCTs: MD 0.31 µmol/L, 95% CI −0.18 to 0.80; two trials; 205 participants; very low-certainty evidence) and on subclinical vitamin A deficiency. Low-certainty evidence showed no effect of vitamin D fortification on serum 25-hydroxy vitamin D concentration (MD 6.59 nmol/L, 95% CI −6.89 to 20.07; one trial; 62 participants). In conclusion, vitamin A-fortified vegetable oils and fats may result in little to no difference in serum retinol levels in general populations. The dose of vitamin A used in the trials may be safe but may not be sufficient to reduce subclinical vitamin A deficiency. Further, the evidence suggests that vitamin D fortification results in little to no difference in serum 25-hydroxy vitamin D concentration. Several aspects of providing fortified oils and fats to the general population as a public health intervention should be further investigated, including optimal fortification dose, effects on vitamin D deficiency and its clinical symptoms and potential adverse effects.

## 1. Introduction

Food fortification, where essential micronutrients are added to widely consumed staple foods and condiments during production, either compulsorily or voluntarily, is a strategy that has been used safely and effectively for more than a century to prevent micronutrient deficiencies and related health problems in high-income countries [1]. Compared to voluntary food fortification, which is primarily used for marketing purposes, public health fortification campaigns aim to address vitamin and mineral deficiencies at the population level without creating economic inequalities (homogeneous affordability) [1].

Vitamin A is a group of fat-soluble molecules with a similar structure, including retinol, retinal, retinoic acid, and several provitamin A carotenoids (most notably beta-carotene) [2]. Vitamin A has diverse functions: it is essential for vision, for embryo development and growth and for maintaining the immune system [3]. Therefore, vitamin A deficiency (VAD) can impair the function of neutrophils, macrophages, NK cells, and diminish the Th2 cytokine-production and Th1-mediated immunity [2]. VAD is a major nutritional problem in many parts of the world, especially in low-income countries, leading to a number of health problems, including xerophthalmia, increased susceptibility to infections and anemia. VAD is the leading cause of preventable blindness [4], but children with VAD are at increased risk of morbidity and mortality as well [5]. The risk factors for the development of VAD are multifactorial, including demographic (mainly men and preschool children), geographical (mainly in Africa and Southeast Asia), childhood (breastfeeding, infections), household (lower socioeconomic status, poor hygiene), and dietary (lower quality and diversity of diet) factors [6]. The World Health Organization (WHO) estimates that VAD affected an estimated 190 million pre-school children and 19.1 million pregnant women worldwide between 1995 and 2005, mainly in Africa and Southeast Asia [7].

Vitamin D_3_ or cholecalciferol, another fat-soluble vitamin, can be taken up with food, but the main source is the endogenous synthesis in the skin. The active form is obtained after two hydroxylation steps and is called calcitriol (1,25-dihydroxy vitamin D_3_) [8]. Vitamin D plays a central role in calcium homeostasis, and therefore in bone mineralization [9], but it also has immunomodulatory effects in both innate and adaptive immunity, and through the immune cells, in both acute and chronic inflammation as well as in the pathomechanism of several autoimmune processes [8]. Dietary sources of vitamin D, including eggs, dairy products, meat, and fish, are limited, so commercially fortified products make a sizeable contribution to daily dietary intake [10,11]. Vitamin D deficiency is a global health problem affecting all age groups in almost every country in the world. The global burden of vitamin D deficiency is hard to quantified, as different definitions of deficiency exist, which are all based on serum 25-hydroxyvitamin D levels (25(OH)D) [12]. The determinants of lower vitamin D status may vary depending on the location (lower exposure to sunlight, lower consumption of vitamin D-containing foods, urbanization, air pollution, higher body mass index (BMI)) [13]. Vitamin D deficiency can primarily cause symptoms in the bones, namely reduced mineralization, leading to nutritional rickets in children and osteoporosis in adults [14], as well as chronic inflammation, autoimmunity, and the increased frequency of infections [8,15].

There are three main strategies which might be effective in the prevention of vitamin deficiencies: increasing diversity, supplementation, and food fortification. Improvements in food diversity are difficult to achieve when limited amounts of food items with high vitamin content are available. Supplements are usually used by a small proportion of the population; therefore, food fortification is the strategy preferred by the WHO in terms of coverage [16].

Edible vegetable oils and fats are one of the most important staple foods worldwide because of their energy density, but they are also natural sources of fat-soluble vitamins (A, D, E, and K) and act as a solvent to enhance the absorption of fat-soluble vitamins. Edible vegetable oils and fats are consumed widely, regardless of wealth. The production of vegetable oils more than doubled between 2000 and 2019 [17]. In most countries they are processed centrally by medium and large-scale producers, which facilitates the implementation and monitoring of a potential fortification process [10].

Existing systematic reviews and meta-analyses mainly focus on the health outcomes of vitamin A or vitamin D fortification of all types of staple foods in the general population [18,19] or in children only [20,21,22] and are mainly based on results from clinical trials. A lower number of evidence summaries focus on the fortification of specific vehicles (e.g., bread [23] and yoghurt [24]), but no systematic review has been published on the effects of vitamin A or vitamin D fortification of edible oils and fats in the general population. 

The current systematic review aims to synthetize up-to-date data from both interventional and observational trials and provide a systematic assessment of the benefits and harms of edible oils and fat fortified with vitamin A or vitamin D, either alone or in combination to inform policymaking and assist countries in the design and implementation of appropriate food-fortification programs.

## 2. Materials and Methods

The methodology and the results are reported according to the Preferred Reporting Items for Systematic reviews and Meta-Analyses (PRISMA) reporting guidelines. This study is registered with PROSPERO, CRD42022351689.

### 2.1. Search Strategy

For this systematic review and meta-analysis, we searched the following electronic databases and trial registers from the inception of each database up to 14 November 2022 without restrictions on the language of publication: Ovid MEDLINE, Cochrane Central Register of Controlled Trials (CENTRAL), Global Index Medicus (comprising African Index Medicus (AIM), Index Medicus for the Eastern Mediterranean Region (IMEMR), Index Medicus for the South East Asia Region (IMSEAR), Latin America and the Caribbean Literature on Health Science (LILACS), and Western Pacific Region Index Medicus (WPRO)), Scopus and trial registers (https://clinicaltrials.gov/, WHO ICTRP (International Clinical Trials Registry Platform; apps.who.int/trialsearch)). Details for all search strategies are available in Supplementary File S1.

Using the reference lists of included studies, related systematic reviews, meta-analyses, and health technology assessment reports, we attempted to identify other potentially eligible trials or additional publications. We searched for grey literature, which we defined as searching the Global Index Medicus, as well as trial registers.

### 2.2. Eligibility Criteria

We included randomized controlled trials (RCTs), controlled clinical trials (CCTs), cohort studies, controlled before-after studies, and interrupted time series. For cluster randomized trials, non-randomized cluster trials, and controlled before–after studies, we only included studies with at least two intervention sites and two control sites. We included the general population (including pregnant women), comprising individuals of any age and from any country. Studies of interventions targeted toward participants with a critical illness or severe comorbidities were excluded. 

The eligible interventions were edible oils and/or fats (of vegetable origin) for household use fortified with either vitamin A or vitamin D or a combination of vitamin A and D compared to no intervention or the same unfortified oil and/or fat. No restriction was made regarding the type of vegetable oil (extracted from seeds or from other parts of fruits). We excluded studies comparing vitamin A and/or vitamin D oil or fat fortification with other forms of vitamin A and/or vitamin D interventions (i.e., supplementation or dietary diversification) or fortification of other food vehicles (e.g., sugar, flour, milk, and dairy products). 

### 2.3. Selection Process

Pairs of review authors (ES, DK, IC, RF, PNN, KI, SL) independently screened the abstract, title, or both, of every record retrieved by the literature searches using COVIDence^TM^ software, https://www.covidence.org/. We obtained the full texts of all potentially relevant records and screened these for eligibility. Any disagreements were resolved through consensus or by recourse to a third review author (SL). Potentially relevant articles written in a language other than English were translated to English prior to full text assessment. Multiple reports of the same study were merged, as each study rather than each report was the unit of interest in this review. All articles excluded after full-text assessment and the reasons for their exclusion are described in the table on characteristics of excluded studies (Supplementary File S2). The trial selection process is presented in a PRISMA flow diagram.

### 2.4. Data Collection

From the full-text publications, we extracted data on study methods, participants, interventions, controls, outcomes, confounders, and funding sources. Data were extracted by one reviewer (IC or RF) and verified for completeness, accuracy, and consistency by a second reviewer (IC or RF).

We included abstracts and conference proceedings but did not use them to extract data, as they did not meet CONSORT requirements. We also extracted data available in the study registers as study results.

The main outcomes, defined by the WHO guideline development group (GDG), were markers of vitamin A and/or D deficiency (measured as serum retinol, serum 25(OH)D, subclinical/clinical VAD, vitamin D deficiency, osteomalacia, nutritional rickets), all-cause morbidity and mortality, and any adverse effects. Additional outcomes were vitamin A status, dietary vitamin A/D intake, iron status, anemia, maternal and infant outcomes, growth, weight change, and any longer-term outcomes. We included outcomes as measured at any given timepoints. 

We extracted data on study information, participants, type of intervention, type of outcomes (both primary and secondary outcomes specified and collected, timepoints reported), adjusted and unadjusted outcome measures, confounders, methods used to control confounders, funding, and any notable conflicts of interest of the study authors. Studies reporting outcomes at multiple timepoints, we extracted data for each timepoint. Data extraction was performed by one reviewer and was checked for completeness, accuracy, and consistency by a second independent reviewer. We attempted to obtain missing data from the study investigators.

### 2.5. Risk of Bias Assessment

Two review authors (ÉS and DK) independently assessed the risk of bias of each included trial. Any disagreements were resolved by consensus. Risk of bias in RCTs was assessed using version 2.0 of the Cochrane “Risk of bias” tool (RoB2), while in NRSIs (including quasi-randomized studies, cohort studies, controlled before-and-after studies, and interrupted time series) were assessed using the “Risk of Bias in Non-randomized Studies of Interventions” (ROBINS-I). To illustrate the risk of bias judgements for RCTs and NRSIs, we used the robvis tool to create traffic light plots [25].

### 2.6. Effect Measures

For dichotomous data, we present results as risk ratios (RRs) or odds ratios (ORs) with 95% confidence intervals (CIs). For continuous data, we use mean differences (MDs) with 95% CIs for studies measuring outcomes in the same way and standardized mean differences (SMDs) with 95% CIs for studies measuring outcomes in a variety of ways.

### 2.7. Synthesis Methods

We used RevMan 5 (version 5.4.1) for statistical analyses. As we expected differences between studies in both the population and the intervention, we decided to combine the data using a random effects model, when it was clinically meaningful to do so, to provide an average treatment effect across studies. We used Mantel–Haenszel weighting for dichotomous outcomes and inverse variance for continuous outcomes. In case both individually randomized and cluster-randomized trials were included in a meta-analysis, we planned to use the inverse variance method.

Methodological heterogeneity was assessed by examining risk of bias, while clinical heterogeneity was assessed by examining similarities and differences between studies regarding types of participants, interventions, and outcomes. We considered the size and direction of effect and used a standard χ^2^ test with a significance level of α = 0.1 and I^2^ statistic, quantifying inconsistency across trials, to assess the impact of heterogeneity on the meta-analysis. We explored heterogeneity by conducting pre-specified subgroup analyses. 

We planned to perform subgroup analyses for the following characteristics for both vitamin A and D: age groups, psychological condition, vitamin A/D intake, public health significance of vitamin A/D deficiency in the trial’s country, vehicle of intervention, consumption patterns, duration of intervention, amount of added vitamin A/D through fortification, type of vitamin compound, type of fortification intervention, method of cooking, and delivery platform. We planned additional subgroup analyses for vitamin D only for: skin pigmentation, latitude, exposure to environmental pollutants, BMI, exposure to additional vitamin D though other programs, and as a method to stabilize vitamin D.

We planned to conduct sensitivity analyses to examine the potential effects of clustering on the CIs of summary estimates. 

### 2.8. Reporting Bias Assessment

We planned to use funnel plots to assess reporting bias (such as publication bias) and to investigate the relationship between effect size and standard error when 10 or more studies were included in a meta-analysis. The degree of funnel plot asymmetry was planned to be quantified using Egger’s test.

### 2.9. Certainty Assessment

We followed the GRADE approach to rate the certainty of evidence [26].

## 3. Results

### 3.1. Description of Included Studies

**Figure 1:**
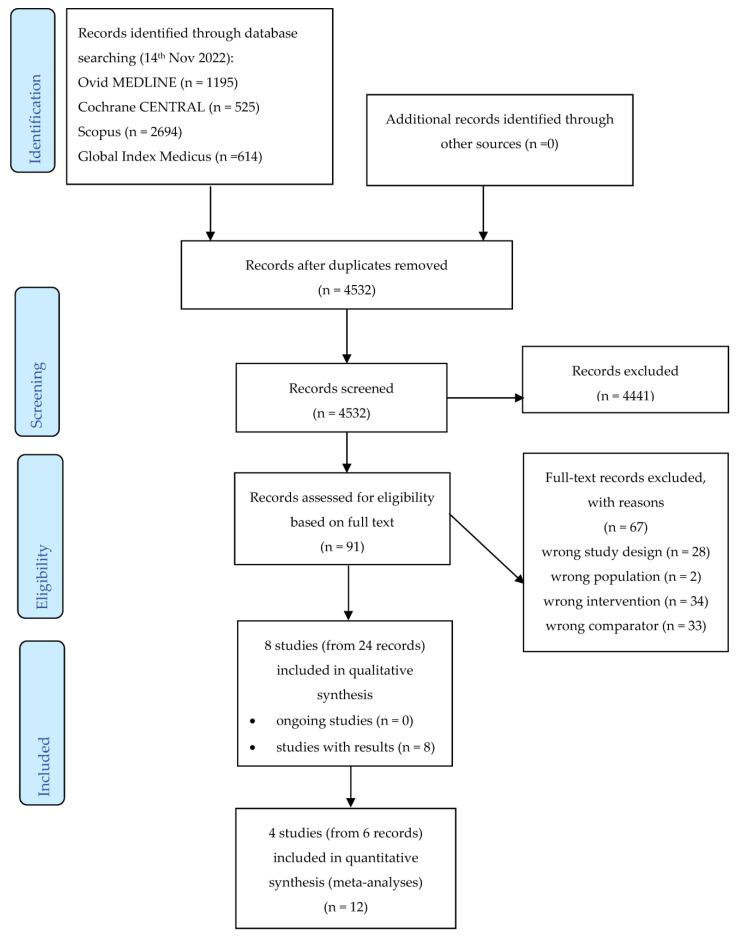
Study selection. CENTRAL: Central Register of Controlled Trials; ICTRP: International Clinical Trials Registry Platform.

We retrieved 5678 unique records through database searching (Figure 1). After removing duplicates, 4532 records were screened based on their titles and abstracts. Most of the references (*n* = 4441) clearly did not meet the inclusion criteria based on title and abstract review and were excluded. We evaluated 91 full texts or records to determine their eligibility for inclusion in the review. Of these, 28 studies were excluded because they were not RCTs or NRSIs, 2 studies were excluded because the participants were people with a specific disease, 34 studies were excluded because the intervention/exposure was not an oil or fat fortified with either vitamin A or vitamin D or their combination, and 33 studies were excluded because there was no eligible comparator (Supplementary File S2). Eight studies (reported in 24 records) met our inclusion criteria for qualitative, and four studies (reported in 6 records) met the requirements for quantitative synthesis. One of the included studies (with 13 associated records) was a large birth cohort study based on the cancellation of mandatory fortification of margarine in Denmark in 1985, which we will refer to as the “Danish study” (Supplementary File S3).

A total of five studies were included for the comparison of vitamin A fortification versus no fortification with vitamin A (Table 1), including two RCTs [27,28,29,30], two CCTs [31,32] and one birth cohort study [33]. Participants in all RCTs and CCTs were allocated to groups at the individual level.

A total of three studies were included for the comparison of vitamin D fortification versus no fortification with vitamin D (Table 2), including one RCT [34,35,36] and two birth cohort studies (Stougaard 2018 [37] and the Danish Study [38,39,40,41,42,43,44,45,46,47,48,49]). No studies investigated the combined effects of vitamin A and D fortification. In four studies, oil was fortified with vitamin A [27,28,31,32], and in one study [33], margarine was fortified with vitamin A, while one study used oil [34] and two studies used margarine [37,41] for vitamin D fortification.

Three studies were conducted in a high-income country [33,37], one was conducted in an upper-middle-income country [32], while four studies were conducted in lower-middle-income countries including Indonesia [31], the Philippines [27], Morocco [28], and Iran [35], while no study was conducted in a low income country.

Participant age ranged from 4 to 40 years, while in the birth cohort studies, fetuses or pregnant women were either exposed or not exposed. Sample sizes ranged from 31 [31] to 331,623 [44]. Among studies investigating longer-term effects of fortified edible oil consumption, intervention duration lasted between 8 weeks [31] and 6 months [27,28], while in birth cohort studies, there were no detailed information about intervention duration.

### 3.2. Risk of Bias in Included Studies

Overall, two randomized trials (66%) [27,28,29] were rated as having a moderate risk of bias, while one study was evaluated as having a low risk of bias [34,35]. In the included non-randomized trials, two studies [32,33] were rated as having a high risk of bias due to the selection of participants, while 60% of the articles had a moderate risk of bias (Supplementary File S4).

### 3.3. Primary Outcomes for Studies on Vitamin A Fortification versus No Fortification with Vitamin A

Two randomized [27,28] and two non-randomized studies [31,32] with intervention durations of 6 months and 2–5 months, respectively, measured serum retinol. Available evidence based on RCTs showed no effect of fortification with vitamin A on serum retinol levels (MD 0.35 µmol/L, 95% CI −0.43 to 1.12; two trials; 514 participants; low-certainty evidence, Table 3), also supported by evidence derived from non-randomized studies (MD 0.31 µmol/L, 95% CI −0.8 to 0.80; two trials; 205 participants; very low-certainty evidence; Supplementary File S5, p. 1).

Similarly, no effect on subclinical vitamin A deficiency, measured as serum retinol ≤ 0.70 µmol/L in one RCT (0/268 vs. 0/144, RR not estimable, one trial, low-certainty evidence, Table 3), supported by evidence derived from the CCT (RR 0.21, 95% CI 0.01 to 4.10; one trial; 31 participants; very low-certainty evidence; Supplementary File S5, p. 6) and no effect on all-cause morbidity (low certainty and very low-certainty evidence, respectively) of fortification with vitamin A was found. 

All-cause morbidity was measured in two RCTs [27,28] and one CCT [31]; however, only one RCT [27] and one CCT [31] reported results. Neither the RCTs nor the CCTs described differences between groups in all-cause morbidity (low certainty and very low-certainty evidence, respectively, Table 3). No studies reported data on clinical vitamin A deficiency, adverse effects, or all-cause mortality.

**Table 2:** Key characteristics of included studies with vitamin D fortification as the intervention.

Study ID	References	Country	Study Design	Sample Size (*n*)	Age at Exposure	Age at Outcome Measurement	Fortified Product	Micronutrient(s) Added to the Fortified Products	Duration of Intervention	Outcomes
Ghasemifard 2020	[33,34,35]	Iran	randomized controlled trial	99	18–30 years	18–30 years	Canola oil	Vitamin D	12 weeks	Se levels of 25(OH)D, CTX, B-ALP, PTH, TC, LDL, HDL, TG; dietary intake of energy, protein, vitamin D, vitamin K, vitamin C, calcium, phosphorus, magnesium, zinc,
Stougaard 2018	[35]	Denmark	birth cohort study	73,237	during pregnancy	NA	Margarine	Vitamin D	NA	Gestational hypertension, preeclampsia (including mild and unspecified preeclampsia and toxemia), eclampsia (including severe preeclampsia and eclampsia)
Danish study	[36]	Denmark	birth cohort study	217,249	during fetal life	NA	Margarine	Vitamin D	NA	Incidence of celiac disease
	[37]	Denmark	birth cohort study	217,249	during fetal life	NA	Margarine	Vitamin D	NA	Incidence of IBD (including Crohn’s disease, ulcerative colitis, unidentified IBD)
	[38,39,40]	Denmark	birth cohort study	28,871	during fetal life	20.6–27.5 years	Margarine	Vitamin D	NA	Incidence of gestational diabetes mellitus
	[41]	Denmark	birth cohort study	222,247	during fetal life	0–9 years	Margarine	Vitamin D	NA	Incidence of childhood asthma (diagnoses from birth to the age of 9 years)
	[42,43]	Denmark	birth cohort study	331,623	during fetal life and during first postnatal year	0–15 years	Margarine	Vitamin D	NA	Incidence of type 1 diabetes mellitus (before age of 15 years)
	[44]	Denmark	birth cohort study	327,254	during fetal life	10–18 years	Margarine	Vitamin D	NA	Number of fracture events
	[45]	Denmark	birth cohort study	30,004	during fetal life	0 year	Margarine	Vitamin D	NA	Birth weight, prevalence of low and high birth weight
	[46]	Denmark	birth cohort study	30,004	during fetal life	7 years	Margarine	Vitamin D	NA	Birth weight, BMI, BMI Z-score, prevalence of overweight and obesity (at 7 years of age)
	[47]	Denmark	birth cohort study	35,435	during fetal life	14.5–27.5 years	Margarine	Vitamin D	NA	Gestational hypertension, preeclampsia (including mild and unspecified preeclampsia and toxemia), eclampsia (including severe preeclampsia and eclampsia)

Se: serum; 25(OH)D: 25-hydroxy vitamin D; CTX: collagen type 1 cross-linked C-telopeptide; B-ALP: bone-specific alkaline phosphatase; PTH: parathyroid hormone; TC: total cholesterol; LDL: low-density lipoprotein; HDL: high-density lipoprotein; TG: triglyceride; NA: not applicable; IBD: inflammatory bowel disease; BMI: body mass index.

**Table 3:** Vitamin A-fortified oils or fats compared to same oils or fats without vitamin A in the general population as a public health intervention.

Certainty Assessment							No of Patients		Effect		Certainty	Importance
No of Studies	Study Design	Risk of Bias	Inconsistency	Indirectness	Imprecision	Other Considerations	Vitamin A-Fortified Oils or Fats	Same Oils or Fats without Vitamin A	Relative (95% CI)	Absolute (95% CI)		
Serum retinol (µmol/L)—Randomized Studies (follow-up: 6 months)												
2 [27,28]	randomized trials	serious ^a^	serious ^b^	not serious	not serious ^c^	none	307	207	-	MD 0.35 µmol/L higher(0.43 lower to 1.12 higher)	⊕⊕◯◯ Low	CRITICAL
Serum retinol (µmol/L)— non-randomized studies (follow-up: between 2 and 5 months)												
2 [31,32]	randomized trials	serious ^d^	serious ^b^	not serious	serious ^e^	none	102	103	-	MD 0.31 µmol/L higher(0.18 lower to 0.8 higher)	⊕◯◯◯ Very low	CRITICAL
Subclinical vitamin A deficiency (serum retinol ≤ 0.70 µmol/L)—randomized studies (follow-up: 6 months)												
1 [27]	randomized trials	serious ^f^	not serious	not serious	serious ^g^	none	0/268 (0.0%)	0/144 (0.0%)	not estimable		⊕⊕◯◯ Low	CRITICAL
Subclinical vitamin A deficiency (serum retinol ≤ 0.70 µmol/L)—non-randomized studies (follow-up: 2 months)												
1 [31]	randomized trials	serious ^f^	not serious	not serious	very serious ^h^	none	0/15 (0.0%)	2/16 (12.5%)	RR 0.21 (0.01 to 4.10)	99 fewer per 1 000 (from 124 fewer to 387 more)	⊕◯◯◯ Very low	CRITICAL
Clinical vitamin A deficiency (xerophthalmia, night blindness)—not measured												
-	-	-	-	-	-	-					-	CRITICAL
All-cause morbidity—randomized studies (follow-up: 6 months)												
2 [27,28]	randomized trials	very serious ^i^	not serious	not serious	not serious	none	Out of two RCTs measuring morbidity, one (with 268 participants in the intervention and 144 in the control group) reported results as frequency and duration of illness. This study reported no significant differences between study groups.				⊕⊕◯◯ Low	CRITICAL
All-cause morbidity—non-randomized studies (follow-up: 2 months)												
1 [31]	randomized trials	Serious ^f^	not serious ^j^	not serious	very serious ^h^	none	One CCT reported morbidity scores (defined as frequency of illness multiplied by duration of illness) and described no significant differences between study groups.				⊕◯◯◯ Very low	CRITICAL
All-cause mortality—not measured												
-	-	-	-	-	-	-					-	CRITICAL
Adverse effects (hypervitaminosis, liver toxicity)—not measured												
-	-	-	-	-	-	-					-	CRITICAL

CI: confidence interval; MD: mean difference; RR: risk ratio. Explanations: ^a^. Downgraded by one level for RoB since both included studies were rated with some concerns for RoB. ^b^. Downgraded for inconsistency as point estimates varied widely, 95% CI did not overlap between studies, the direction of effect was not consistent, and the magnitude of heterogeneity was high (I2 was 98%, *p*-value for heterogeneity was <0.0001). Sub-group analyses did not fully explain heterogeneity. ^c^. Not downgraded for imprecision. Although only two studies were included, the magnitude of the median sample size was intermediate (*n* = 257), and the total sample size was larger than 400 (*n* = 514). ^d^. Downgraded by one level for RoB since one of the two included studies was rated with a high RoB, and none of the included studies were rated with a low RoB. ^e^. Downgraded by one level for imprecision since the number of included studies was small (*n* = 2), the magnitude of the median sample size was intermediate (*n* = 103), and the total sample size was smaller than 400 (*n* = 205). ^f^. Downgraded by one level for RoB since the included study was rated with some concerns for RoB. ^g^. Downgraded by one level for imprecision. There was only one study included, but the total sample size was larger than 400 (*n* = 412). ^h^. Downgraded by two levels for imprecision since results are derived from one study, where total sample size was very low (*n* < 100). ^i^. Downgraded by two levels for RoB, as results were not reported for one out of two studies, and additionally, because none of the included studies was rated with a low RoB. ^j^. This is a single study so inconsistency cannot be judged.

### 3.4. Primary Outcomes for Studies on Vitamin D Fortification versus No Fortification with Vitamin D

Serum 25(OH)D concentration was measured in a single RCT involving 62 participants [35], and based on this trial, no difference between the groups was found (MD 6.59 µmol/L, 95% CI −6.89 to 20.07; low-certainty evidence; Supplementary File S6, p. 1). No studies reported data on vitamin D deficiency, osteomalacia, nutritional rickets, any adverse effects, morbidity, and mortality.

No studies reported data on vitamin D deficiency, osteomalacia in elderly, nutritional rickets, adverse effects, all-cause morbidity, or mortality.

### 3.5. Secondary Outcomes for Studies on Vitamin A Fortification versus No Fortification with Vitamin A

Low-certainty evidence from one RCT [27] showed no difference in vitamin A intake of participants of the vitamin A-fortified group compared to non-fortified groups (MD 15.7 µg RE/day, 95% CI −105.82 to 74.42; one trial; 412 participants), while very low-certainty evidence derived from one CCT [31] showed significant effects of fortification (MD 240.6 µg RE/day, 95% CI −175.1 to 306.1; one trial; 31 participants; Supplementary File S5, p. 7). 

The consumption of vitamin A-fortified oil resulted in better vitamin A status based on one trial [28] measured as higher breast milk retinol (MD 0.79 µmol/L, 95% CI 0.72 to 0.86; one trial; 63 participants; very low-certainty evidence) or lower risk of low breast milk retinol concentration (<1.05 µmol/L; RR 0.04 µmol/L, 95% CI 0.01 to 0.14; one trial; 101 participants; very low-certainty evidence; Supplementary File S5, p. 7).

### 3.6. Secondary Outcomes for Studies on Vitamin D Fortification versus No Fortification with Vitamin D

Vitamin D fortification resulted in no effect on vitamin D intake based on one RCT (MD 22.35 mcg/day, 95% CI −52.88 to 8.18; one trial; 62 participants; low-certainty evidence; Supplementary File S6, p. 1) [35]. 

In terms of maternal and infant outcomes, there was no difference in the incidence of gestational diabetes mellitus between the vitamin D-fortified and non-fortified groups based on one birth cohort study [40,42] (RR 0.87, 95% CI 0.75 to 1.01; one trial; 28,871 participants; very low-certainty evidence). A birth cohort study [37] showed that preeclampsia also did not differ between the exposed and non-exposed groups (RR 1.04, 95% CI 0.96 to 1.12; one trial; 73,237 participants; very low-certainty evidence). The incidence of children born with low birth weight (<2500 g) was found to be not different among offspring of mothers consuming vitamin A-fortified margarine during pregnancy and newborns whose mothers did not consume vitamin A-fortified margarine (RR 1.16, 95% CI 0.99 to 1.35; 1 trial; 10552 participants; very low-certainty evidence) [47]. 

The childhood effects of fetal exposure to vitamin D-fortified margarine consumed by pregnant women were investigated in five publications of the Danish Study. Based on this birth cohort study [48], lower BMI (MD −0.1 kg/m^2^, 95% CI −0.17 to −0.03; one trial; 10832 participants; low-certainty evidence), lower risk of overweight (RR 0.92, 95% CI 0.86 to 0.98; one trial; 10,832 participants; low-certainty evidence), and lower risk of obesity (RR 0.85, 95% CI 0.77 to 0.95; one trial; 10,832 participants; low-certainty evidence) was observed in children of mothers exposed to vitamin D-fortified margarine during pregnancy as compared to women consuming margarine without additional vitamin D, at the age of seven.

This birth cohort study [46] also investigated childhood fracture events and showed lower risk of fracture events in the vitamin D-fortified group (RR 0.84, 95% CI 0.82 to 0.85; one trial; 217,983 participants; low-certainty evidence). In the same birth cohort [44,45], the maternal consumption of vitamin D-fortified margarine during pregnancy was associated with a lower risk of developing type-1 diabetes mellitus in their children up to the age of 15 years (RR 0.78, 95% CI 0.67 to 0.91; one trial; 261,956 participants; low-certainty evidence). The risk of childhood asthma did not differ between the two groups (RR 0.96, 95% CI 0.90 to 1.03; one trial; 222,247 participants; low-certainty evidence) [43]. 

Consumption of vitamin D-fortified oil had no effect on the serum parathyroid hormone (MD 0.10 pmol/L, 95% CI −0.99 to 1.19; one trial; 36 participants; low-certainty evidence) or serum alkaline phosphatase levels (MD 5.76 IU/L, 95% CI −0.12 to 11.64; one trial; 36 participants; low-certainty evidence) in healthy adults [34]. 

## 4. Discussion

To our knowledge, this is the first systematic review summarizing evidence on the consumption of vitamin A and/or D-fortified edible oil or fat compared with the unfortified version of the same oil or fat in a general population. The evidence suggests that vitamin A fortification may result in little to no difference in serum retinol levels in general populations. The dose of vitamin A used in trials may be safe but may not be sufficient to reduce subclinical vitamin A deficiency. Similarly, the consumption of vitamin D-fortified oils and/or fats may result in little to no difference in serum 25(OH)D concentrations. Available evidence suggests that vitamin A-fortified oils/fats might increase dietary vitamin A intake, and therefore vitamin A status; however, there is no current evidence that vitamin D intake is increased by consuming vitamin D-fortified oils/fats. There is no current evidence that gestational vitamin D fortification can influence maternal and neonatal outcomes, but it might be beneficial in growth and weight gain in childhood and might also have some longer-term health effects. 

A significant strength of this study is that we used a broad search strategy in both electronic databases and trial registries without applying date or language restrictions. It is unlikely that published trials have been missed; however, unpublished or ongoing trials not registered in clinical trial registries could be missing. Secondly, we aimed to reduce bias wherever possible by having at least two review authors work independently on trial selection, data extraction, and “Risk of bias” assessments. We examined the general population without age restrictions, so that our results can be used widely, not just in certain age groups. Finally, we examined the effects of the fortification of edible oils/fats only, thus reducing the effects of potentially different vitamin absorption from different food types.

However, a major limitation of this systematic review is that several prespecified outcomes were investigated in a small number of trials or that no data were available at all. Due to the low number of studies, we were also not able to explore the potential for publication bias using a funnel plot.

Most existing reviews have addressed the effects of different food vehicles like dairy products, flour, grains, and oils, but most include results mainly from controlled trials and do not considered specific population subgroup analysis. There are only a few meta-analyses discussing the effect of vitamin A [18] or vitamin D [19,23] in the general population; most of them included either only children [20,21,22] or only adults [24,50,51,52]. Almost all meta-analyses found a consistent improvement in vitamin 25(OH)D levels with vitamin D fortification [19,21,22,23,24,51,52], while the effect of vitamin A fortification is less clear [18,20]. By contrast, in this meta-analysis, no significant effect was found on either serum 25(OH)D values or serum retinol, although we were able to only include one RCT for vitamin D fortification and two RCTs as well as two CCTs for vitamin A fortification. Most meta-analyses focused only on serum retinol or serum 25(OH)D levels, while some also investigated the effect of fortified food on clinical/subclinical VAD [18] or other cognitive functions [21].

Although oils and fats are widely consumed staple foods worldwide, providing an ideal solvent for fat-soluble vitamins, there are only a few clinical trials that have investigated the effects of vitamin A/D fortification on edible vegetable oils or fats. Our results suggest that the doses used in the trials so far are safe, but further clinical studies are needed to establish effective doses for the prevention of vitamin A and/or D deficiency. Future research should also clarify the stability of added vitamin A/D in different oils and fats under various conditions and types of usage, as very diverse factors can influence this.

When considering the advantages and disadvantages of the implementation of the fortification of edible oils and fats, it is essential that the worldwide consumption of edible oils and fats; the effectiveness and safety of currently existing fortifying policies; challenges during implementation; and aspects of cost-effectiveness, acceptability, and the potential impact on non-communicable diseases (NCDs) are taken into account.

Currently, 35 countries have mandatory policies and eight countries voluntary policies regarding the fortification of edible oils, mainly in Asian and African countries [11]; however, only about 40% of the population consume fortified vegetable oil and 34% consume adequately fortified oil based on a recent meta-analysis [53]. On the other hand, core micronutrient deficiencies, including of iron, zinc, and vitamin A, are still high worldwide, affecting nearly half of pre-school children and non-pregnant women of reproductive age [54]. 

Fat and oil fortification guidelines should be developed with consideration of the broader nutritional context. Based on guidance formulated by the WHO, total fat should not exceed 30% of total energy intake, the intake of saturated fats should be less than 10%, and that of trans-fats less than 1% of total energy intake [55]. Edible oils have different saturated fat contents and fatty acid profiles [56]. Currently, palm oil is the most commonly produced oil worldwide, followed by some healthier alternatives, including soybean, rapeseed, and sunflower oils [57].

Based on the regulations of Codex General Principles for the Addition of Essential Nutrients to Foods “fortification should be the responsibility of national authorities since the kinds and amounts of essential nutrients to be added and foods to be fortified will depend upon the particular nutritional problems to be corrected, the characteristics of the target populations, and the food consumption patterns of the area” [58]. Although oil consumption should not be promoted in any way, it should be taken into account that in some countries, adequate micronutrient intake through healthy diets is not feasible for large groups of people, so vitamin intake through processed food, which is otherwise consumed regularly, may be a possible solution to prevent vitamin deficiencies.

Although vitamin A and D deficiency is a global health problem and the fortification of oils and fats with vitamin A and D might be a safe strategy that countries could consider making part of their strategy to tackle deficiencies, the results based on the included studies suggest that vitamin A- and D-fortified oils have little or no effect on health; however, more studies are needed as the sample size is presently very low, meaning that the presence of small effects that might be still relevant on the population level, cannot be excluded with a high degree of certainty.

In conclusion, vitamin A and D deficiencies are global health problems, and the fortification of oils and fats with vitamin A and D might be a safe strategy which countries could consider making part of their policies to tackle deficiencies, after assessing local circumstances. In order to be able to formulate recommendations based on higher-certainty evidence, further studies investigating the effectiveness and safety of vitamin A and D fortification are needed. 

## Figures and Tables

**Table 1:** Key characteristics of included studies with vitamin A fortification as the intervention.

	References	Country	Study Design	Sample Size (*n*)	Age at Exposure	Age at Outcome Measurement	Fortified Product	Micronutrient(s) Added to the Fortified Products	Duration of Intervention	Outcomes
Marliyati 2016	[31]	Indonesia	controlled clinical trial	31	7–9 years	7–9 years	Non-branded cooking oil	Carotene from red palm oil	8 weeks	Se retinol; IgG level; sub-clinical vitamin A deficiency; dietary intake of energy, protein, fat, vitamin A; consumption of cooking oil; BMI; WAZ (results not reported); morbidity
Candelaria 2005	[27]	Philippines	randomized controlled trail	622	4–7 years	4–7 years	Coconut oil	Vitamin A	6 months	Se retinol; dietary intake of energy, protein, vitamin C, vitamin A; distribution of subjects by sources of vitamin A; sub-clinical vitamin A deficiency; WHZ (results not reported); proportion of children with low WHZ; morbidity; cooking practices; cooking oil use
Atalhi 2020	[28,29,30]	Morocco	randomized controlled trial	140	19–40 years	19–40 years	Soy oil	Vitamin A	6 months	Se retinol; retinol in breast milk; proportion of mothers with low concentrations of retinol in their breast milk; breast milk fat; CRP; proportion of mothers who consumed foods rich in vitamin A; morbidity (results not reported)
Keller 2020	[33]	Denmark	birth cohort study	193,803	during fetal life	NA	Margarine	Vitamin A	NA	incidence of type 2 diabetes mellitus
Donglan 2006	[32]	China	controlled clinical trial	174	9–11 years	9–11 years	Oil	Vitamin A	5 months	Se levels of vitamin A, IgA, IgG, IgM, complement C3

Se: serum; IgG: Immunoglobulin G; BMI: body mass index; WAZ: weight for age z-core; WHZ: weight-for-height z-scores; CRP: C-reactive-protein; IgA: Immunoglobulin A; IgM: Immunoglobulin M.

## Data Availability

Data described in the manuscript, will be made available upon request.

## Supplementary Materials

The following supporting information can be downloaded at: https://www.mdpi.com/article/10.3390/nu15245135/s1, Supplementary File S1: Search strategies; Supplementary File S2: Characteristics of excluded studies; Supplementary File S3: Characteristics of included studies; Supplementary File S4: Risk of bias assessment; Supplementary File S5: Effect of Vitamin A-fortified versus non-fortified oils and fats; Supplementary File S6: Effect of Vitamin D-fortified versus non-fortified oils and fats; Supplementary File S7: Grade assessment.

## References

[1] Keats E.C., Neufeld L.M., Garrett G.S., Mbuya M.N.N., Bhutta Z.A. (2019). Improved micronutrient status and health outcomes in low- and middle-income countries following large-scale fortification: Evidence from a systematic review and meta-analysis. Am. J. Clin. Nutr..

[2] Stephensen C.B. (2001). Vitamin A, infection, and immune function. Annu. Rev. Nutr..

[3] Carazo A., Macakova K., Matousova K., Krcmova L.K., Protti M., Mladenka P. (2021). Vitamin A Update: Forms, Sources, Kinetics, Detection, Function, Deficiency, Therapeutic Use and Toxicity. Nutrients.

[4] Vijayaraghavan K. (2018). National control programme against nutritional blindness due to vitamin A deficiency: Current status & future strategy. Indian J. Med. Res..

[5] Wirth J.P., Petry N., Tanumihardjo S.A., Rogers L.M., McLean E., Greig A., Garrett G.S., Klemm R.D., Rohner F. (2017). Vitamin A Supplementation Programs and Country-Level Evidence of Vitamin A Deficiency. Nutrients.

[6] Sherwin J.C., Reacher M.H., Dean W.H., Ngondi J. (2012). Epidemiology of vitamin A deficiency and xerophthalmia in at-risk populations. Trans. R. Soc. Trop. Med. Hyg..

[7] WHO Global prevalence of vitamin A deficiency in populations at risk 1995–2005: WHO global database on vitamin A deficiency. https://www.who.int/publications/i/item/9789241598019.

[8] Vanherwegen A.S., Gysemans C., Mathieu C. (2017). Regulation of Immune Function by Vitamin D and Its Use in Diseases of Immunity. Endocrinol. Metab. Clin. N. Am..

[9] DeLuca H.F. (2004). Overview of general physiologic features and functions of vitamin D. Am. J. Clin. Nutr..

[10] Roth D.E., Abrams S.A., Aloia J., Bergeron G., Bourassa M.W., Brown K.H., Calvo M.S., Cashman K.D., Combs G., De-Regil L.M. (2018). Global prevalence and disease burden of vitamin D deficiency: A roadmap for action in low- and middle-income countries. Ann. N. Y. Acad. Sci..

[11] Palacios C., Gonzalez L. (2014). Is vitamin D deficiency a major global public health problem?. J. Steroid Biochem. Mol. Biol..

[12] Kimball S.M., Holick M.F. (2020). Official recommendations for vitamin D through the life stages in developed countries. Eur. J. Clin. Nutr..

[13] Lips P., de Jongh R.T., van Schoor N.M. (2021). Trends in Vitamin D Status Around the World. JBMR Plus.

[14] Prentice A. (2013). Nutritional rickets around the world. J. Steroid Biochem. Mol. Biol..

[15] Amrein K., Scherkl M., Hoffmann M., Neuwersch-Sommeregger S., Kostenberger M., Tmava Berisha A., Martucci G., Pilz S., Malle O. (2020). Vitamin D deficiency 2.0: An update on the current status worldwide. Eur. J. Clin. Nutr..

[16] World Health Organization (2006). Guidelines on Food Fortification with Micronutrients. https://apps.who.int/iris/handle/10665/43412.

[17] FAO World Food and Agriculture: Statistical Yearbook 2021. Rome. 2021. FIGURE 23: World Production of Vegetable Oils, Main Commodities. https://www.fao.org/documents/card/en/c/cb4477en.

[18] Hombali A.S., Solon J.A., Venkatesh B.T., Nair N.S., Pena-Rosas J.P. (2019). Fortification of staple foods with vitamin A for vitamin A deficiency. Cochrane Database Syst. Rev..

[19] Dunlop E., Kiely M.E., James A.P., Singh T., Pham N.M., Black L.J. (2021). Vitamin D Food Fortification and Biofortification Increases Serum 25-Hydroxyvitamin D Concentrations in Adults and Children: An Updated and Extended Systematic Review and Meta-Analysis of Randomized Controlled Trials. J. Nutr..

[20] Mendu V.V.R., Nair K.P.M., Athe R. (2019). Systematic review and meta-analysis approach on vitamin A fortified foods and its effect on retinol concentration in under 10 year children. Clin. Nutr. ESPEN.

[21] Al Khalifah R., Alsheikh R., Alnasser Y., Alsheikh R., Alhelali N., Naji A., Al Backer N. (2020). The impact of vitamin D food fortification and health outcomes in children: A systematic review and meta-regression. Syst. Rev..

[22] Brandao-Lima P.N., Santos B.D.C., Aguilera C.M., Freire A.R.S., Martins-Filho P.R.S., Pires L.V. (2019). Vitamin D Food Fortification and Nutritional Status in Children: A Systematic Review of Randomized Controlled Trials. Nutrients.

[23] Souza S.V.S., Borges N., Vieira E.F. (2022). Vitamin D-fortified bread: Systematic review of fortification approaches and clinical studies. Food Chem..

[24] Gasparri C., Perna S., Spadaccini D., Alalwan T., Girometta C., Infantino V., Rondanelli M. (2019). Is vitamin D-fortified yogurt a value-added strategy for improving human health? A systematic review and meta-analysis of randomized trials. J. Dairy Sci..

[25] McGuinness L.A., Higgins J.P.T. (2020). Risk-of-bias VISualization (robvis): An R package and Shiny web app for visualizing risk-of-bias assessments. Res. Syn. Meth..

[26] Balshem H., Helfand M., Schünemann H.J., Oxman A.D., Kunz R., Brozek J., Vist G.E., Falck-Ytter Y., Meerpohl J., Norris S. (2011). GRADE guidelines: 3. Rating the quality of evidence. J. Clin. Epidemiol..

[27] Candelaria L.V., Magsadia C.R., Velasco R.E., Pedro M.R., Barba C.V., Tanchoco C.C. (2005). The effect of vitamin A-fortified coconut cooking oil on the serum retinol concentration of Filipino children 4–7 years old. Asia Pac. J. Clin. Nutr..

[28] Atalhi N., El Hamdouchi A., Barkat A., Elkari K., Hamrani A., El Mzibri M., Haskell M.J., Mokhtar N., Aguenaou H. (2020). Combined consumption of a single high-dose vitamin A supplement with provision of vitamin A fortified oil to households maintains adequate milk retinol concentrations for 6 months in lactating Moroccan women. Appl. Physiol. Nutr. Metab..

[29] Atalhi N., Choua G., Elhamdouchi A., Elhaloui N., Elmzibri M., Haskell M., Aguenaou H., Mokhtar N. (2011). Impact of daily consumption of Vitamin A fortified oil on human milk Vitamin A concentration in lactating Moroccan women. Ann. Nutr. Metab..

[30] (2015). PACTR201512001217212. Combined Consumption of a Single High-Dose Vitamin A Supplement and Provision of Vitamin A Fortified Oil to Households Maintains Adequate Milk Retinol. https://pactr.samrc.ac.za/TrialDisplay.aspx?TrialID=1217.

[31] Marliyati S.A., Martianto D., Andarwulan N., Fauzi S. (2016). Efficacy of non-branded cooking oil fortified with carotene from RPO on blood retinol and IgG of children aged 7–9 years. Pak. J. Nutr..

[32] Wang D.L., Xiao Q.M., Hong Y., Li S.T., Chen W.Q., Cheng Y.Y. (2006). Effects of vitamin A-fortified edible oil on improving the immune fuction of children. Acta Nutr. Sin..

[33] Keller A., Angquist L., Jacobsen R., Vaag A., Heitmann B.L. (2017). A retrospective analysis of a societal experiment among the Danish population suggests that exposure to extra doses of vitamin A during fetal development may lower type 2 diabetes mellitus (T2DM) risk later in life. Br. J. Nutr..

[34] Ghasemifard N., Nasimi N., Hassanzadeh-Rostami Z., Abbasi A., Faghih S. (2020). Effects of vitamin D fortified canola oil on vitamin D and lipid profiles in healthy adults: A double-blind randomized trial. Iran. J. Nutr. Sci. Food Technol..

[35] Ghasemifard N., Hassanzadeh-Rostami Z., Abbasi A., Naghavi A.M., Faghih S. (2022). Effects of vitamin D-fortified oil intake versus vitamin D supplementation on vitamin D status and bone turnover factors: A double blind randomized clinical trial. Clin. Nutr. ESPEN.

[36] (2018). Irct20180708040401N. Fortification and Supplementation Effect on Vitamin D Levels. https://trialsearch.who.int/Trial2.aspx?TrialID=IRCT20180708040401N1.

[37] Stougaard M., Damm P., Frederiksen P., Jacobsen R., Heitmann B.L. (2018). Extra vitamin D from fortification and the risk of preeclampsia: The D-tect Study. PLoS ONE.

[38] Moos C., Duus K.S., Frederiksen P., Heitmann B.L., Andersen V. (2020). Exposure to the Danish Mandatory Vitamin D Fortification Policy in Prenatal Life and the Risk of Developing Coeliac Disease-The Importance of Season: A Semi Ecological Study. Nutrients.

[39] Duus K.S., Moos C., Frederiksen P., Andersen V., Heitmann B.L. (2021). Prenatal and Early Life Exposure to the Danish Mandatory Vitamin D Fortification Policy Might Prevent Inflammatory Bowel Disease Later in Life: A Societal Experiment. Nutrients.

[40] Keller A., Stougard M., Frederiksen P., Thorsteinsdottir F., Vaag A., Damm P., Jacobsen R., Heitmann B.L. (2018). In Utero exposure to extra vitamin D from food fortification and the risk of subsequent development of gestational diabetes: The D-tect study. Nutr. J..

[41] (2012). NCT03330301. D-tecting Disease—From Exposure to Vitamin D During Critical Periods of Life. NCT03330301.

[42] Jacobsen R., Abrahamsen B., Bauerek M., Holst C., Jensen C.B., Knop J., Raymond K., Rasmussen L.B., Stougaard M., Sorensen T.I. (2013). The influence of early exposure to vitamin D for development of diseases later in life. BMC Public Health.

[43] Thorsteinsdottir F., Maslova E., Jacobsen R., Frederiksen P., Keller A., Backer V., Heitmann B.L. (2019). Exposure to Vitamin D Fortification Policy in Prenatal Life and the Risk of Childhood Asthma: Results From the D-Tect Study. Nutrients.

[44] Jacobsen R., Hypponen E., Sorensen T.I., Vaag A.A., Heitmann B.L. (2015). Gestational and Early Infancy Exposure to Margarine Fortified with Vitamin D through a National Danish Programme and the Risk of Type 1 Diabetes: The D-Tect Study. PLoS ONE.

[45] Jacobsen R., Moldovan M., Vaag A.A., Hypponen E., Heitmann B.L. (2016). Vitamin D fortification and seasonality of birth in type 1 diabetic cases: D-tect study. J. Dev. Orig. Health Dis..

[46] Handel M.N., Frederiksen P., Osmond C., Cooper C., Abrahamsen B., Heitmann B.L. (2017). Prenatal exposure to vitamin D from fortified margarine and risk of fractures in late childhood: Period and cohort results from 222,000 subjects in the D-tect observational study. Br. J. Nutr..

[47] Jensen C.B., Berentzen T.L., Gamborg M., Sorensen T.I., Heitmann B.L. (2014). Does prenatal exposure to vitamin D-fortified margarine and milk alter birth weight? A societal experiment. Br. J. Nutr..

[48] Jensen C.B., Gamborg M., Berentzen T.L., Sørensen T.I.A., Heitmann B.L. (2015). Prenatal exposure to vitamin-D from fortified margarine and milk and body size at age 7 years. Eur. J. Clin. Nutr..

[49] Stougaard M., Damm P., Frederiksen P., Jacobsen R., Heitmann B.L. (2018). Exposure to vitamin D from fortified margarine during fetal life and later risk of pre-eclampsia: The D-tect Study. Public Health Nutr..

[50] Bjelakovic G., Gluud L.L., Nikolova D., Whitfield K., Wetterslev J., Simonetti R.G., Bjelakovic M., Gluud C. (2014). Vitamin D supplementation for prevention of mortality in adults. Cochrane Database Syst. Rev..

[51] Black L.J., Seamans K.M., Cashman K.D., Kiely M. (2012). An updated systematic review and meta-analysis of the efficacy of vitamin D food fortification. J. Nutr..

[52] Nikooyeh B., Neyestani T.R. (2022). The effects of vitamin D-fortified foods on circulating 25(OH)D concentrations in adults: A systematic review and meta-analysis. Br. J. Nutr..

[53] Rohner F., Wirth J.P., Zeng W., Petry N., Donkor W.E.S., Neufeld L.M., Mkambula P., Groll S., Mbuya M.N., Friesen V.M. (2023). Global Coverage of Mandatory Large-Scale Food Fortification Programs: A Systematic Review and Meta-Analysis. Adv. Nutr..

[54] Stevens G.A., Beal T., Mbuya M.N.N., Luo H., Neufeld L.M. (2022). Micronutrient deficiencies among preschool-aged children and women of reproductive age worldwide: A pooled analysis of individual-level data from population-representative surveys. Lancet. Glob. Health.

[55] Van Schoor N., Lips P. (2017). Global Overview of Vitamin D Status. Endocrinol. Metab. Clin. N. Am..

[56] Cianferotti L., Marcocci C. (2012). Subclinical vitamin D deficiency. Best Pract. Res. Clin. Endocrinol. Metab..

[57] Mogire R.M., Mutua A., Kimita W., Kamau A., Bejon P., Pettifor J.M., Adeyemo A., Williams T.N., Atkinson S.H. (2020). Prevalence of vitamin D deficiency in Africa: A systematic review and meta-analysis. Lancet. Glob. Health.

[58] Bartley J. (2010). Vitamin D: Emerging roles in infection and immunity. Expert Rev. Anti-Infect. Ther..

