# Supplementary material for: Benefits and Harms of Edible Vegetable Oils and Fats Fortified with Vitamins A and D as a Public Health Intervention in the General Population: A Systematic Review of Interventions
Source: Nutrients. 2023 Dec 18;15(24):5135. doi: 10.3390/nu15245135 (PMC10745565; doi:10.3390/nu15245135)
Supplement: Supplementary file 1 [file nutrients-15-05135-s001.zip › Supplementary File S1_Search_strategies.pdf]

Supplementary file S1: Search strategies

Medline Search Strategy (Ovid)

1. exp Vitamin A/
2. (vitamin A* or retinol* or caroten* or retinyl*).tw.
3. exp Vitamin D/
4. (vitamin D* or calciferol* or cholecalciferol* or colecalciferol* or ergocalciferol* or calciol*).tw.
5. or/1-4
6. Fats/
7. Dietary Fats/
8. exp Fats, Unsaturated
9. exp Dietary Fats, Unsaturated/
10. (fat or fats).tw.
11. exp Oils/
12. exp Fish Oils/
13. exp Plant Oils/
14. (oil or oils).tw.
15. Margarine/
16. (margarine*).tw.
17. Ghee/
18. (ghee or suet or oleo or lard or tallow or blubber).tw.
19. or/6-18
20. 5 and 19
21. Food, Fortified/
22. (fortif* or enrich* or enhanc*).tw.
23. ((boost* or increas* or improv* or add*) adj3 (vitamin A* or retinol* or caroten* or retinyl* or vitamin D* or calciferol* or cholecalciferol* or colecalciferol* or ergocalciferol* or calciol*)).tw.
24. or/21-23
25. 20 and 24

[Cochrane RCT Filter 2022 (100), high sensitivity – without “drug therapy.fs”]

26. randomized controlled trial.pt.
27. controlled clinical trial.pt.
28. randomi?ed.ab.
29. placebo.ab.
30. randomly.ab.
31. trial.ab.
32. groups.ab.
33. or/26-32
34. exp animals/ not humans/
35. 33 not 34
36. 25 and 35

[Waffenschmidt 2020 (101), sensitivity max. version + time series.mp]

37. exp cohort studies/ or exp epidemiologic studies/ or exp clinical trial/ or exp evaluation studies as topic/ or exp statistics as topic/
38. ((control and (group* or study)) or (time and factors) or program or survey* or ci or cohort or comparative stud* or evaluation studies or follow-up* or time series).mp.
39. or/37–38
40. (animals/not humans/) or comment/or editorial/ or exp review/ or meta analysis/ or consensus/ or exp guideline/
41. hi.fs. or case report.mp.
42. or/40-41
43. 39 not 42
44. 25 and 43
45. 36 or 44

Cochrane Central Register of Controlled Trials (Cochrane Register of Studies Online)

1. MESH DESCRIPTOR Vitamin A EXPLODE ALL TREES
2. (vitamin A* OR retinol* OR caroten* OR retinyl*):TI,AB,KY
3. MESH DESCRIPTOR Vitamin D EXPLODE ALL TREES
4. (vitamin D* OR calciferol* OR cholecalciferol* OR colecalciferol* OR ergocalciferol* OR calciol*):TI,AB,KY
5. #1 OR #2 OR #3 OR #4
6. MESH DESCRIPTOR Fats
7. MESH DESCRIPTOR Dietary Fats
8. MESH DESCRIPTOR Fats, Unsaturated EXPLODE ALL TREES
9. MESH DESCRIPTOR Dietary Fats, Unsaturated EXPLODE ALL TREES
10. (fat OR fats):TI,AB,KY
11. MESH DESCRIPTOR Oils EXPLODE ALL TREES
12. MESH DESCRIPTOR Fish Oils EXPLODE ALL TREES
13. MESH DESCRIPTOR Plant Oils EXPLODE ALL TREES
14. (oil OR oils):TI,AB,KY
15. MESH DESCRIPTOR Margarine
16. (margarine*):TI,AB,KY
17. MESH DESCRIPTOR Ghee
18. (ghee OR suet OR oleo OR lard OR tallow OR blubber):TI,AB,KY
19. #6 OR #7 OR #8 OR #9 OR #10 OR #11 OR #12 OR #13 OR #14 OR #15 OR #16 OR #17 OR #18
20. #5 AND #19
21. MESH DESCRIPTOR Food, Fortified
22. (fortif* OR enrich* OR enhanc*):TI,AB,KY
23. ((boost* OR increas* OR improv* OR add*) ADJ3 (vitamin A* OR retinol* OR caroten* OR retinyl* OR vitamin D* OR calciferol* OR cholecalciferol* OR colecalciferol* OR ergocalciferol* OR calciol*)):TI,AB,KY
24. #21 OR #22 OR #23
25. #20 AND #24

Scopus (www.scopus.com – Advanced search)

TITLE-ABS-KEY((("vitamin A*" OR retinol* OR caroten* OR retinyl* OR "vitamin D*" OR calciferol* OR cholecalciferol* OR colecalciferol* OR ergocalciferol* OR calciol*) AND (fat OR fats OR oil OR oils OR margarine* OR ghee OR suet OR oleo OR lard OR tallow OR blubber)) AND (fortif* OR enrich* OR enhanc* OR ((boost* OR increas* OR improv* OR add*) W/3 ("vitamin A*" OR retinol* OR caroten* OR retinyl* OR "vitamin D*" OR calciferol* OR cholecalciferol* OR colecalciferol* OR ergocalciferol* OR calciol*)))) AND TITLE-ABS((random* OR placebo OR trial OR groups OR (control* AND (group* OR study)) OR (time AND factors) OR program OR survey* OR cohort OR comparative stud* OR "follow-up*" OR multicenter OR "multi center" OR multicentre OR "multi centre" OR quasiexperiment* OR "quasi experiment*" OR evaluat* OR "time series" OR "time point" OR "time points" OR "repeated measur*" OR (before W/5 after) OR (pre W/5 post) OR ((pretest OR "pre test") AND (posttest OR "post test")) OR intervention OR interventions OR effect OR effects OR impact OR impacts OR controlled))

Global Index Medicus (WHO) - https://www.globalindexmedicus.net/

All indexes, All information sources

("vitamin A*" OR retinol* OR caroten* OR retinyl* OR "vitamin D*" OR calciferol* OR cholecalciferol* OR colecalciferol* OR ergocalciferol* OR calciol*) AND (fat OR fats OR oil OR oils OR margarine* OR ghee OR suet OR oleo OR lard OR tallow OR blubber)

AND (fortif* OR enrich* OR enhanc* OR ((boost* OR increas* OR improv* OR add*) AND ("vitamin A*" OR retinol* OR caroten* OR retinyl* OR "vitamin D*" OR calciferol* OR cholecalciferol* OR colecalciferol* OR ergocalciferol* OR calciol*)))

ICTRP (Standard search)

("vitamin A" OR retinol* OR caroten* OR retinyl* OR "vitamin D" OR "vitamin D3" OR calciferol* OR cholecalciferol* OR colecalciferol* OR ergocalciferol* OR calciol*) AND (fat OR fats OR oil OR oils OR margarine* OR ghee OR suet OR oleo OR lard OR tallow OR blubber)

ClinicalTrials.gov (Expert search)

(fortified OR fortification OR fortificant OR enriched OR enhanced OR boost OR boosted OR increase OR increased OR improve OR improved OR add OR added) AND ("vitamin A" OR retinol OR carotene OR retinyl OR "vitamin D" OR "vitamin D3" OR calciferol OR cholecalciferol OR colecalciferol OR ergocalciferol OR calciol) AND (fat OR fats OR oil OR oils OR margarine OR margarines OR ghee OR suet OR oleo OR lard OR tallow OR blubber)
