# Supplementary material for: Benefits and Harms of Edible Vegetable Oils and Fats Fortified with Vitamins A and D as a Public Health Intervention in the General Population: A Systematic Review of Interventions
Source: Nutrients. 2023 Dec 18;15(24):5135. doi: 10.3390/nu15245135 (PMC10745565; doi:10.3390/nu15245135)
Supplement: Supplementary file 1 [file nutrients-15-05135-s001.zip › Supplementary File S2_Excluded_studies.pdf]

Supplementary file S2: Characteristics of excludes studies

Study	Reason for exclusion
Bromage 2018 (1)	Wrong intervention (modelled fortification)
Calvo 2005 (2)	Wrong study design (review paper)
Cashman 2015 (3)	Wrong study design
ChiCTR-TRC-12001914 (4)	Wrong intervention (supplementation)
CTRI/2020/11/029135 (5)	Wrong study design (beta-carotene bioavailability study)
Dass 2021 (6)	Wrong intervention (micronutrient fortification, biofortification, supplementation and use of locally available nutrient-rich foods)
deJong 2022 (7)	Wrong study design (cross-sectional study)
DeOliveira 1994 (8)	Wrong study design (simple before-and-after study)
Diósady 2013 (9)	Wrong study design (review paper)
Dunnigan 1986 (10)	Wrong intervention (fortification of margarine, butter and milk)
Dutra-de-Oliveira 1998 (11)	Wrong comparator (All participants consumed fortified soybean oil. Effect of heating was investigated, i.e. outcome was compared in heated or non-heated groups)
Engle-Stone 2017 (12)	Wrong study design (repeated cross-sectional study)
Engle-Stone 2015 (13)	Wrong study design
Englestone 2015 (14)	Wrong intervention (supplementation)
Fiedler 2010 (15)	Wrong study design
Fiedler 2015 (16)	Wrong study design (cross-sectional study)
Gibbs 2015 (17)	Wrong study design (cross-sectional study)
Harika 2016 (18)	Wrong study design (simulation study)
Ianiro 2013 (19)	Wrong intervention (oil fortified with probiotics or antioxidants)
Jaaskelainen 2017 (20)	Wrong intervention (wrong vehicle: fortified fat spreads, fluid milk products and respective lactose-free milk-, soy-, and cereal-based drinks)
Jensen 2020 (21)	Wrong population (women diagnosed with infertility; The question investigated was, whether infertile women exposed to vitamin D fortified margarine have an increased chance of live birth or not)
Kozlowska-Wojciechowska 2002 (22)	Wrong intervention (butter vs. margarine consumption)
Laaksi 2006 (23)	Wrong intervention (liquid milk products, margarines and butter were fortified)
Lehtonen-Veromaa 2008 (24)	Wrong intervention (fluid milks, margarines and spreads were fortified)
Lietz 2001 (25)	Wrong intervention (No fortification)
Lopriore 2004 (26)	Wrong intervention (Spread fortified with vitamins and minerals)
Madkour 1994 (27)	Wrong intervention (vitamin A and E fortified palm oil vs. plain oil)
Mark 2019 (28)	Wrong study design
Martianto 2015 (29)	Wrong study design
Masni 2016 (30)	Wrong intervention (red palm oil emulsion)
Mason 2011 (31)	Wrong comparator (vitamin A fortification compared to vitamin A supplementation)
Mazzanti 2015 (32)	Wrong intervention (Extra virgin olive oil enriched with vitamin K1, vitamin D3 and vitamin B6, compared to extra virgin olive oil without added vitamins)
Muzhingi 2017 (33)	Wrong intervention (vehicle was kale)

Nair 1993 (34)	Wrong intervention (vitamin E-fortified fish oil)
NCT01669915 (35)	Wrong intervention (vitamin D + fish oil supplementation)
NCT03499327 (36)	Wrong intervention (UV-treated vs. untreated oil)
NCT03826654 (37)	Wrong study design (Cluster RCT with only 2 clusters; Nikooyeh 2020)
NCT04156074 (38)	Wrong intervention (supplementation)
NCT04302987 (39)	Wrong intervention (supplementation)
NCT05271045 (40)	Wrong population (adults with type 2 diabetes)
NCT05460221 (41)	Wrong intervention (Optimized extra virgin olive oil (with bioactive compounds or polyphenols)
NCT05541094 (42)	Wrong intervention (fortified vs. unfortified milk + oil)
Nikooyeh 2020 (43)	Wrong study design (Cluster RCT with only 2 clusters)
Olsen 2016 (44)	Wrong intervention (supplementation)
Parr 2018 (45)	Wrong study design (not fortification effects were investigated)
Piirainen 2007 (46)	Wrong intervention (wrong vehicle: Both milks and margarines were fortified)
Raulio 2017 (47)	Wrong intervention (liquid dairy products and fat spreads were fortified)
Rohner 2016 (48)	Wrong study design (cross-sectional study)
Salam 2021 (49)	Wrong comparator (Cooking Oil Fortification vs. Nutrition education vs. supplementation)
Sandjaja 2015 (50)	Wrong study design (simple before-after study)
Sandjaja 2015 (51)	Wrong study design
Sauvant 2003 (52)	Wrong intervention (supplementation)
Schmaelzle 2014 (53)	Wrong intervention (There were three treatment groups, which received orange maize and placebo oil, white maize and placebo oil, or white maize and a daily vitamin A supplement)
Schoener 2019 (54)	Wrong study design (vitamin D bioaccessibility was measured)
Shakur 2014 (55)	Wrong study design (the modelled effect of increased vitamin D fortification was examined)
Sichert-Hellert 2000 (56)	Wrong study design
Sivan 2002 (57)	Wrong intervention (fortification at the place of use)
Sivan 2001 (58)	Wrong intervention (red palm oil supplementation)
Smedshaug 2007 (59)	Wrong intervention (supplementation)
Soekirman 2012 (60)	Wrong study design
Solon 1996 (61)	Wrong intervention (Wrong fortificant. The intervention margarine was fortified with vitamins A + D + B1 and was compared to a non-fortified margarine (containing no vitamins A, D, or B1).
Solon 1998 (62)	Wrong study design (review paper)
Tanumihardjo 2015 (63)	Wrong study design (review paper)
Touaoro 2015 (64)	Wrong study design (cross-sectional study)
Unlu 2005 (65)	Wrong intervention
vanDam 2007 (66)	Wrong study design (cross-sectional study)
Walters 2019 (67)	Wrong study design (economic evaluation)

References:

1. Bromage S, Ganmaa D, Rich-Edwards JW, Rosner B, Bater J, Fawzi WW. Projected effectiveness of mandatory industrial fortification of wheat flour, milk, and edible oil with multiple micronutrients among Mongolian adults. *PLoS One*. 2018;13(8):e0201230.

2. Calvo MS, Whiting SJ, Barton CN. Vitamin D intake: a global perspective of current status. *Journal of Nutrition*. 2005;135(2):310-6.
3. Cashman KD, Kazantzidis A, Webb AR, Kiely M. An Integrated Predictive Model of Population Serum 25-Hydroxyvitamin D for Application in Strategy Development for Vitamin D Deficiency Prevention1-3. *Journal of Nutrition*. 2015;145(10):2419-25.
4. Chi CT, Nanjing Medical University School of Public Health N. Effects of Vitamin A Supplementation to Lactating Mothers on Infants' Antibody Response to Hepatitis B Vaccine in China. 2012.
5. CTRI/2020/11/029135. Beta carotene absorption and vitamin A status in humans. 2020; Available from: http://www.ctri.nic.in/Clinicaltrials/pmaindet2.php?trialid=49304.
6. Dass M, Nyako J, Tortoe C, Fanou-Fogny N, Nago E, Hounhouigan J et al. Comparison of Micronutrient Intervention Strategies in Ghana and Benin to Cover Micronutrient Needs: Simulation of Bene-Fits and Risks in Women of Reproductive Age. *Nutrients*. 2021;13(7):01.
7. de Jong MH, Nawijn EL, Verkaik-Kloosterman J. ^{Contribution of fortified margarines and other plant-based fats to micronutrient intake in the Netherlands}. *European Journal of Nutrition*. 2022;61(4):1893-904.
8. De Oliveira JED, Desai ID, Favaro RMD, Ferreira JF. Effect of heat treatment during cooking on the biological value of vitamin a fortified soybean oil in human. *International journal of food sciences and nutrition*. 1994;45(3):203-7.
9. Diósady LL, Venkatesh-Mannar MG. Vitamin A fortification of cooking oils. *Handbook of Food Fortification and Health: From Concepts to Public Health Applications*. 2013;1:275-90.
10. Dunnigan MG, Fraser SA, McIntosh WB, Moseley H, Sumner DJ. The prevention of vitamin D deficiency in the elderly. *Scottish Medical Journal*. 1986;31(3):144-9.
11. Dutra-de-Oliveira JE, Leonardo IR, Jordão AA, Jr., Vannucchi H, Duarte Fávaro RM. Absorption, by Humans, of β-carotene from fortified soybean oil added to rice: Effect of heat treatment. *Journal of the American College of Nutrition*. 1998;17(4):361-5.
12. Engle-Stone R, Nankap M, Ndjebayi A, Gimou MM, Friedman A, Haskell MJ et al. Vitamin A Status of Women and Children in Yaounde and Douala, Cameroon, is Unchanged One Year after Initiation of a National Vitamin A Oil Fortification Program. *Nutrients*. 2017;9(5):20.
13. Engle-Stone R, Ndjebayi A, Nankap M, Gimou M-M, Friedman A, Brown K. Vitamin A and Iron Status of Women and Young Children in Major Cities of Cameroon before and after Vitamin A Fortification of Vegetable Oil and Iron Fortification of Wheat Flour. 2015.
14. Englestone R, Osei A, Reario D, Arsenault J, Brown K, Haskell M. Short-term, Daily Supplementation with Vitamin A, But Not Beta-carotene, Increases Plasma and Breast Milk Retinol Concentration in Lactating Filipino Women. 2015.
15. Fiedler JL, Afidra R. Vitamin A fortification in Uganda: comparing the feasibility, coverage, costs, and cost-effectiveness of fortifying vegetable oil and sugar. *Food & Nutrition Bulletin*. 2010;31(2):193-205.
16. Fiedler JL, Lividini K, Bermudez OI. Estimating the impact of vitamin A-fortified vegetable oil in Bangladesh in the absence of dietary assessment data. *Public health nutrition*. 2015;18(3):414-20.
17. Gibbs M, Samuel A, Wuehler S, Tesfaye K, Gibson R. Modelling the Impact of Fortification on the Prevalence of Inadequate Micronutrient Intakes in Ethiopia: Results from the Ethiopian National Food Consumption Survey. 2015.
18. Harika RK, Dotsch-Klerk M, Zock PL, Eilander A. Compliance with Dietary Guidelines and Increased Fortification Can Double Vitamin D Intake: A Simulation Study. *Annals of nutrition & metabolism*. 2016;69(3-4):246-55.
19. Ianiro G, Pizzoferrato M, Franceschi F, Tarullo A, Luisi T, Gasbarrini G. Effect of an extra-virgin olive oil enriched with probiotics or antioxidants on functional dyspepsia: a pilot study. *European review for medical and pharmacological sciences*. 2013;17(15):2085-90.
20. Jaaskelainen T, Itkonen ST, Lundqvist A, Erkkola M, Koskela T, Lakkala K et al. The positive impact of general vitamin D food fortification policy on vitamin D status in a representative adult

Finnish population: evidence from an 11-y follow-up based on standardized 25-hydroxyvitamin D data. *American Journal of Clinical Nutrition*. 2017;105(6):1512-20.
21. Jensen A, Nielsen ML, Guleria S, Kjaer SK, Heitmann BL, Kesmodel US. Chances of live birth after exposure to vitamin D-fortified margarine in women with fertility problems: results from a Danish population-based cohort study. *Fertility & Sterility*. 2020;113(2):383-91.
22. Kozlowska-Wojciechowska M, Makarewicz-Wujec M, Nowicka G. [Increased serum levels of vitamin D and calcium in young men after replacement of butter with soft margarine in usual diet]. *Polskie Archiwum Medycyny Wewnetrznej*. 2002;108(4):953-8.
23. Laaksi IT, Ruohola JPS, Ylikomi TJ, Auvinen A, Haataja RI, Pihlajamäki HK et al. Vitamin D fortification as public health policy: Significant improvement in vitamin D status in young Finnish men. *European journal of clinical nutrition*. 2006;60(8):1035-8.
24. Lehtonen-Veromaa M, Mottonen T, Leino A, Heinonen OJ, Rautava E, Viikari J. Prospective study on food fortification with vitamin D among adolescent females in Finland: minor effects. *British journal of nutrition*. 2008;100(2):418-23.
25. Lietz G, Henry CJ, Mulokozi G, Mugyabuso JK, Ballart A, Ndossi GD et al. Comparison of the effects of supplemental red palm oil and sunflower oil on maternal vitamin A status. *American Journal of Clinical Nutrition*. 2001;74(4):501-9.
26. Lopriore C, Guidoum Y, Briend A, Branca F. Spread fortified with vitamins and minerals induces catch-up growth and eradicates severe anemia in stunted refugee children aged 3-6 y. *American Journal of Clinical Nutrition*. 2004;80(4):973-81.
27. M.A M. Plain dietary palm oil versus vitamin A and E fortified form histological and biochemical studies on liver and kidney. 1994. p. 207-28.
28. Mark HE, Assiene JG, Luo H, Nankap M, Ndjebayi A, Ngnie-Teta I et al. Monitoring of the National Oil and Wheat Flour Fortification Program in Cameroon Using a Program Impact Pathway Approach. *Current Developments in Nutrition*. 2019;3(8):nzz076.
29. Martianto D, Natakusuma S, Rahardjo S, Jus'at I, Soekarjo D, Pribadi N et al. Quality Control to Enable Successful Vitamin A Fortification of Cooking Oil in Indonesia - A Pilot Program in Two Rural Districts. 2015.
30. Masni, Sirajuddin S, Syaharuddin, Syam A. Influence of a red palm oil emulsion on the level of retinol in the plasma of primary school children in the coastal area of Makassar city. *Pakistan journal of nutrition*. 2016;15(5):465-73.
31. Mason JB, Ramirez MA, Fernandez CM, Pedro R, Lloren T, Saldanha L et al. Effects on vitamin A deficiency in children of periodic high-dose supplements and of fortified oil promotion in a deficient area of the Philippines. *International Journal for Vitamin & Nutrition Research*. 2011;81(5):295-305.
32. Mazzanti L, Battino M, Nanetti L, Raffaelli F, Alidori A, Sforza G et al. Effect of 1-year dietary supplementation with vitaminized olive oil on markers of bone turnover and oxidative stress in healthy post-menopausal women. *Endocrine*. 2015;50(2):326-34.
33. Muzhingi T, Yeum KJ, Bermudez O, Tang G, Siwela AH. Peanut butter increases the bioavailability and bioconversion of kale Î²-carotene to vitamin A. *Asia Pacific journal of clinical nutrition*. 2017;26(6):1039-47.
34. Nair PP, Judd JT, Berlin E, Taylor PR, Shami S, Sainz E et al. Dietary fish oil-induced changes in the distribution of α-tocopherol, retinol, and β-carotene in plasma, red blood cells, and platelets: Modulation by vitamin E. *American Journal of Clinical Nutrition*. 1993;58(1):98-102.
35. Nct, Brigham, Women's Hospital N, National Institute on A. A Large Randomized Trial of Vitamin D, Omega-3 Fatty Acids and Cognitive Decline. 2012.
36. NCT03499327. Human Intervention Study to Increase 25-hydroxyvitamin D Levels. 2018; Available from: https://clinicaltrials.gov/show/NCT03499327.
37. NCT03826654. Assessment of the Efficacy of Vitamin D-fortified Oil in Healthy Adults. 2019; Available from: https://clinicaltrials.gov/show/NCT03826654.
38. NCT04156074. Innovative Food Structures to Enhance Nutrient Bioavailability. 2019; Available from: https://clinicaltrials.gov/show/NCT01381276.

39. NCT04302987. Vitamin D Intervention in Infants - 6 Years Follow-up (VIDI2). 2019; Available from: https://ClinicalTrials.gov/show/NCT04302987.

40. NCT05271045. Assessment of the Efficacy of Vitamins A and D and γ-oryzanol-fortified Canola Oil in Adults With Type 2 Diabetes. 2021; Available from: https://ClinicalTrials.gov/show/NCT05271045.

41. Universidad de G, Complejo Hospitalario Universitario de S, Hospital Universitario Reina Sofia de C. Antihypertensive and Cardioprotective Evaluation of a Functional Olive Oil. https://ClinicalTrials.gov/show/NCT05460221; 2022.

42. Dr. Anuradha K, Trusts T, Hirabai Cowasji Jehangir Medical Research I. Study of Vitamin D Fortification of Milk and Oil. https://ClinicalTrials.gov/show/NCT05541094; 2020.

43. Nikooyeh B, Zargaraan A, Kalayi A, Shariatzadeh N, Zahedirad M, Jamali A et al. Vitamin D-fortified cooking oil is an effective way to improve vitamin D status: an institutional efficacy trial. *European Journal of Nutrition*. 2020;59(6):2547-55.

44. Olsen KE, Binkley N, Gannon BM, Tanumihardjo SA. Serum bone-building metabolites are enhanced by a restricted vitamin A intervention in Zambian children with high liver reserves of vitamin A. *FASEB journal Conference: experimental biology 2016, EB San diego, CA united states Conference start: 20160402 Conference end: 20160406 Conference publication: (varpagings)*. 2016;30(no pagination).

45. Parr CL, Magnus MC, Karlstad O, Holvik K, Lund-Blix NA, Haugen M et al. Vitamin A and D intake in pregnancy, infant supplementation, and asthma development: the Norwegian Mother and Child Cohort. *American Journal of Clinical Nutrition*. 2018;107(5):789-98.

46. Piirainen T, Laitinen K, Isolauri E. Impact of national fortification of fluid milks and margarines with vitamin D on dietary intake and serum 25-hydroxyvitamin D concentration in 4-year-old children. *European journal of clinical nutrition*. 2007;61(1):123-8.

47. Raulio S, Erlund I, Mannisto S, Sarlio-Lahteenkorva S, Sundvall J, Tapanainen H et al. Successful nutrition policy: improvement of vitamin D intake and status in Finnish adults over the last decade. *European Journal of Public Health*. 2017;27(2):268-73.

48. Rohner F, Raso G, Ake-Tano SO, Tschannen AB, Mascie-Taylor CG, Northrop-Clewes CA. The Effects of an Oil and Wheat Flour Fortification Program on Pre-School Children and Women of Reproductive Age Living in Cote d'Ivoire, a Malaria-Endemic Area. *Nutrients*. 2016;8(3):148.

49. Salam A, Briawan D, Martianto D, Thaha AR, Virani D. Effect of vitamin a supplementation, cooking oil fortification, and nutrition education to postpartum mother on breast milk retinol levels. *Open Access Macedonian Journal of Medical Sciences*. 2021;9:823-7.

50. Sandjaja, Jusat I, Jahari AB, Ifrad, Htet MK, Tilden RL et al. Vitamin A-fortified cooking oil reduces Vitamin A deficiency in infants, young children and women: Results from a programme evaluation in Indonesia. *Public health nutrition*. 2015;18(14):2511-22.

51. Sandjaja S, Jus'at I, Jahari AB, Tilden R, Ernawati F, Soekarjo D et al. Fortifying Cooking Oil with Vitamin A in Two Rural Districts of Indonesia: Impact on Vitamin A Status of Mothers and Children. 2015.

52. Sauvant P, Mekki N, Charbonnier M, Portugal H, Lairon D, Borel P. Amounts and types of fatty acids in meals affect the pattern of retinoids secreted in human chylomicrons after a high-dose preformed vitamin A intake. *Metabolism: Clinical & Experimental*. 2003;52(4):514-9.

53. Schmaelzle S, Kaliwile C, Arscott SA, Gannon B, Masi C, Tanumihardjo SA. Nutrient and nontraditional food intakes by Zambian children in a controlled feeding trial. *Food and nutrition bulletin*. 2014;35(1):60-7.

54. Schoener AL, Zhang R, Lv S, Weiss J, McClements DJ. Fabrication of plant-based vitamin D₃-fortified nanoemulsions: influence of carrier oil type on vitamin bioaccessibility. *Food & function*. 2019;10(4):1826-35.

55. Shakur YA, Lou W, L'Abbe MR. Examining the effects of increased vitamin D fortification on dietary inadequacy in Canada. *Canadian Journal of Public Health Revue Canadienne de Sante Publique*. 2014;105(2):e127-32.

56. Sichert-Hellert W, Kersting M, Alexy U, Manz F. Ten-year trends in vitamin and mineral intake from fortified food in German children and adolescents. *European journal of clinical nutrition*. 2000;54(1):81-6.
57. Sivan YS, Alwin Jayakumar Y, Arumughan C, Sundaresan A, Jayalekshmy A, Suja KP et al. Impact of vitamin A supplementation through different dosages of red palm oil and retinol palmitate on preschool children. *Journal of tropical pediatrics*. 2002;48(1):24-8.
58. Sivan YS, Jayakumar YA, Arumughan C, Sundaresan A, Balachandran C, Job J et al. Impact of β-carotene supplementation through red palm oil. *Journal of tropical pediatrics*. 2001;47(2):67-72.
59. Smedshaug GB, Pedersen JI, Meyer HE. Can vitamin D supplementation improve grip strength in elderly nursing home residents? A double-blinded controlled trial. *Scandinavian journal of food and nutrition*. 2007;51(2):74-8.
60. Soekirman, Soekarjo D, Martianto D, Laillou A, Moench-Pfanner R. Fortification of Indonesian unbranded vegetable oil: public-private initiative, from pilot to large scale. *Food & Nutrition Bulletin*. 2012;33(4 Suppl):S301-9.
61. Solon FS, Solon MS, Mehansho H, West Jr KP, Sarol J, Perfecto C et al. Evaluation of the effect of vitamin A-fortified margarine on the vitamin A status of preschool Filipino children. *European journal of clinical nutrition*. 1996;50(11):720-3.
62. Solon FS. History of fortification of margarine with vitamin a in the Philippines. *Food and nutrition bulletin*. 1998;19(2):154-8.
63. Tanumihardjo SA, Gannon BM, Kaliwile C, Chileshe J. Hypercarotenodermia in Zambia: which children turned orange during mango season? *European journal of clinical nutrition*. 2015;69(12):1346-9.
64. Touaoro Z, Somda JC, Topan F, Yago-Wienne F, Sablah M. Evaluation of the Population Coverage of Mandatory Vitamin A Fortified Vegetable Oil in Burkina Faso. 2015.
65. Unlu NZ, Bohn T, Clinton SK, Schwartz SJ. Carotenoid absorption from salad and salsa by humans is enhanced by the addition of avocado or avocado oil. *Journal of Nutrition*. 2005;135(3):431-6.
66. van Dam RM, Snijder MB, Dekker JM, Stehouwer CD, Bouter LM, Heine RJ et al. Potentially modifiable determinants of vitamin D status in an older population in the Netherlands: the Hoorn Study. *American Journal of Clinical Nutrition*. 2007;85(3):755-61.
67. Walters D, Ndau E, Saleh N, Mosha T, Horton S. Cost-effectiveness of sunflower oil fortification with vitamin A in Tanzania by scale. *Maternal & Child Nutrition*. 2019 May;15 Suppl 3:e12720.
