# Supplementary material for: Benefits and Harms of Edible Vegetable Oils and Fats Fortified with Vitamins A and D as a Public Health Intervention in the General Population: A Systematic Review of Interventions
Source: Nutrients. 2023 Dec 18;15(24):5135. doi: 10.3390/nu15245135 (PMC10745565; doi:10.3390/nu15245135)
Supplement: Supplementary file 1 [file nutrients-15-05135-s001.zip › Supplementary File S3_Included_studies.pdf]

Supplementary file S3: Characteristics of included studies

		Description	
**Study Characteristics**	**Study ID**	#2604	
	**Author, date**	**Marliyati, 2016**	
	**Publication type** *(e.g. full report, abstract, letter)*	Full report	
	**Language of publication**	English	
	**Funding**	“to thank the Directorate General of Higher Education, Ministry of national Education, Republic of Indonesia for funding this research through the operational expenses of Bogor Agricultural University”	
	**Conflict** *(Notable conflicts of interest of study authors)*	not mentioned	
**Methods**	**Aim of study**	“The general objective of this research was to assess the efficacy of non-branded cooking oil fortified with carotene from RPO (red palm oil) on blood retinol and IgG of children aged 7-9 years.”, “This research aimed to assess the efficacy of non-branched cooking oil fortified with carotene from red palm oil (RPO) on blood retinol and IgG level.	
	**Study design** *(RCT/ CCT/ cohort/ controlled before-after/ interrupted time series)*	Quasi experimental pre- and post-treatment-controlled control trial	
	**Unit of allocation** *(by individuals, cluster/ groups or body parts)*	individuals<br>31 elementary school children aged 7-9 years, divided into two groups: RPO (red palm oil) group and control group	
	**Number of study arms**	2	
	**Start date of study**	not mentioned	
	**End date of study**	not mentioned	
**Participants**	**Population description** *(from which study participants are drawn)*	- Elementary school children, aged 7-9 years,<br>- healthy (based on the results of the doctor’s examination),	
	**Country where trial was performed**	Angsana village (1), Leuwiliang (2) Sub-district located in Bogor Regency, Indonesia	
	**Location/Setting**	Indonesia	
	**Inclusion criteria**	- Elementary school children, aged 7-9 years,<br>- Healthy (based on the results of the doctor’s examination),<br>- Received the explanation about the research,<br>- Sign informed consent,<br>- Agree to follow the research procedure	
	**Exclusion criteria**	not mentioned	
	**Number of participants**	Number of contacted persons	Not mentioned
		Agreed to participate	31
		Sample size *(started the study)*	14 boys (45,2%), 17 girls (54,8%)
		Lost to follow-up/withdrawals/dropouts	
		Number of participants analysed	RPO group n=15 (9 boys /60%/, 6 girls /40%/)<br>Control group: n=15 (5 boys /31,3%/, 11 girls /68,7%/)
		Sample size of total group	31
		Sample size of each group	RPO=15, Control=15
	**Sex** *(Male/Female; Boys/Girls)*	boys and girls	
	**Mean age (age range)**	-Both research groups most of the subject aged 8<br>-53,3% RPO, 50% Control group;<br>-average age 7.8 ± 0.7 years in both groups	
	**Race/Ethnicity**	not mentioned (Indonesia)	
	**Other relevant baseline data** *(e.g. BMI, health status, pregnant ect.)*	Characteristics of elementary school children and their families including the children’s identity (name, gender, age, birth order of the child in the family and others), health status, food consumption, serum retinol level, nutritional status (measured by anthropometric index, namely weight-for-age, z-score/WAZ, height), morbidity<br>- data of children’s identity: through interviews<br>- children and their caregivers using questionnaires<br>- health status (morbidity): through observations and interviews<br>food consumption: through food recall method<br>The child’s birth order between the RPO and the control groups was not significantly different (p=0.396). Most of the subjects’ mother (61.3%) took care of their children by themselves while other had the other family members take care their children (38.7%).	
	**Subgroups measure**	not mentioned	
	**Subgroups reported**	not mentioned	

**Interventions**	**Intervention(s)**	RPO (red palm oil) group
	**Comparator(s)**	Control group: non-branded cooking oil
	**Dosage**<br>*(e.g recorded by FFQ)*	-1 kg/week for eight weeks<br>- "Fortified cooking oil given to the subjects contributed 53.4 3- 10.5 RE per day (10,7%) RDA) to meet the requirement of vitamin A. "
	**Fortified oil**<br>*(e.g type, name ect.)*	non-branded cooking oil fortified with carotene from RPO (red palm oil)
	**Duration of intervention**	8 weeks
	**Duration of follow-up**	8 weeks
	**Run-in period**	–
	**Concomitant intervention**	"The average consumption of cooking oil in the RPO group increased because the children liked foods that absorbed a lot of cooking oil such as fish chicken and tempeh."<br>"Increased serum level in the RPO group was caused by the intake of vitamin A and carotene from other food sources as well as from fortified cooking oil."
	**Timing**<br>*(e.g. frequency, duration of each episode)*	oils given to the family once a week, 1 kg/week for eight weeks

		**Description**
**Outcomes**	**Primary outcomes defined by the authors**	-Serum retinol
	**Secondary outcomes defined by the authors**	- Weight to age z-score (WAZ) (results not reported)<br>-BMI<br>-Energy, protein, and fat intake<br>-Dietary vitamin A intake<br>-Consumption of cooking oil<br>-Sub-clinical vitamin A deficiency<br>-IgG level<br>-Morbidity score
	**Confounder** *(uncontrolled, but not adjusted; list of variables authors adjusted variables; Methods used to control to confounders)*	

		**Description**			
**Outcome 1.**	**Outcome name**	WAZ (results not reported)			
	**Outcome definition** *(with diagnostic criteria if relevant)*	–			
	**Time point**<br>*(specify from start or end of intervention) for example 3,6,9,12*	Before-after intervention			
	**Unit of analysis**<br>*(individuals, cluster/ groups or body parts)*	Subjects (2 groups)			
	**Post-intervention or change from baseline?**	Post intervention			
	**Unit of measurement**	%			
	**Results**<br><br>*Nutritional status*<br>*See in Text in Figure 2*	**Intervention group**			
		**Time point**	**Mean**<br>*(if it Median than correct this part please to that)*	**SD**<br>*(or other variance, specify)*	**No. participants**
		Before intervention Thin			13,3%
		Before intervention Normal			86,7%
		After intervention Thin			0%
		After intervention Normal			100%
		**Comparison group**			
		**Time point**	**Mean**<br>*(if it Median than correct this part please to that)*	**SD**<br>*(or other variance, specify)*	**No. participants**
		Before intervention Thin			0%
		Before intervention Normal			100%
		After intervention			0%

		Thin			
		After intervention<br>Normal			100%
	**Any other results reported**<br>*(e.g. mean difference, CI, P value)*				

**Outcome 2.**		**Description**			
	**Outcome name**	Energy, protein and fat intake			
	**Outcome definition** *(with diagnostic criteria if relevant)*	-			
	**Time point**<br>*(specify from start or end of intervention) for example 3,6,9,12*	Before-after intervention<br>0, 8 weeks			
	**Unit of analysis**<br>*(individuals, cluster/ groups or body parts)*	between the two groups			
	**Post-intervention or change from baseline?**	post-intervention			
	**Unit of measurement**	Kcal, g			
	**Results**<br>See in Text in Table 1	**Intervention group RPO group**			
		**Time point**	**Mean**<br>*(if it Median than correct this part please to that)*	**SD**<br>*(or other variance, specify)*	**No. participants**<br>-
		Before intervention			
		Energy (Cal)	1123	398	15
		Protein (g)	27.1	13.4	15
		Fat (g)	36.4	16.5	15
		After intervention			
		Energy (Cal)	1.264	2014	15
		Protein (g)	31.4	7.2	15
		Fat (g)	38.6	7.5	15
		p-value E: 0.243, P: 0.317, Fat: 0.642			
		**Comparison group Control group**			
		**Time point**	**Mean**<br>*(if it Median than correct this part please to that)*	**SD**<br>*(or other variance, specify)*	**No. participants**<br>-
		Before intervention			
		Energy (Cal)	1184	288	16
		Protein (g)	22.9	4.9	16
		Fat (g)	31.7	7.6	16
		After intervention			
		Energy (Cal)	906	198	16
		Protein (g)	19.9	4.2	16
		Fat (g)	24.8	6.9	16
		p-value E: 0.004, P: 0.054, Fat: 0.007			
	**Any other results reported**<br>*(e.g. mean difference, CI, P value)*	-results of paired difference test showed: the average intake of E, protein, fat were not significantly different (p>0.05)<br>-an increase of energy intake in RPO group was caused by an increase in the intake of protein 15,9%, fat 6%, CHO 12,4%<br>-decrease in energy intake of control group after the intervention was result of a decrease in the intake CHO 40.9%, protein 13,1%, fat 21,8%<br>-*See in Text in Table 8* Comparison of Indonesian and Philippines RDA for children aged 7-9 years			

**Outcome 3.**		**Description**
	**Outcome name**	dietary vitamin A intake (in RE=Retinol Equivalent)
	**Outcome definition** *(with diagnostic criteria if relevant)*	–
	**Time point**<br>*(specify from start or end of intervention) for example 3,6,9,12*	Before-after intervention<br>0,8 weeks
	**Unit of analysis**<br>*(individuals, cluster/ groups or body parts)*	between the two groups
	**Post-intervention or change from baseline?**	Post-intervention
	**Unit of measurement**	Retinol Equivalent (RE)
	**Results**	**Intervention group**

	**Time point**	**Mean** *(if it Median than correct this part please to that)*	**SD** *(or other variance, specify)*	**No. participants** -
	Before intervention	302.4	190.5	15
	After intervention	464.2	73.8	15
	p-value 0.009			
	**Comparison group**			
	**Time point**	**Mean** *(if it Median than correct this part please to that)*	**SD** *(or other variance, specify)*	**No. participants** -
	Before intervention	196.7	84.2	16
	After intervention	223.6	109.9	16
	p-value	0.151		
**Any other results reported** *(e.g. mean difference, CI, P value)*	average intake of vitamin A was significantly different (p=0.009)			

**Outcome 4.**		**Description**			
	**Outcome name**	Consumption of cooking oil			
	**Outcome definition** *(with diagnostic criteria if relevant)*	Average consumpition of cooking oil per day.			
	**Time point** *(specify from start or end of intervention) for example 3,6,9,12*	Before-after intervention<br>0,8 weeks			
	**Unit of analysis** *(individuals, cluster/ groups or body parts)*	between the two groups			
	**Post-intervention or change from baseline?** *See in Table 2*	post-intervention			
	**Unit of measurement**	g; RE			
	**Results** "Average consumption of cooking oil per day and the average intake of vitamin A from the cooking oil" *See in Text in Table 2 and Table 3*	**Intervention group**			
		**Time point**	**Mean** *(if it Median than correct this part please to that)*	**SD** *(or other variance, specify)*	**No. participants** -
		Average of cooking oil consumption/day (g)			
		Before intervention	14.4	8.9	15
		After intervention	21.4	4.2	15
		Average intake of vitamin A (RE)	53.4	10.5	15
		**Comparison group**			
		**Time point**	**Mean** *(if it Median than correct this part please to that)*	**SD** *(or other variance, specify)*	**No. participants**
		Average of cooking oil consumption/day (g)			
		Before intervention	14.2	7.8	16
		After intervention	12.9	4.7	16
		Average intake of vitamin A (RE)	0.00	0.00	16
	**Any other results reported** *(e.g. mean difference, CI, P value)*	-RPO oil and control oil consumption was not significantly different before the intervention (p=0.957),<br>-average consumption of oils: RPO 14.4±8.9, control oil 14.2±7.8 g<br>-after the intervention the consumption of cooking oil in RPO group increased to 21.4±4.1, but it decreased slightly to 12.9±4.5 in the control goup,<br>-consumption of cooking oil after the intervention was significantly higher in RPO group than in the control group (p=0000)			

**Outcome 5.**		**Description**
	**Outcome name**	Serum retinol<br>(Serum retinol level and immune response /as measured by serum IgG level/)
	**Outcome definition** *(with diagnostic criteria if relevant)*	
	**Time point** *(specify from start or end of intervention) for example 3,6,9,12*	Before-after intervention<br>0,8 weeks

	**Unit of analysis** *(individuals, cluster/ groups or body parts)*	between the two groups			
	**Post-intervention or change from baseline?**	Post-intervention and change from baseline			
	**Unit of measurement**	µg/dl			
	**Results** "Average of serum retinol level before and after intervention in the RPO and control group" *See in Text in Table 4*	**Intervention group** RPO oil			
		**Time point**	**Mean** *(if it Median than correct this part please to that)*	**SD** *(or other variance, specify)*	**No. participants**
		Before intervention	10.44	1.81	15
		After intervention	15.76	3.12	15
		Delta	5.31	3.29	15
		p-value	0.000		
		**Comparison group** Control oil			
		**Time point**	**Mean** *(if it Median than correct this part please to that)*	**SD** *(or other variance, specify)*	**No. participants**
			l		-
		Before intervention	10.88	2.53	16
		After intervention	14.12	3.41	16
		Delta	3.25	2.64	16
		p-value	0.000		
	**Any other results reported** *(e.g. mean difference, CI, P value)*	–			

**Outcome 6.**		**Description**			
	**Outcome name**	Sub-clinical vitamin A deficiency			
	**Outcome definition** *(with diagnostic criteria if relevant)*	The authors describe the distribution of subject with efficient, marginal and sufficient vitamin A status. However they did not define the categories.			
	**Time point** *(specify from start or end of intervention) for example 3,6,9,12*	Before-after intervention			
	**Unit of analysis** *(individuals, cluster/ groups or body parts)*	between the two groups			
	**Post-intervention or change from baseline?**	Post intervention			
	**Unit of measurement**	%			
	**Results** "Distribution of subjects in the RPO and control groups based on the vitamin A status before and after intervention" *See in Text in Fig 1*	**Intervention group** RPO oil			
		**Time point**	**Mean** *(if it Median than correct this part please to that)*	**SD** *(or other variance, specify)*	**No. participants**
		Before intervention	53.3 deficient	46.7 marginal (with low vitamin A status)	0 sufficient
		After intervention	0 deficient	86.7 marginal	13.3 sufficient
		**Comparison group** Control oil			
		**Time point**	**Mean** *(if it Median than correct this part please to that)*	**SD** *(or other variance, specify)*	**No. participants**
		Control oil	Subjects (%)		-
		Before intervention	37.5 deficient	62.5 marginal	0 sufficient
		After intervention	12.5 deficient	81.2 marginal	6.2 sufficient
	**Any other results reported** *(e.g. mean difference, CI, P value)*	"The avarage of vitamin A deficiency increased in both groups, but only in RPO group that was categorized as sufficient."<br>"There was no subject categorized as sufficient or excessive in both groups."<br>"After intervention for the past eight weeks, there had been improvements in vitamin A status in both groups."			

**Outcome 7.**	**Description**

	**Outcome name**	Body Mass Index (BMI)
	**Outcome definition** *(with diagnostic criteria if relevant)*	
	**Time point** *(specify from start or end of intervention) for example 3,6,9,12*	Before-after intervention<br>0,8 weeks
	**Unit of analysis** *(individuals, cluster/ groups or body parts)*	between the two groups
	**Post-intervention or change from baseline?**	Post-intervention
	**Unit of measurement**	kg/m^2

Results
"Average of Body Mass Index (BMI) before and after intervention in the RPO and control groups"
See in Text in Table 5

Intervention group RPO oil

**Time point**	**Mean** *(if it Median than correct this part please to that)*	**SD** *(or other variance, specify)*	**No. participants**
Before intervention	14.51	0.64	15
After intervention	15.19	0.84	15

Comparison group Control oil

**Time point**	**Mean** *(if it Median than correct this part please to that)*	**SD** *(or other variance, specify)*	**No. participants**
Before intervention	14.18	0.62	16
After intervention	14.50	0.85	16

**Any other results reported** *(e.g. mean difference, CI, P value)*<br>*See Figure 2*<br>*Nutritional status*	"The average of overall BMI before and after intervention was 14.34±0.64 kg/m2 and it increased to 14.84±0.90kg/m2 after the intervention."

		**Description**
**Outcome 8.**	**Outcome name**	Level of immunoglobulin G (IgG)
	**Outcome definition** *(with diagnostic criteria if relevant)*	
	**Time point** *(specify from start or end of intervention) for example 3,6,9,12*	Before-after intervention<br>0,8 weeks
	**Unit of analysis** *(individuals, cluster/ groups or body parts)*	between the two groups
	**Post-intervention or change from baseline?**	Post-intervention and change
	**Unit of measurement**	mg/mL

Results
"Level of IgG before and after intervention in the RPO and control group"
See in Text in Table 6

Intervention group RPO oil

**Time point**	**Mean** *(if it Median than correct this part please to that)*	**SD** *(or other variance, specify)*	**No. participants**
Before intervention	313.73	148.99	15
After intervention	296.34	178.01	15
Delta	-17.39	204.36	

Comparison group Control oil

**Time point**	**Mean** *(if it Median than correct this part please to that)*	**SD** *(or other variance, specify)*	**No. participants**
Before intervention	185.83±179.47	179.47	16
After intervention	279.75±342.11	342.11	16
Delta	93.92±365.03	365.03	

**Any other results reported** *(e.g. mean difference, CI, P value)*	"The data of IgG level in both groups at the end of research were not significantly different (p>0.05)"

		**Description**
**Outcome 9.**	**Outcome name**	Morbidity
	**Outcome definition** *(with diagnostic criteria if relevant)*	"Morbidity is defined as the number of subjects who were sick within 2 weeks prior to intervention and 8 weeks after the intervention".

		"Score of morbidity was the result of frequency of illness multiplied by duration of illness.<br>3 categories: low (<4), medium (4-7), high (>8)
	**Time point** *(specify from start or end of intervention) for example 3,6,9,12*	Before intervention, intermediate 1, intermediate 2, intermediate 3, after intervention<br><br>0,2,4,6,8 weeks
	**Unit of analysis** *(individuals, cluster/ groups or body parts)*	between the two groups
	**Post-intervention or change from baseline?**	Post intervention and change
	**Unit of measurement**	Mean SD, categories (score of morbidity)
	**Results**<br>"Average score of morbidity in the RPO and control groups before and after intervention"<br>*See in Text in Table 7*	**Intervention group** RPO oil<br><br>**Time point** / **Mean** *(if it Median than correct this part please to that)* / **SD** *(or other variance, specify)* / **No. participants**<br>Before intervention / 4.87 / 5.79 / 15<br>After intervention / 0.67 / 2.58 / 15<br><br>**Comparison group** Control oil<br><br>**Time point** / **Mean** *(if it Median than correct this part please to that)* / **SD** *(or other variance, specify)* / **No. participants**<br>Before intervention / 4.44 / 5.10 / 16<br>After intervention / 0.00 / 0.00 / 16
	**Any other results reported** *(e.g. mean difference, CI, P value)*	-Average score of morbidity was classified mild (before intervention) and it was decreased in intermediate 3<br>-Morbidity scores were low in intermediate 1,2,3<br>-"After the intervention, the score in the RPO group increased to 0.67±2.58 because one subject suffered from the mumps in the last two weeks of intervention."

	**Conclusion**
**Conclusion**	"Intervention with non-branched cooking oil fortified with carotene from RPO tended to increase blood retinol level but it did not increase IgG level and morbidity score."

		**Description**
**Study Characteristics**	**Study ID**	#1013
	**Author, date**	**Donglan, 2006**
	**Publication type** *(e.g. full report, abstract, letter)*	full text
	**Language of publication**	Chinese
	**Funding**	The project of "China Oil and Foodstuffs (Group) Co.
	**Conflict** *(notable conflicts of interest of study authors)*	not define
**Methods**	**Aim of study**	To observe the effect of vitamin A (VA) on the improvement of immune function in adolescent children during nutritional interventions with vitamin A (VA) fortified edible oils.
	**Study design** *(RCT/ CCT/ cohort/ controlled before-after/ interrupted time series)*	controlled clinical trail
	**Unit of allocation** *(by individuals, cluster/ groups or body parts)*	individuals
	**Number of study arms**	2
	**Number of study centres**	not mention
	**Start date of study**	not defined
	**End date of study**	not defined
**Participants**	**Population description** *(from which study participants are drawn)*	9-11 years old children with vitamin A deficiency from Beijing, Shenyang, Wubing, and Hefei (China)
	**Country where trial was performed**	China
	**Location/Setting**	Beijing, Shenyang, Wubing, and Hefei (the urban-rural combination of these four cities)
	**Number of study centers**	not defined
	**Inclusion criteria**	9-11 years old school children<br>Vitamin A deficiency (not define the cut off point)
	**Exclusion criteria**	not define

	**Number of participants**	Number of contacted persons	150-200 children
		Agreed to participate	not define
		Sample size *(started the study)*	not define
		Lost to follow-up/withdrawals/drop outs	not define
		Number of participant analysed	174
		Sample size of total group	174
		Sample size of each groups	Intervention group: 87<br>Control group:87
	**Sex** *(Male/Female; Boys/Girls)*	not define	
	**Mean age (age range)**	not define (9-11 years old)	
	**Race/Ethnicity**	not define	
	**Other relevant baseline data** *(e.g. BMI, health status, pregnant ect.)*	not define	
	**Subgroups measure**	-	
	**Subgroups reported**	reported the result by city	
**Interventions**	**Intervention(s)**	VA-enhanced edible vegetable oil	
	**Comparator(s)**	vegetable oil	
	**Dosage** *(e.g recorded by FFQ)*	1 kg of vegetable oil per person	
	**Fortified oil** *(e.g type, name ect.)*	edible vegetable oil (produced by Fulinmen) 7500 hg/kg (GB14880)	
	**Duration of intervention**	5 months	
	**Duration of follow-up**	not mention	
	**Run-in period**	not mention	
	**Concomitant intervention**	not mention<br>(at the end of intervention the children from the control group received the fortified oil).	
	**Timing** *(e.g. frequency, duration of each episode)*	before, after intervention	

		**Description**
**Outcomes**	**Primary outcomes defined by the authors**	Serum vitamin A, Immunoglobulin A, Immunoglobulin B, Immunoglobulin M, Serum Complement C3
	**Secondary outcomes defined by the authors**	
	**Confounder** *(uncontrolled, but not adjusted; list of variables authors adjusted variables; Methods used to control to confounders)*	

		**Description**			
**Outcome 1.**	**Outcome name**	Serum vitamin A			
	**Outcome definition** *(with diagnostic criteria if relevant)*	-			
	**Time point** *(specify from start or end of intervention) for example 3,6,9,12*	0, 5 months			
	**Unit of analysis** *(individuals, cluster/ groups or body parts)*	group			
	**Post-intervention or change from baseline?**	post intervention			
	**Unit of measurement**	µg/l			
	**Results**	**Intervention group**			
		**Time point**	**Mean**	**SD**	**No. participants**
		Baseline	not define		
		After intervention	416.4	123	87
		**Comparison group**			
		**Time point**	**Mean**	**SD**	**No. participants**
		Baseline	not define		
		After intervention	261.2	81.6	87
	**Any other results reported** *(e.g. mean difference, CI, P value)*	The result showed that the serum VA levels of intervention group were significantly higher than control group.			

Outcome 1.		Description			
	**Outcome name**	Immune function indicators (IgA, IgG, IgM)			
	**Outcome definition** *(with diagnostic criteria if relevant)*	Serum immunoglobulins			
	**Time point** *(specify from start or end of intervention) for example 3,6,9,12*	0, 5 months			
	**Unit of analysis** *(individuals, cluster/ groups or body parts)*	group			
	**Post-intervention or change from baseline?**	post intervention			
	**Unit of measurement**	g/l			
	**Results IgA**	**Intervention group**			
		**Time point**	**Mean**	**SD**	**No. participants**
		Baseline	not define		
		After intervention	1.37	0.52	40
		**Comparison group**			
		**Time point**	**Mean**	**SD**	**No. participants**
		Baseline	not define		
		After intervention	1.06	0.55	40
	**Results IgG**	**Intervention group**			
		**Time point**	**Mean**	**SD**	**No. participants**
		Baseline	not define		
		After intervention	8.8	3.27	39
		**Comparison group**			
		**Time point**	**Mean**	**SD**	**No. participants**
		Baseline	not define		
		After intervention	9	3.61	40
	**Results IgM**	**Intervention group**			
		**Time point**	**Mean**	**SD**	**No. participants**
		Baseline	not define		
		After intervention	0.93	0.44	40
		**Comparison group**			
		**Time point**	**Mean**	**SD**	**No. participants**
		Baseline	not define		
		After intervention	0.96	0.42	40
	**Any other results reported** *(e.g. mean difference, CI, P value)*	The average IgA level of the intervention group was higher than control group ($p<0.05$). However, in Hefei and Shenyang, there was no statistical difference between the two groups.<br>The IgG and IgM levels were not significantly different between the control group and the intervention group.			

Outcome 1.		Description			
	**Outcome name**	Serum Complement C3			
	**Outcome definition** *(with diagnostic criteria if relevant)*	-			
	**Time point** *(specify from start or end of intervention) for example 3,6,9,12*	0, 5 months			
	**Unit of analysis** *(individuals, cluster/ groups or body parts)*	group			
	**Post-intervention or change from baseline?**	post intervention			
	**Unit of measurement**	g/l			
	**Results**	**Intervention group**			
		**Time point**	**Mean**	**SD**	**No. participants**
		Baseline	not define		
		After intervention	1.41	0.51	41
		**Comparison group**			

		Time point	Mean	SD	No. participants
		Baseline	not define		
		After intervention	1.07	0.46	38
	**Any other results reported** *(e.g. mean difference, CI, P value)*	Complement C3 was significantly higher in the intervention group.			

	Conclusion
**Conclusion**	The results showed that after 5 months of VA fortification of edible oil, there was a significant increase in the level of serum VA. IgA and C3 levels were significantly higher in the intervention group than those of the control group.

		Description	
**Study Characteristics**	**Study ID**	#600	
	**Author, date**	**Candelaria, 2005**	
	**Publication type** *(e.g. full report, abstract, letter)*	full article in peer-reviewed journal	
	**Language of publication**	English	
	**Funding**	Bureau of Agricultural Research of the Department of Agriculture	
	**Conflict** *(notable conflicts of interest of study authors)*	not mentioned	
**Methods**	**Aim of study**	**"**The study aimed to determine the effect of the consumption of Vitamin A-fortified coconut cooking oil (VAFCCO) on the vitamin A status of the at risk group where vitamin A deficiency is a public health problem. Specifically, the study sought to: determine the contribution of VAFCCO to children's total vitamin A intake, relative to other sources of vitamin A in their current diet; identify factors that influence the children's serum retinol (SR) concentration; and evaluate the effect of VAFCCO intervention on the children's vitamin A status."	
	**Study design** *(RCT/ CCT/ cohort/ controlled before-after/ interrupted time series)*	RCT<br>interventional trial, randomly selected patients	
	**Unit of allocation** *(by individuals, cluster/ groups or body parts)*	"Subjects were randomly allocated"<br>blinded and not blinded part	
	**Number of study arms**	3 (1 experimental, 2 control groups)	
	**Number of study centres**	2 ("In each province, one municipality was selected.", Palauig: six sample villages (Zambales); Lian five sample villages (Batangas)	
	**Start date of study**	September 2000	
	**End date of study**	not mentioned	
**Participants**	**Population description** *(from which study participants are drawn)*	Filipino children aged 4 to 7 years	
	**Country where trial was performed**	Philippines	
	**Location/Setting**	Zambales & Batangas provinces<br><br>„in two provinces in the Philippines, Zambales and Batangas, where vitamin A deficiency was of public health magnitude (>5% deficient with <10µg/dl serum retinol concentration and 15% deficient and low serum retinol with <20 µg/dl serum retinol concentration)."	
	**Inclusion criteria**	4 - <7 years old children in the selected villages whose mothers/caregivers indicated consent to participate	
	**Exclusion criteria**	Children who had measles and/or infections that lasted 7 days or more within a month prior to baseline data collection, and children with certain abnormalities i.e. hydrocephalus case	
	**Number of participants**	Number of contacted persons	-
		Agreed to participate	-
		Sample size *(started the study)*	622
		Lost to follow-up/withdrawals/drop outs	80 dropped-out:<br>Experimental group: 40, Control 1: 16, Control 2: 24<br>*(„due to refusal to be pricked for blood collection, were out-of town/transferred residence or sick during data collection, had lower height for- age and weight-for-age z scores and cooking oil intake, and higher baseline serum retinol concentration and fat intake than those who stayed on as subjects")*
		Number of participant analysed	
		Sample size of total group	542

		Sample size of each groups: Experimental n=268, Control1 n=145, Control 2 n=128
	**Sex**<br>*(Male/Female; Boys/Girls)*	Both<br><u>Male:</u> Experimental gorup (n=268) 56.3 %, Control 1 (n=145) 51.0 %, Control 2 (n=128) 46.5 %<br><u>Female:</u> Experimental gorup (n=268) 43.7 %, Control 1 (n=145) 49.0 %, Control 2 (n=128) 53.5 %
	**Mean age**<br>**(age range)**	Experimental: 6.9± 13.3<br>Control 1: 4.9 ± 0.8<br>Control 2: 5.4 ± 7.4<br>„Children in the Experimental group were older than in Control 1 group"
	**Race/Ethnicity**	not mentioned
	**Other relevant baseline data**<br>*(e.g. BMI, health status, pregnant ect.)*	"Baseline socio-demographic characteristics of the study children's households were, to some extent, different between groups. A larger proportion of households in the Experimental and Control 1 groups had larger household size (35 – 36%) and used open pits for burning rubbish or threw garbage in rivers or other places (63% and 61.8%, respectively) compared to households in Control 2 group (28.9% and 58.8%, respectively). The sources of drinking water were deep well and waterworks for almost all households in Control 2 (96.1%). Whereas dug wells or surface water were more common in both the Experimental (10.1%) and Control 1 (10.3%) groups."
	**Subgroups measure**	–
	**Subgroups reported**	–
**Interventions**	**Intervention(s)**	vitamin A fortified coconut oil (vitamin A content 11.42 - 25.22 Cg/g) along with nutrition education<br>● **Experimental group**: VAFCCO (vitamin A-fortified coconut cooking oil) for 6-months; n=308<br>re-packed cooking oil weekly from 17–18 kg institutional containers into white opaque 200-gram calibrated color-coded plastic bottles
	**Comparator(s)**	unfortified coconut oil along with nutrition education<br>● **Control 1:** using unfortified cooking oil for 6 months, 3 teaspoons per day („this was not the same across periodes during the intervention") n=161<br>● **Control 2**: did not receive cooking oil for 6 months, n=153 („mean of 1.45 ± 0.64 teaspoons a day (P <0.01)")
	**Dosage**<br>*(e.g recorded by FFQ)*	"The mean cooking oil intake amongst the Experimental (using VAFCCO) and Control 1 (using unfortified cooking oil) study children was 3 teaspoons per day."
	**Fortified oil**<br>*(e.g type, name ect.)*	Vitamin A fortified coconut oil (vitamin A content 11.42 - 25.22 Cg/g)
	**Duration of intervention**	6 months
	**Duration of follow-up**	„Follow-up data collection of variables mentioned above was done on the **50th day**, mid-intervention (**about 3rd month**), **120th day** and **after 6 months** of intervention."
	**Run-in period**	-
	**Concomitant intervention**	- "Nutrition education sessions on nutrition and vitamin A, its sources and benefits, the advantages of using cooking oil for the utilization of vitamin A, and the importance of the research undertaking were conducted in a community assembly before the start of the intervention for all three groups."<br>- „In addition, the Nutritionist-Dietitians of the Project checked record-keeping, suggested and encouraged mothers/caregivers of Experimental and Control 1 during the twice-a-month household monitoring visits to: (a) provide the subject child with foods cooked in oil; (b) mix cooking oil in the child's viand/broth or rice; or (c) give cooking oil by other means, such as by adding or replacing sachets of oil found in instant noodle packs with the rationed cooking oil."
	**Timing**<br>*(e.g. frequency, duration of each episode)*	–

		**Description**
**Outcomes**	**Primary outcomes defined by the authors**	1. Weight-for-height z-score<br>2. Proportion of children with low WHZ (Nutritional status as indicated by weight-for-height z-scores)<br>3. Morbidity (number of episoed; mean duration of illness)<br>4. Energy, protein and vitamin C intake<br>5. Dietary vitamin A intake (in ug)<br>6. Distribution of subjects by sources of vitamin A /Nutrient intake (using 24h food recall and a modified 1-month food frequency for intake of vitamin A-rich food/<br>7. Serum retinol level<br>8. Sub-clinical vitamin A deficiency<br>9. Cooking practices and feeding methods<br>10. Cooking use
	**Secondary outcomes defined by the authors**	–

	**Confounder** *(uncontrolled, but not adjusted; list of variables authors adjusted variables; Methods used to control to confounders)*	–

**Outcome 1.**		**Description**			
	**Outcome name**	Weight-for height z-scores			
	**Outcome definition** *(with diagnostic criteria if relevant)*	–			
	**Time point** *(specify from start or end of intervention) for example 3,6,9,12*	0 (baseline), 50th, 90th, 120th, 180th days (endpoint)			
	**Unit of analysis** *(individuals, cluster/ groups or body parts)*	2 groups:<br>- Experimental<br>- Control 1			
	**Post-intervention or change from baseline?**	post intervention			
	**Unit of measurement**	–			
	**Results**	**Intervention group**<br>vitamin A Fortified soy Oil group (FO)			
		**Time point**	**Mean** *(if it Median than correct this part please to that)*	**SD** *(or other variance, specify)*	**No. participants**
		Baseline	-0.69	0.76	263
		Endline	-0.69	0.85	263
		**Comparison group**<br>Non-Fortified soy Oil group (NFO), Control 1			
		**Time point**	**Mean** *(if it Median than correct this part please to that)*	**SD** *(or other variance, specify)*	**No. participants**
		Baseline	-0.67	0.96	145
		Endline	-0.67	0.95	145
	**Any other results reported** *(e.g. mean difference, CI, P value)*				

**Outcome 2.**		**Description**			
	**Outcome name**	Proportion of children with low WHZ			
	**Outcome definition** *(with diagnostic criteria if relevant)*	(Nutritional status as indicated by weight-for-height z-scores ***See in text Table 4***)			
	**Time point** *(specify from start or end of intervention) for example 3,6,9,12*	0 (baseline), 50th, 90th, 120th, 180th days (endpoint)			
	**Unit of analysis** *(individuals, cluster/ groups or body parts)*	**2** groups:<br>- Experimental<br>- Control 1			
	**Post-intervention or change from baseline?**	-			
	**Unit of measurement**	%			
	**Results**	**Intervention group**<br>vitamin A Fortified soy Oil group (FO)			
		**Time point**	**Mean** *(if it Median than correct this part please to that)*	**SD** *(or other variance, specify)*	**No. participants**
		–	–	–	–
		% Low weight-for-height <20 μg/dl			
		Baseline	2.6		263
		Endline	4.1		263
		**Comparison group**<br>Non-Fortified soy Oil group (NFO), Control 1			
		**Time point**	**Mean** *(if it Median than correct this part please to that)*	**SD** *(or other variance, specify)*	**No. participants**
		–	–	–	–
		% Low weight-for-height <20 μg/dl			
		Baseline	3.4		145

		Endline	4.1		145
	**Any other results reported**				

**Outcome 3.**		**Description**			
	**Outcome name**	Morbidity (number of episode; mean duration of illness)			
	**Outcome definition** *(with diagnostic criteria if relevant)*	Morbidity<br><br>„Morbidity data including occurrence, frequency and duration of diarrhea, measles, fever and acute respiratory infections”<br>„Morbidity were monitored from caregivers' daily records using calendar-type monitoring sheets”			
	**Time point** *(specify from start or end of intervention) for example 3,6,9,12*	daily records			
	**Unit of analysis** *(individuals, cluster/ groups or body parts)*	2 groups:<br>- Experimental<br>- Control 1			
	**Post-intervention or change from baseline?**	Post intervention			
	**Unit of measurement**	Daily record, occurrence, frequency and duration of episodes			
	**Results**	**Intervention group**<br>vitamin A Fortified soy Oil group (FO)			
		**Time point**	**Mean** *(if it Median than correct this part please to that)*	**SD** *(or other variance, specify)*	**No. participants**
		–	–	–	–
		**Comparison group**<br>Non-Fortified soy Oil group (NFO)			
		**Time point**	**Mean** *(if it Median than correct this part please to that)*	**SD** *(or other variance, specify)*	**No. participants**
		–	–	–	–
	**Any other results reported** *(e.g. mean difference, CI, P value)*	***Other important notes:***<br>„During the intervention, there were no significant differences between study groups in the prevalence,frequency and duration of illnesses at any period.”			

**Outcome 4.**		**Description**			
	**Outcome name**	Energy, protein, and vitamin C intake			
	**Outcome definition** *(with diagnostic criteria if relevant)*	–,<br>(Nutrient intake (using 24h food recall), („Mother/caregivers were interviewed on study children's food intake using 24-hour food recall and a modified 1-month food frequency for intake of vitamin A-rich food, receipt of high-dose VAC and other multivitamins, and occurrence of infection for the immediate past month.”)			
	**Time point** *(specify from start or end of intervention) for example 3,6,9,12*	0 (baseline), 50th, 90th, 120th, 180th days (endpoint)			
	**Unit of analysis** *(individuals, cluster/ groups or body parts)*	2 groups:<br>- Experimental<br>- Control 1			
	**Post-intervention or change from baseline?**	Post intervention			
	**Unit of measurement**	Kcal			
	**Results**	**Intervention group**<br>vitamin A Fortified soy Oil group (FO)			
		**Time point**	**Mean** *(if it Median than correct this part please to that)*	**SD** *(or other variance, specify)*	**No. participants**
		–	–	–	–
		**Comparison group**<br>Non-Fortified soy Oil group (NFO), Control 1			
		**Time point**	**Mean** *(if it Median than correct this part please to that)*	**SD** *(or other variance, specify)*	**No. participants**
		–	–	–	–
	**Any other results reported** *(e.g. mean difference, CI, P value)*	***Other important notes:***			

		Mean one-day **energy intake** ranged from 1058 –1132 kcal (66.1% - 70.8% adequacy).<br>Mean one-day **protein intake** was 30.6g – 36.0g (95.6% - 112.5% adequacy) while **intake of vitamin C** was 20.5mg - 38.2mg (45.6% - 84.9% adequacy).<br><br>"… at all intervention periods, no differences in mean energy, protein and vitamin C intakes and percentage adequacy were noted between study groups, nor were there differences between periods from baseline for all groups."

		**Description**
**Outcome 5.**	**Outcome name**	Dietary vitamin A intake (in ug)
	**Outcome definition** *(with diagnostic criteria if relevant)*	modified 1-month food-frequency questionnaire, baseline mean vitamin A intake"
	**Time point** *(specify from start or end of intervention) for example 3,6,9,12*	0 (baseline), 50th, 90th, 120th, 180th days (endpoint)
	**Unit of analysis** *(individuals, cluster/ groups or body parts)*	**2** groups:<br>- Experimental<br>- Control 1
	**Post-intervention or change from baseline?**	Post intervention
	**Unit of measurement**	µg per day and %
	**Results**<br>**See in text Table 3**<br>Mean vitamin A intake (µg) per day and % distribution of subjects by source of vitamin A by study group by period	**Intervention group**<br>vitamin A Fortified soy Oil group (FO)

Time point	Mean *(if it Median than correct this part please to that)*	SD *(or other variance, specify)*	No. participants
Baseline	789.0	540.9	268
3rd Period (121st - post)	936.8	719.9	268

Comparison group
Non-Fortified soy Oil group (NFO)

Time point	Mean *(if it Median than correct this part please to that)*	SD *(or other variance, specify)*	No. participants
Baseline	646.8	±657.2	144
3rd Period (121st - post)	952.5	±161.2	144

	**Any other results reported** *(e.g. mean difference, CI, P value)*	Relationships between variables:<br>● Vitamin A intake by different sources<br>● VAFCCO intake and nutritional status as indicated by weight-for-height z-scores<br>● Vitamin A intake, vitamin A status and morbidity<br>● Determinants of vitamin A status<br>**Vitamin A intake by different sources:** "Percent contribution of vitamin A from fortified foods other than cooking oil and from other vitamin A-rich foods were positively correlated at all periods (r = 0.217 – 0.517). The analysis showed higher percentage vitamin A and vitamin A-rich foods (r = 0.181) with increasing purchase of cooking oil."<br>Vitamin A sources: vitamin A sources (Sangkap Pinoy Seal products, Vitamin A-rich foods, supplements)<br>**VAFCCO intake and nutritional status as indicated by weight-for-height z-scores**: "Subjects who consumed high amounts of VAFCCO (>15g per day) improved in weight-for-height z-scores by 80%. The weight-for-height z-scores decreased by >80% for those who consumed average amount of VAFCCO (5 to 15g per day) from base to after 6 months of cooking oil intervention. The findings show that there was no significant effect of VAFCCO intake on nutritional status of subjects.", "There was no significant association between nutritional status and percent vitamin A contribution from cooking oil, even as children with lower height-for-age (r = -0.132) or weight-for age z scores (r = -0.114) tend to have higher vitamin A intake from cooking oil. Serum retinol concentration was significantly associated with lower cooking oil intake (r = -0.121), but not with the intake of VAFCCO except at the end of the intervention period (r = -0.122)."<br>**Vitamin A intake, vitamin A status and morbidity**: no relationship<br>**Determinants of vitamin A status**: „Consumption of VAFCCO alone was not a determinant of serum retinol concentration."<br>"The significant determinants of serum retinol concentration post-intervention, after considering variables on household and socio-demographic characteristics, nutritional status and morbidity, and cooking oil (both VAFCCO and unfortified cooking oil) and vitamin A intake were as follows (step-wise): serum retinol concentration at baseline (β = 0.282), interaction of buying cooking oil and food expenditure (β = 0.004), education of caregiver (β = 0.109), receipt of high-dose

		vitamin A capsule (β = 1.214), and interaction of vitamin A from vitamin A-rich foods and cooking oil (β = 0.000005)."

Outcome 6.

	Description
**Outcome name**	Distribution of subjects by sources of vitamin A
**Outcome definition** *(with diagnostic criteria if relevant)*	„modified 1-month food-frequency questionnaire, baseline mean vitamin A intake"
**Time point** *(specify from start or end of intervention) for example 3,6,9,12*	0 (baseline), 50th, 90th, 120th, 180th days (endpoint)
**Unit of analysis** *(individuals, cluster/ groups or body parts)*	**2** groups:<br>- Experimental<br>- Control 1
**Post-intervention or change from baseline?**	–
**Unit of measurement**	%

Results
See in text Table 3
Vitamin A intake and sources of vitamin A

Intervention group
vitamin A Fortified soy Oil group (FO)

**Time point**	**Mean** *(if it Median than correct this part please to that)*	**SD** *(or other variance, specify)*	**No. participants**
-	-	-	-
**Period and sources** *Baseline*			
% cooking oil		29.0	
% other fortified foods		31.5	
% other vitamin A rich food		39.2	
**Period and sources** *3rd Period (121st - post)*			
% cooking oil		26.5	
% other fortified foods		25.2	
% other vitamin A rich food		48.4*	

Comparison group
Non-Fortified soy Oil group (NFO)

**Time point**	**Mean** *(if it Median than correct this part please to that)*	**SD** *(or other variance, specify)*	**No. participants**
-	-	-	-
**Period and sources** *Baseline*			
% cooking oil	4.3		
% other fortified foods	42.8		
% other vitamin A rich food	52.3		
**Period and sources** *3rd Period (121st - post)*			
% cooking oil	0*		
% other fortified foods	35.2		
% other vitamin A rich food	64.8*		

**Any other results reported** *(e.g. mean difference, CI, P value)*	**Vitamin A sources:** vitamin A sources (Sangkap Pinoy Seal products, Vitamin A-rich foods, supplements)<br>**Vitamin A intake by different sources:** "Percent contribution of vitamin A from fortified foods other than cooking oil and from other vitamin A-rich foods were positively correlated at all periods (r = 0.217 – 0.517). The analysis showed higher percentage vitamin A and vitamin A-rich foods (r = 0.181) with increasing purchase of cooking oil."<br>"The significant determinants of serum retinol concentration post-intervention, after considering variables on household and socio-demographic characteristics, nutritional status and morbidity, and cooking oil (both VAFCCO and unfortified cooking oil) and vitamin A intake were as follows (step-wise): serum retinol concentration at baseline (β = 0.282), interaction of buying cooking oil and food expenditure (β = 0.004), education of caregiver (β = 0.109), receipt of high-dose vitamin A capsule (β = 1.214), and interaction of vitamin A from vitamin A-rich foods and cooking oil (β = 0.000005)."<br>"The constant contact with the nutritionist-dietitian researchers may have motivated the mothers/caregivers to improve the subjects' food intake and to become more aware of the sources of vitamin A."

Outcome 7.

	Description
**Outcome name**	Serum retinol level

	**Outcome definition** *(with diagnostic criteria if relevant)*	–
	**Time point** *(specify from start or end of intervention) for example 3,6,9,12*	0 months (baseline), 6 months
	**Unit of analysis** *(individuals, cluster/ groups or body parts)*	2 groups:<br>- Experimental<br>- Control 1
	**Post-intervention or change from baseline?**	Post-intervention and change from baseline
	**Unit of measurement**	μg/dL; %;<br>low, marginal, acceptable, high
	**Results**<br>**See in Text Table 5**<br><br>**See in Text Figure 2.** Percent distribution of subjects by serum retinol concentration by study group at baseline and after 6 months of intervention<br><br>**See in Text Fig 3.**<br>Percent of subjects with increased serum retinol concentration by study group by study period	**Intervention group**<br>vitamin A Fortified soy Oil group (FO)

Time point	Mean *(if it Median than correct this part please to that)*	SD *(or other variance, specify)*	No. participants
**Se retinol**			
Baseline (μg/dL±SD)	28.81	8	268
After 6 months (μg/dL±SD)	36.88	7	268
Relative change (%)		36.1	
**Se retinol concentration**, WHO cut-offs	Baseline	After 6 months	
Low	11.6	no data	
Marginal	18.3	2.6	
Acceptable	69.4	93.7	
**Se retinol concentration** (Figure3)			
	% of subjects with improvement in VAS		
50th day	22.4		
120th day	26.5		
180th day	33.2		

Comparison group
Non-Fortified soy Oil group (NFO)

Time point	Mean *(if it Median than correct this part please to that)*	SD *(or other variance, specify)*	No. participants
**Se retinol**			
Baseline (μg/dL±SD)	30.77	±9	145
After 6 months (μg/dL±SD)	37.94	±8[3]	145
Relative change (%)		31.5*	
**Se retinol concentration**, WHO cut-offs	Baseline	After 6 months	
Low	7.6	no data	
Marginal	15.9	1.4	
Acceptable	72.4	89.6	
**Se retinol concentration** (Figure3)			
	% of subjects with improvement in VAS		
50th day	16.6		
120th day	18		
180th day	33.8		

**Any other results reported** *(e.g. mean difference, CI, P value)*	"Among children in the Control 1 group who had low baseline serum retinol "concentration, 91% improved to acceptable concentration while 9% had marginal concentration at end of the study."<br><br>"Among study children who had marginal baseline serum retinol concentration, 98.1% in the Experimental group improved vis-àvis 95.6% in the Control 1 group and 100% in the Control2."

**Outcome 8.**		**Description**
	**Outcome name**	Sub clinical vitamin A deficiency
	**Outcome definition** *(with diagnostic criteria if relevant)*	–

	**Time point** *(specify from start or end of intervention) for example 3,6,9,12*	0 months (baseline), 6 months
	**Unit of analysis** *(individuals, cluster/ groups or body parts)*	2 groups:<br>- Experimental<br>- Control 1
	**Post-intervention or change from baseline?**	Post-intervention and change from baseline
	**Unit of measurement**	%;
	**Results**<br><br>**See in Text Figure 2.** Percent distribution of subjects by serum retinol concentration by study group at baseline and after 6 months of intervention	**Intervention group**<br>vitamin A Fortified soy Oil group (FO)
	**Any other results reported** *(e.g. mean difference, CI, P value)*	„The results of this study revealed that usage of VAFCCO alone is not sufficient to increase vitamin A status of 4 to <7 y old children. However, together with vitamin A-rich foods, intake of VAFCCO showed significant change in the serum retinol level concentration considering the results of multivariate regression. Increased availability and consumption of vitamin A-rich foods to adequate amounts through continued efforts in backyard and community gardening, is an important component of the solution to the problem of vitamin A deficiency."

Intervention group – vitamin A Fortified soy Oil group (FO):

Time point	Mean *(if it Median than correct this part please to that)*	SD *(or other variance, specify)*	No. participants
–	–	–	–
**Se retinol concentration**, WHO cut-offs	Baseline	After 6 months	
High	0.7	3.7	

Comparison group
Non-Fortified soy Oil group (NFO)

Time point	Mean *(if it Median than correct this part please to that)*	SD *(or other variance, specify)*	No. participants
–	–	–	–
**Se retinol concentration**, WHO cut-offs	Baseline	After 6 months	
High	4.1	11.7	

**Outcome 9**.		**Description**
	**Outcome name**	Cooking use
	**Outcome definition** *(with diagnostic criteria if relevant)*	-
	**Time point** *(specify from start or end of intervention) for example 3,6,9,12*	• Baseline<br>• After 6 months
	**Unit of analysis** *(individuals, cluster/ groups or body parts)*	2 groups:<br>- Experimental<br>- Control 1
	**Post-intervention or change from baseline?**	-
	**Unit of measurement**	[1]$P < 0.05$ $\chi 2$ test (within period between groups);
	**Results**<br>**See in Table 2**	**Intervention group**<br>vitamin A Fortified soy Oil group (FO)

Intervention group – vitamin A Fortified soy Oil group (FO):

Time point	Mean *(if it Median than correct this part please to that)*	SD *(or other variance, specify)*	No. participants
**Use of cooking oil in a day**[1]	Baseline	After 6 months	
1 – 2	75.0	68.2	n = 268
> 3	25.0**	31.8**	n = 268
**Save used cooking oil for re-use**	64.0*	60.1	n = 268

Comparison group
Non-Fortified soy Oil group (NFO)

Time point	Mean *(if it Median than correct this part please to that)*	SD *(or other variance, specify)*	No. participants
**Use of cooking oil in a day**[1]	Baseline	After 6 months	

1 – 2	64.9**	67.2**	n = 145
> 3	35.1	32.8	n = 145
**Save used cooking oil for re-use**	53.8*	52.4	n = 145

**Any other results reported** *(e.g. mean difference, CI, P value)*	The frequency of use and re-use of cooking oil by households however differed between groups as shown in Table 2.”<br>A larger proportion of households in the experimental group (64.0%) vis-à-vis the Control groups (50.8% – 53.8%) tended to re-use cooking oil. According to Marero et al.,3 re-use of cooking oil lead to 50% loss of vitamin A in its second use.<br><br>„Considering all types of cooking oil used by the study children, the proportion of vitamin A obtained<br>from cooking oil was highest in the Experimental group (29.0%) compared with the two Control groups (4.3% – 4.6%). On the other hand, vitamin A from other fortified foods and other vitamin A rich foods, was higher in both Control groups than in the Experimental group.”<br><br>„The percentage contribution of cooking oil to total vitamin A intake was not different between baseline and the intervention periods in the Experimental group, but had a significant decline for the two Control groups.”<br><br>„The mean cooking oil intake amongst the Experimental<br>(using VAFCCO) and Control 1 (using unfortified<br>cooking oil) study children was 3 teaspoons per day. This was not the same across periods during the intervention (P <0.01), which fluctuated between the 1st, 2nd and 3rd periods in all study groups.”

Outcome 10.

	**Description**
**Outcome name**	Cooking practices
**Outcome definition** *(with diagnostic criteria if relevant)*	–, Cooking practices and feeding methods
**Time point** *(specify from start or end of intervention) for example 3,6,9,12*	0 months (baseline)<br>6 months
**Unit of analysis** *(individuals, cluster/ groups or body parts)*	2 groups:<br>- Experimental<br>- Control 1
**Post-intervention or change from baseline?**	Post-intervention
**Unit of measurement**	%

Results
See in text Table 2
Cooking practices and cooking oil use of household subjects (in %) by study group at baseline and after 6 months of intervention

Intervention group
vitamin A Fortified soy Oil group (FO)

**Time point**	**Mean** *(if it Median than correct this part please to that)*	**SD** *(or other variance, specify)*	**No. participants**
–	–	–	–

	**Cooking practice**, n = 268	
	Baseline	After 6 months
Boiled	38.1	41.7
Fried	51.5	82.6
Sautéed	29.9	49.6
Broiled	2.6	6.4

	**Portion size of vegetables fed to child only Baseline data in % n = 268**
cut in big slices	16.2
cut in small slices	71.9**
mashed	11.9

Comparison group
Non-Fortified soy Oil group (NFO)

**Time point**	**Mean** *(if it Median than correct this part please to that)*	**SD** *(or other variance, specify)*	**No. participants**
–	–	–	–

	**Cooking practice**, n = 145	
	Baseline	After 6 months
Boiled	34.5	49.0
Fried	51.7**	85.5**
Sautéed	28.3**	52.4**
Broiled	3.4	6.9

Portion size of vegetables fed to child only Baseline data in %

	Any other results reported *(e.g. mean difference, CI, P value)*	**n = 145**	
		cut in big slices	24.5
		cut in small slices	62.3**
		mashed	13.2
		„At baseline, the groups were the same with regard to cooking methods and the brand of cooking oil commonly used.” The practice of cooking and feeding vegetables to children in smaller cuts tends to enhance utilization of vitamin A in the body. Significant differences in cooking practices were noted across periods in all study groups. The use of frying and sautéing, and cooking oil with vitamin A, increased in all groups during the intervention (P < 0.01). These changes were most likely influenced by the households' interaction with the researchers, including the provision of nutrition education.	

	**Conclusion**
**Conclusion**	„The study, conducted in a community-setting for a period of six-months, has the following conclusions:<br>• VAFCCO intake, which contributed to about 30% of vitamin A intake, was significantly higher among the Experimental group than in the two Control groups, but taken alone was not shown to improve the serum retinol concentration of children 4 to < 7 y old;<br>• the use and consumption of VAFCCO together with intake of vitamin A from other sources such as green leafy and yellow vegetables, liver, fish, meat and eggs, improves serum retinol concentration;<br>• as the intake of fat, whether fortified or not, enhances absorption of vitamin A, the use of coconut oil improves vitamin A status and nutritional status, in general.

		**Description**
**Study Characteristics**	**Study ID**	#293
	**Author, date**	**Atalhi, 2020** (2019, abstract)
	**Publication type** *(e.g. full report, abstract, letter)*	full article in peer-reviewed journals, conference abstract<br>Atalhi 2019: Trial identifier: PACTR201512001217212
	**Language of publication**	English
	**Funding**	“The study was supported by the IAEA and the logistical assistance was provided by CNESTEN (Centre National d'Energie, des Sciences et des Techniques Nucléaires); The study materials (vitamin A fortified and non- fortified oil) were provided by Aïcha Company, Meknes, Morocco, Vitamin A supplements were provided by the Moroccan Ministry of Health.”
	**Conflict** *(notable conflicts of interest of study authors)*	None
**Methods**	**Aim of study**	**“**The purpose of the study is to evaluate the effect of post-partum high dose VA supplementation and provision of VA fortified oil for household consumption on plasma and milk retinol concentrations of lactating Moroccan women during the first 6 mo post-partum.” VA= vitamin A
	**Study design** *(RCT/ CCT/ cohort/ controlled before-after/ interrupted time series)*	longitudinal randomized control trial
	**Unit of allocation** *(by individuals, cluster/ groups or body parts)*	−individuals<br>−unit of randomization: „simple randomization procedure”, blinding: „The fortified and non-fortified oils were pre-packed in coded bottles and were identical in appearance and taste”.
	**Number of study arms**	2
	**Number of study centres**	1
	**Start date of study**	February 2009
	**End date of study**	July 2010
**Participants**	**Population description** *(from which study participants are drawn)*	- mother infant pairs, recruited at 2-3 weeks post-partum<br>- from a local maternity hospital in a low-income community in Rabat<br>- at the time of BCG (Bacille Calmette-Guerin) vaccination of infants<br>- Mothers who breastfeeding a healthy single infant (and planned the breastfeeding) for at least 6 mo postpartum
	**Country where trial was performed**	Morocco ('mentioned low-income countrí in the study')
	**Location/Setting**	Rabat
	**Inclusion criteria**	- mother infant pairs, recruited at 2-3 weeks post-partum<br>- from a local maternity hospital in a low-income community in Rabat<br>- at the time of BCG vaccination of infants<br>- Mothers who breastfeeding a healthy single infant (and planned the breastfeeding) for at least 6 mo postpartum<br>-Written informed consent
	**Exclusion criteria**	-aged <18 y<br>-if they had acute infections/chronic diseases or severe vitamin A deficiency<br>-Women who became pregnant during the study were excluded

		Not meeting inclusion criteria (n=6) · Declined to participate (n=4) · Other reasons (n= 0)	
	**Number of participants**	Number of contacted persons	150
		Agreed to participate	Randomized n=140
		Sample size<br>*(started the study)*	140
		Lost to follow-up/withdrawals/drop outs	Fortified oil, FO group: lost to follow-up n=5; discontinued intervention n=3<br>Non-fortified oil, NFO group: lost to follow-up n=10; discontinued intervention n=21<br><br>- FO Lost to follow-up (n=5) (unauthorized absence, husband refusal, illness, travel) Discontinued intervention (n=3) Reasons: travel, work, family problems<br>- NFO Lost to follow-up (n= 10) (unauthorized absence, husband refusal, illness, travel) Discontinued intervention (n=21) Reasons: travel, work, family problems
		Number of participant analysed	108
		Sample size of total group	108
		Sample size of each groups	NFO= 39, FO=69
	**Sex**<br>*(Male/Female; Boys/Girls)*	Female	
	**Mean age (age range)**	not mentioned (19-40 y)	
	**Race/Ethnicity**	not mentioned	
	**Other relevant baseline data**<br>*(e.g. BMI, health status, pregnant ect.)*		
	**Subgroups measure**	–	
	**Subgroups reported**	–	
**Interventions**	**Intervention(s)**	● FO=Vitamin A fortified soy oil group (n=62)<br><br>FO: 30 IU/g of oil was produced and obtained from Aicha, a local food company in Meknes, Morocco. Soy oil, Delivered weekly (2 liters / week/family amount)	
	**Comparator(s)**	● NFO =Non- Fortified soy Oil group (n=39)	
	**Dosage**<br>*(e.g recorded by FFQ)*	-receive weekly a quantity of 2 litres of vitamin A-fortified Soya oil (HSF) (33 UI.g of oil) or non- fortified Soya oil (HS) (*from Atalhi abstract*); HSF=FO, HS=NFO<br>-2 liters/week: This fortification rate covers one-third of the daily needs of an adult male (Ministere de la Sante 2002). Any leftover oil at the end of the week was weighed and recorded.<br>*(from Atalhi, 2020)*<br>-All women received a capsule containing 200.000 IU (60 RE) *(from Athali, 2020)* of vitamin A at 15 days postpartum.	
	**Fortified oil**<br>*(e.g type, name ect.)*	Vitamin A fortified soy oil (FO)	
	**Duration of intervention**	6 months	
	**Duration of follow-up**	6 months	
	**Run-in period**	–	
	**Concomitant intervention**	„Women were requested to maintain their dietary habits including oil consumption" „Usual consumption of vitamin A-containing foods was assessed weekly by administering a food frequency questionnaire to mothers."<br>-All women received a capsule containing 200.000 IU (60 RE) *(from Athali, 2020)* of vitamin A at 15 days postpartum.	
	**Timing**<br>*(e.g. frequency, duration of each episode)*	–	

		**Description**
**Outcomes**	**Primary outcomes defined by the authors**	–Retinol in breast milk<br>–Proportion of mothers with low concentrations of retinol in their breast milk<br>*(„Mothers' milk retinol (Low mother's milk retinol concentration, Maternal milk retinol concentration": Abstract from Atalhi)*
	**Secondary outcomes defined by the authors**	• Serum retinol concentration<br>• CRP<br>• Breast milk fat<br>• Morbidity (results not reported)

		• Proportion of mothers who consumed foods rich in vitamin A
	**Confounder** *(uncontrolled, but not adjusted; list of variables authors adjusted variables; Methods used to control to confounders)*	not mentioned

		**Description**			
**Outcome 1.**	**Outcome name**	Retinol in breast milk<br>(Mothers' milk retinol)			
	**Outcome definition** *(with diagnostic criteria if relevant)*	"The vitamin A content of milk was expressed as concentration per volume (μmol/L) and per gram of milk fat (μmol/g). According to WHO criteria, values ≤1.05 μmol/L and ≤28 μmol /g of fat were considered low (World Health Organization 1996)."			
	**Time point** *(specify from start or end of intervention) for example 3,6,9,12*	Vitamin A supplementation: 0 months (T0)<br>Human milk sampling: 0 months, 1 months, 2 months, 3 months, 4 months, 5 months, 6 months (T0, T1, T2, T3, T4, T5, T6)<br>weekly administration of oil			
	**Unit of analysis** *(individuals, cluster/ groups or body parts)*	FO group- NFO group			
	**Post-intervention or change from baseline?**	post-intervention			
	**Unit of measurement**	Means (mean ± Standard deviation or median (interquartiles at 25% and 75%) and proportions) or medians nmol/g;<br>% ≤28nmol/g			
	**Results**	**Intervention group** FO group			
		**Time point**	**Mean** *(if it Median than correct this part please to that)*	**SD** *(or other variance, specify)*	**No. participants**
		Maternal milk retinol concentrations, Milk retinol μmol at **Baseline**	1.89	0.47	62
		Maternal milk retinol concentrations, Milk retinol μmol at **6 months**	1.39	0.18	62
		**Comparison group** NFO group			
		**Time point**	**Mean** *(if it Median than correct this part please to that)*	**SD** *(or other variance, specify)*	**No. participants**
		Maternal milk retinol concentrations, Milk retinol μmol at **Baseline**	1.77	0.39	39
		Maternal milk retinol concentrations, Milk retinol μmol at **6 months**	0.60	0.11	39
	**Any other results reported** *(e.g. mean difference, CI, P value)*	„Retinol concentration: By 1 mo post-partum, milk retinol concentration was higher in the FO group than in the NFO group and continued being higher throughout the study period (P<0.0001)."<br><br>*Method: Milk retinol concentration was determined by HPLC (from Atalhii, 2020)*			

		**Description**
**Outcome 2.**	**Outcome name**	Proportion of mothers with low concentrations of retinol in their breast milk
	**Outcome definition** *(with diagnostic criteria if relevant)*	"The vitamin A content of milk was expressed as concentration per volume (μmol/L) and per gram of milk fat (μmol/g). According to WHO criteria, values ≤1.05 μmol/L and ≤28 μmol /g of fat were considered low (World Health Organization 1996)."
	**Time point** *(specify from start or end of intervention) for example 3,6,9,12*	Mother's height and weight measurement, 0 months (T0)<br>Vitamin A supplementation: 0 months (T0)<br>Human milk sampling: 0 months, 1 months, 2 months, 3 months, 4 months, 5 months, 6 months (T0, T1, T2, T3, T4, T5, T6)<br>weekly administration of oil

	**Unit of analysis** *(individuals, cluster/ groups or body parts)*	FO group- NFO group			
	**Post-intervention or change from baseline?**	post-intervention			
	**Unit of measurement**	% *(Results presented as proportions)*			
		**Intervention group** FO group			
		**Time point**	**Mean** *(if it Median than correct this part please to that)*	**SD** *(or other variance, specify)*	**No. participants**
		–	–	–	–
		**Low mother's milk retinol concentrations, <1.05 µmol/l**			
		Low mother's milk retinol concentrations, **baseline** <1.05 µmol/l	5.41		62 (FO group total n)
		Low mother's milk retinol concentrations, **6 months** <1.05 µmol/l	2.7		62 (FO group total n)
		**Comparison group** NFO group			
	**Results** Low mother's milk retinol concentrations at baseline and during 6 mo follow-up in the FO and NFO groups. **See in Table 3**	**Time point**	**Mean** *(if it Median than correct this part please to that)*	**SD** *(or other variance, specify)*	**No. participants**
		–	–	–	–
		**Low mother's milk retinol concentrations, <1.05 µmol/l**			
		Low mother's milk retinol concentrations, **baseline** <1.05 µmol/l	6.5		39 (NFO group total n)
		Low mother's milk retinol concentrations, **6 months** <1.05 µmol/l	100		39 (NFO group total n)
	**Any other results reported** *(e.g. mean difference, CI, P value)*	„Retinol concentration: By 1 mo post-partum, milk retinol concentration was higher in the FO group than in the NFO group and continued being higher throughout the study period (P<0.0001)."<br><br>Mother's characteristics: age, total pregnancies, Education level, Mother's anthropometry (Seca)<br>baby's length and weight were measured (Seca)<br>socio-economic status, including education, number of children, marital status, and working situation, was obtained from all study participants by an interview using an appropriate questionnaire *(from Atalhi, 2020)*			

**Outcome 3.**		**Description**
	**Outcome name**	Serum retinol concentration
	**Outcome definition** *(with diagnostic criteria if relevant)*	
	**Time point** *(specify from start or end of intervention) for example 3,6,9,12*	0 months, 3 months, 6 months (T0, T3, T6)
	**Unit of analysis** *(individuals, cluster/ groups or body parts)*	FO group- NFO group
	**Post-intervention or change from baseline?**	post-intervention
	**Unit of measurement**	µmol/L

	Results	**Intervention group** FO group			
		**Time point**	**Mean** *(if it Median than correct this part please to that)*	**SD** *(or other variance, specify)*	**No. participants**
		**Baseline**	1.49	0.66	62
		**6 months**	3.53	0.57	62
		**Comparison group** NFO group			
		**Time point**	**Mean** *(if it Median than correct this part please to that)*	**SD** *(or other variance, specify)*	**No. participants**
		**Baseline**	1.49	0.66	39
		**6 months**	2.78	0.60	39
	**Any other results reported** *(e.g. mean difference, CI, P value)*	At 3 mo of intervention, mean serum retinol was 2.80 ± 0.69 umol/L and 2.85±0.49 µmol/L for NFO and FO groups respectively (p=0.76).<br>“Our results showed that serum retinol continued to increase beyond the third month in the FO group compared to NFO group; this could be explained by the combined effect of supplementation and fortification.”<br>„Serum retinol concentration was higher in the FO group than in the NFO group at 6 mo postpartum (P<0.0001).”<br>„The analysis of reference materials showed a loss of 15% during the extractions. This loss was taken into account in the calculations of the different concentration of serum retinol.”<br>“We did not expect very high concentrations of serum vitamin A in our population as the recruited mothers were from a low socioeconomic community and therefore we did not measure retinyl esters and retinoic acids to evaluate whether the women had excessive vitamin A status.”			

**Outcome 4.**		**Description**			
	**Outcome name**	CRP			
	**Outcome definition** *(with diagnostic criteria if relevant)*	cut-off of >10 mg/L used for abnormal CRP concentration (def. based on 2 studies)			
	**Time point** *(specify from start or end of intervention) for example 3,6,9,12*	−Vitamin A supplementation: 0 months /T0/<br>−Blood sampling: 0 months, 3 months, 6 months /T0, T3, T6/			
	**Unit of analysis** *(individuals, cluster/ groups or body parts)*	FO group- NFO group			
	**Post-intervention or change from baseline?**	Post-intervention			
	**Unit of measurement**	%			
	Results	**Intervention group** FO group			
		**Time point**	**Mean** *(if it Median than correct this part please to that)*	**SD** *(or other variance, specify)*	**No. participants**
		–	–	–	–
		**Comparison group** NFO group			
		**Time point**	**Mean** *(if it Median than correct this part please to that)*	**SD** *(or other variance, specify)*	**No. participants**
		–	–	–	–
	**Any other results reported** *(e.g. mean difference, CI, P value)*	“The overall prevalence of elevated serum **CRP** was 32.1%, 13.2% and 19.6% at baseline, 3 and 6 months, respectively, and did not differ by group.”<br><br>Method: *CRP was measured by radial immunodiffusion using commercial kits (from Atalhii, 2020)*			

**Outcome 5.**		**Description**
	**Outcome name**	Breast milk fat
	**Outcome definition** *(with diagnostic criteria if relevant)*	*WHO criteria, values ≤1.05 µmol/L and ≤28 µmol /g of fat were considered low (World Health Organization 1996)*
	**Time point** *(specify from start or end of intervention) for example 3,6,9,12*	0.5 months<br>/1months, 2 months, 3 months, 4 months,5 months/<br>6 months

	**Unit of analysis**<br>*(individuals, cluster/ groups or body parts)*	FO group- NFO group			
	**Post-intervention or change from baseline?**	post intervention<br><br>„Milk retinol per g fat at baseline did not differ by group; by 3 mo postpartum, milk retinol per g fat was higher in the FO group than in the NFO group p=0.02) and remained higher throughout 6 mo (P<0.0001).”			
	**Unit of measurement**	per gram of milk fat (µmol/g)			
	**Results**<br>**See in Text in Table 4**<br>Mothers’ milk retinol per gram of fat at baseline and during 6 mo follow-up in the FO and NFO groups	**Intervention group** FO group			
		**Time point**	**Mean**<br>*(if it Median than correct this part please to that)*	**SD**<br>*(or other variance, specify)*	**No. participants**
		Means or medians nmol/g			
		0.5 months	70.2	±50.3	n=62
		6 months	56.7	±38.6, 125.7	n=62
		**% ≤28nmol/g**			
		0.5 months		8.0	
		6 months		8.1	
		**Comparison group** NFO group			
		**Time point**	**Mean**<br>*(if it Median than correct this part please to that)*	**SD**<br>*(or other variance, specify)*	**No. participants**
		Means or medians nmol/g			
		0.5 months	66.4	±42.4	n=39
		6 months	17.4	±14.8, 26.1	n=39
		**% ≤28nmol/g**			
		0.5 months		15.4	
		6 months		79.2	
	**Any other results reported**<br>*(e.g. mean difference, CI, P value)*	At 4, 5 and 6 months post-partum, the milk retinol concentration, expressed per gram milk fat was significantly higher in the FO group than in NFO group (p<0.05).”<br>„Method: *Milk fat was determined by using the Crematocrit method as described by Lemons (Lemons et al. 1980)*”			

**Outcome 6**.		**Description**			
	**Outcome name**	Morbidity (results not reported)			
	**Outcome definition** *(with diagnostic criteria if relevant)*	Morbidity of mothers was assessed by interviewing the mothers weekly to obtain information on symptoms of selected illnesses during the previous 7 days. *(from Atalhii, 2020)*<br>“Ethical considerations precluded the use of a placebo group (unsupplemented control group).”			
	**Time point**<br>*(specify from start or end of intervention) for example 3,6,9,12*	weekly administration			
	**Unit of analysis**<br>*(individuals, cluster/ groups or body parts)*	FO group- NFO group			
	**Post-intervention or change from baseline?**	no data			
	**Unit of measurement**	–			
	**Results**	**Intervention group** FO group			
		**Time point**	**Mean**<br>*(if it Median than correct this part please to that)*	**SD**<br>*(or other variance, specify)*	**No. participants**
		**Comparison group** NFO group			
		**Time point**	**Mean**<br>*(if it Median than correct this part please to that)*	**SD**<br>*(or other variance, specify)*	**No. participants**
	**Any other results reported**<br>*(e.g. mean difference, CI, P value)*	–			

**Outcome 7**.		**Description**
	**Outcome name**	Proportion of mothers who consumed foods rich in vitamin A /dietary vitamin A intake by FFQ/

	**Outcome definition** *(with diagnostic criteria if relevant)*	dietary vitamin A intake: Usual consumption of foods containing vitamin A was assessed weekly by administering a food frequency questionnaire to mothers *(from Atalhi, 2020)*
	**Time point** *(specify from start or end of intervention) for example 3,6,9,12*	weekly administration
	**Unit of analysis** *(individuals, cluster/ groups or body parts)*	FO group- NFO group
	**Post-intervention or change from baseline?**	post-intervention
	**Unit of measurement**	frequency
	**Results**	**Intervention group** FO group
		**Time point** / **Mean** *(if it Median than correct this part please to that)* / **SD** *(or other variance, specify)* / **No. participants**
		– / – / – / –
		**Comparison group** NFO group
		**Time point** / **Mean** *(if it Median than correct this part please to that)* / **SD** *(or other variance, specify)* / **No. participants**
		– / – / – / –
	**Any other results reported** *(e.g. mean difference, CI, P value)*	**FFQ not showed different between FO and NFO except for** pumkin and liver where the percentage of women reporting their consumption was slightly higher in NFO group compared to FO, the difference being statistically significant (p = 0.03). There was no significant difference between the two groups for the rest of the food items included in the list." *(See in Text in Table 5)*

	**Conclusion**
**Conclusion**	"In conclusion, fortification approach seems to be an effective way to maintain adequate concentrations of VA in milk of lactating women until 6mo of post-partum." *Atalhi, 2019*<br><br>„1-Supplementation with a high dose of vitamin A has a positive impact on milk vitamin A concentration but was not sufficient to maintain adequate milk vitamin A levels throughout lactation. it has limited to the first 3 months of lactation.2- Fortification is considered a long-term strategy for sustaining and improving adequate human milk vitamin A concentration during the 1rst 6 month of lactation." *Abstract from Atalhi*

		**Description**
**Study Characteristics**	**Study ID**	#1314
	**Author, date**	**Ghasemifard 2020**
	**Publication type** *(e.g. full report, abstract, letter)*	full report, trial registry<br>Registry of Clinical Trials (IRCT.ir) with ID number of: IRCT20180708040401N1.
	**Language of publication**	English, Arab
	**Funding**	Supported by the Shiraz University of Medical Sciences under Grant number of 95-01-84-13932.
	**Conflict** *(notable conflicts of interest of study authors)*	None of the authors had any personal or financial conflicts of interest.
**Methods**	**Aim of study**	"The aim of this study was to compare the effect of vitamin D fortified oil consumption and vitamin D supplementation on serum 25-hydroxy vitamin D and bone turnover factors."<br><br>The purpose of the this study is to evaluate the effect of daily consumption of Canola oil enriched with 1000 IU of vitamin D on serum levels of hydroxycholecalciferol and fat profile in Shiraz adults.
	**Study design** *(RCT/ CCT/ cohort/ controlled before-after/ interrupted time series)*	double blind parallel randomized clinical trial
	**Unit of allocation** *(by individuals, cluster/ groups or body parts)*	Participants were randomly allocated.<br>"Balanced block randomization method was used for random allocation."
	**Number of study arms**	3
	**Number of study centres**	1
	**Start date of study**	October 2018
	**End date of study**	July 2019
**Participants**	**Population description**	Healthy, community-dwelling participants

	(from which study participants are drawn)	
	**Country where trial was performed**	Iran
	**Location/Setting**	Islamic Republic, Iran, Shiraz
	**Number of study centers**	1
	**Inclusion criteria**	18-30 years old<br>The absence of diseases which influence the metabolism of Ca, P and vitamin D such as thyroid and parathyroid disorders, chronic kidney disease, osteoporosis, or bone fractures caused by osteoporosis, during the past year<br>The absence of cardiovascular disease, diabetes, chronic digestive diseases, hepatitis and cancer<br>No medical treatment for osteoporosis or other bone diseases in the last 6 months, such as taking Bisphosphonates, Raloxifene, Teriparatide and Denosambe<br>Not using Glucocorticoids<br>Not using nutritional supplements<br>No pregnancy and lactation<br>Not participating in other studies in the last 6 months
	**Exclusion criteria**	• The presence of diseases affecting the metabolism of phosphorus, calcium and vitamin D such as thyroid and parathyroid disorders<br>• The presence of cardiovascular disease, diabetes, chronic diseases of the digestive tract, hepatitis, chronic kidney disease and cancer<br>• Medical treatment for osteoporosis or other bone diseases in the last 6 months<br>• Use of Bisphosphonates, Raloxifene, Teriparatide, Denosumab, and Glucocorticoids;<br>• Treatment with glucocorticoids<br>• Using nutritional supplements<br>• Pregnancy and lactating<br>• Participating in other studies in the last 6 months<br>• Unwilling to continue the study, no referral for blood sampling, and oil or supplement consumption less than 80%<br>• new insurance premiums
	**Number of participants**	Number of contacted persons: 120<br>Agreed to participate: 111<br>Sample size *(started the study)*: 99<br>Lost to follow-up/withdrawals/drop outs: 6<br>Number of participant analysed: 93<br>Sample size of total group: 93<br>Sample size of each groups: 31 (Vitamin D Supplement group), 30 (Vitamin D Enriched Oil group), 32 (Control group)
	**Sex**<br>*(Male/Female; Boys/Girls)*	male and female (female participants are in higher range- 80.6 % in Vitamin D supplementation group, 74.2 % in Vitamin D enriched oil group, 72.7 % in control group)
	**Mean age**<br>**(age range)**	25.70 ± 3.88 (Vitamin D supplement group), 24.83 ± 3.75 (Vitamin D enriched oil group), 25.60 ± 3.99 (Control group)
	**Race/Ethnicity**	no data
	**Other relevant baseline data**<br>*(e.g. BMI, health status, pregnant ect.)*	There were no significant differences in age, sex, education, height, weight, BMI, accommodation status, use of sunscreen, sun exposure score, smoking status, or physical activity level among the study groups at baseline.<br>"Considering the minimum difference in the mean serum level of 25-hydroxycholecalciferol between groups equal to 15 ng/ml, common standard deviation equal to 11 and having 3 groups"
	**Subgroups measure**	not applicable
	**Subgroups reported**	Participants who had serum 25-hydroxyvitamin D lower than 30 ng/ml.<br>Participants who had serum 25-hydroxyvitamin D higher than 30 ng/ml
**Interventions**	**Intervention(s)**	a placebo tablet + 25-gram canola oil enriched by 1000 IU vitamin D per day
	**Comparator(s)**	placebo tablet + 25 gram ordinary canola oil.
	**Dosage**<br>*(e.g recorded by FFQ)*	Participants were asked to use one tablet each day and add 25 g canola oil to their salad or baked foods every day.
	**Fortified oil**<br>*(e.g type, name ect.)*	canola oil (Kanwal oil was also purchased from Elden Golden Company,Iran)<br>vitamin D3 powder (40,000,000 IU/gram) used to fortify canola oil were purchased from Osvah pharmaceutical Company (Iran).<br>"Fortified oil are also completely the same as unfortified oil in terms of color and packaging."
	**Duration of intervention**	12 weeks
	**Duration of follow-up**	no data
	**Run-in period**	no data
	**Concomitant intervention**	Vitamin D Supplement group, taking a vitamin D tablet containing 1000 IU vitamin D + 25 g conventional canola oil

	**Timing** *(e.g. frequency, duration of each episode)*	not applicable

		Description
**Outcomes**	**Primary outcomes defined by the authors**	25-hydroxy vitamin D, collagen type 1 cross-linked C-telopeptide I, bone specific alkaline phosphatase, parathyroid hormone, energy and protein intake, vitamin D intake, Intake of vitamin K, vitamin C, calcium, phosphorus, magnesium, zinc, Total cholesterol (CHOL) low density lipoprotein (LDL); high density lipoprotein (HDL); Triglyceride (TG)
	**Secondary outcomes defined by the authors**	-
	**Confounder** *(uncontrolled, but not adjusted; list of variables authors adjusted variables; Methods used to control to confounders)*	sun exposure, physical activity, body mass index, dietary intakes (Energy, protein, vitamin D, vitamin C, vitamin K, Calcium, Phosphorus, Magnesium, Zinc), parathyroid hormone, smoking

		Description			
**Outcome 1.**	**Outcome name**	25-hydroxyvitamin D			
	**Outcome definition** *(with diagnostic criteria if relevant)*	Serum 25(OH)D level was measured from fasting blood.			
	**Time point** *(specify from start or end of intervention) for example 3,6,9,12*	0, 12 weeks			
	**Unit of analysis** *(individuals, cluster/ groups or body parts)*	individuals, subgroups			
	**Post-intervention or change from baseline?**	post intervention, mean differences			
	**Unit of measurement**	ng/ml			
	**Results**	**Intervention group**			
		**Time point**	**Mean**	**SD**	**No. participants**
		Baseline	30.8	10.66	30
		After intervention	33.65	10.28	30
		**Comparison group**			
		**Time point**	**Mean**	**SD**	**No. participants**
		Baseline	31.62	14.32	32
		After intervention	31.01	11.41	32
	**Vitamin D deficient subgroup (serum 25-hydroxyvitamin D <30 ng/ml)** **Results**	**Intervention group**			
		**Time point**	**Mean**	**SD**	**No. participants**
		Baseline	20.21	7.02	12
		After intervention	24.15	8.57	12
		**Comparison group**			
		**Time point**	**Mean**	**SD**	**No. participants**
		Baseline	18.31	7.34	14
		After intervention	21.75	6.04	14
	**Vitamin D sufficient subgroup (serum 25-hydroxyvitamin D > 30 ng/ml)** **Results**	**Intervention group**			
		**Time point**	**Mean**	**SD**	**No. participants**
		Baseline	37.86	5.49	18
		After intervention	39.99	5.15	18
		**Comparison group**			
		**Time point**	**Mean**	**SD**	**No. participants**
		Baseline	41.98	8.57	18
		After intervention	38.22	9.15	18
	**Any other results reported** *(e.g. mean difference, CI, P value)*	Mean differences:<br>**Total population**: Intervention group: 2.85 ± 4.69; Comparison group: -0.6 ± 7.64<br>Differences between baseline and after intervention (p value): Intervention group: 0,002; Comparison group: 0.65<br>**Vitamin D deficient subgroup**:<br>Intervention group: 3.93 ± 6.48; Comparison group: 3.43 ± 7.34<br>Differences between baseline and after intervention (p value): Intervention group: 0,05; Comparison group: 0.1<br>**Vitamin D sufficient subgroup**<br>Intervention group: 2.13 ± 2.99; Comparison group: -3.75 ± 6.43<br>Differences between baseline and after intervention (p value): Intervention group: 0.008; Comparison group: 0.02			

		Description
**Outcome 2.**	**Outcome name**	Parathyroid hormone (PTH)

**Outcome definition** *(with diagnostic criteria if relevant)*	Fasting serum PTH level was measured.			
**Time point** *(specify from start or end of intervention) for example 3,6,9,12*	0, 12 weeks			
**Unit of analysis** *(individuals, cluster/ groups or body parts)*	individuals, subgroups			
**Post-intervention or change from baseline?**	post intervention, mean differences			
**Unit of measurement**	pg/m			
**Results**	**Intervention group**			
	**Time point**	**Mean**	**SD**	**No. participants**
	Baseline	24.25	14.58	
	After intervention	27.29	11.20	
	**Comparison group**			
	**Time point**	**Mean**	**SD**	**No. participants**
	Baseline	18.48	10.67	
	After intervention	26.34	19.24	
**Vitamin D deficient subgroup (serum 25-hydroxyvitamin D <30 ng/ml)** **Results**	**Intervention group**			
	**Time point**	**Mean**	**SD**	**No. participants**
	Baseline	23.99	18.2	
	After intervention	28.15	11.26	
	**Comparison group**			
	**Time point**	**Mean**	**SD**	**No. participants**
	Baseline	22.81	11.29	
	After intervention	29.19	22.6	
**Vitamin D sufficient subgroup (serum 25-hydroxyvitamin D > 30 ng/ml)** **Results**	**Intervention group**			
	**Time point**	**Mean**	**SD**	**No. participants**
	Baseline	24.43	12.17	
	After intervention	26.71	11.45	
	**Comparison group**			
	**Time point**	**Mean**	**SD**	**No. participants**
	Baseline	15.11	9.09	
	After intervention	24.13	16.5	
**Any other results reported** *(e.g. mean difference, CI, P value)*	**Total population**:<br>Mean differences: Intervention group:3.03 ± 11.21; Comparison group: 7.86 ± 14.82<br>Differences between baseline and after intervention (p value): Intervention group: 0.01; Control group: 0.003<br>**Vitamin D deficient subgroup**<br>Mean differences: Intervention group: 4.15 ± 15.62; Comparison group: 6.38 ± 15.91<br>Differences between baseline and after intervention (p value): Intervention group: 0.03; Comparison group: 0.15<br>**Vitamin D sufficient subgroup:**<br>Mean differences: Intervention group:2.28 ± 7.41; Comparison group: 9.01 ± 14.28<br>Differences between baseline and after intervention (p value): Intervention group: 0.23; Control group: 0.005			

**Outcome 3.**		**Description**			
	**Outcome name**	Bone alkaline phosphatase (B-ALP)			
	**Outcome definition** *(with diagnostic criteria if relevant)*	Serum B-ALP was measured			
	**Time point** *(specify from start or end of intervention) for example 3,6,9,12*	0, 12 weeks			
	**Unit of analysis** *(individuals, cluster/ groups or body parts)*	individuals, subgroups			
	**Post-intervention or change from baseline?**	post intervention, mean differences			
	**Unit of measurement**	UI/L			
	**Results**	**Intervention group**			
		**Time point**	**Mean**	**SD**	**No. participants**
		Baseline	45.58	11.1	
		After intervention	46.92	8.15	
		**Comparison group**			

		Time point	Mean	SD	No. participants
		Baseline	40.91	9.24	
		After intervention	41.16	9.78	
	**Vitamin D deficient subgroup (serum 25-hydroxyvitamin D <30 ng/ml)** **Results**	**Intervention group**			
		**Time point**	**Mean**	**SD**	**No. participants**
		Baseline	41.74	12.22	
		After intervention	44.96	8.81	
		**Comparison group**			
		**Time point**	**Mean**	**SD**	**No. participants**
		Baseline	40.75	8.9	
		After intervention	40.41	10.3	
	**Vitamin D sufficient subgroup (serum 25-hydroxyvitamin D > 30 ng/ml)** **Results**	**Intervention group**			
		**Time point**	**Mean**	**SD**	**No. participants**
		Baseline	48.13	9.81	
		After intervention	48.23	7.65	
		**Comparison group**			
		**Time point**	**Mean**	**SD**	**No. participants**
		Baseline	41.03	9.76	
		After intervention	41.75	9.61	
	**Any other results reported** *(e.g. mean difference, CI, P value)*	**Total population**:<br>Mean differences: Intervention group:1.34 ± 7.69; Comparison group: 0.25 ± 4.78<br>Differences between baseline and after intervention (p value): Intervention group: 0.10; Control group: 0.51<br>**Vitamin D deficient subgroup**<br>Mean differences: Intervention group: 3.22 ± 5.87; Comparison group: -0.34 ± 6.18<br>Differences between baseline and after intervention (p value): Intervention group: 0.05; Comparison group: 0.82<br>**Vitamin D sufficient subgroup:**<br>Mean differences: Intervention group:0.09 ± 8.63; Comparison group: 0.71 ± 3.45<br>Differences between baseline and after intervention (p value): Intervention group: 0.61; Control group: 0.32			

**Outcome 4.**		**Description**			
	**Outcome name**	Collagen type 1 cross-linked C-telopeptide (CTX)			
	**Outcome definition** *(with diagnostic criteria if relevant)*	Serum CTX was measured			
	**Time point** *(specify from start or end of intervention) for example 3,6,9,12*	0, 12 weeks			
	**Unit of analysis** *(individuals, cluster/ groups or body parts)*	individuals, subgroups			
	**Post-intervention or change from baseline?**	post intervention, mean differences			
	**Unit of measurement**	pg/ml			
	**Results**	**Intervention group**			
		**Time point**	**Mean**	**SD**	**No. participants**
		Baseline	3212.59	4703.06	
		After intervention	3046.89	4535.83	
		**Comparison group**			
		**Time point**	**Mean**	**SD**	**No. participants**
		Baseline	3175.65	4732.37	
		After intervention	3459.88	4702.39	
	**Vitamin D deficient subgroup (serum 25-hydroxyvitamin D <30 ng/ml)** **Results**	**Intervention group**			
		**Time point**	**Mean**	**SD**	**No. participants**
		Baseline	4918.7	6717.31	
		After intervention	4793.86	6660.32	
		**Comparison group**			
		**Time point**	**Mean**	**SD**	**No. participants**
		Baseline	3077.32	2875.47	
		After intervention	3095.2	2926.34	
	**Vitamin D sufficient subgroup (serum 25-hydroxyvitamin D > 30 ng/ml)** **Results**	**Intervention group**			
		**Time point**	**Mean**	**SD**	**No. participants**
		Baseline	2075.18	2260.73	
		After intervention	1882.25	1674.07	
		**Comparison group**			

		Time point	Mean	SD	No. participants
		Baseline	3252.13	5873.8	
		After intervention	3743.53	5794.78	
	**Any other results reported** *(e.g. mean difference, CI, P value)*	**Total population**:<br>Mean differences: Intervention group: -165.69 ± 940.17; Comparison group: 284.23 ± 1757.76<br>Differences between baseline and after intervention (p value): Intervention group: 0.26; Control group: 0.58<br>**Vitamin D deficient subgroup**<br>Mean differences: Intervention group: -124.83 ± 1138.16; Comparison group: 17.87 ± 991.45<br>Differences between baseline and after intervention (p value): Intervention group: 0.63; Comparison group: 0.82<br>**Vitamin D sufficient subgroup:**<br>Mean differences: Intervention group:-192.93 ± 817.13; Comparison group: 491.4 ± 2186.01<br>Differences between baseline and after intervention (p value): Intervention group: 0.26; Control group: 0.32			

		**Description**			
**Outcome 4.-5.**	**Outcome name**	energy and protein intake			
	**Outcome definition** *(with diagnostic criteria if relevant)*	-			
	**Time point** *(specify from start or end of intervention) for example 3,6,9,12*	0, 12 weeks			
	**Unit of analysis** *(individuals, cluster/ groups or body parts)*	individuals, subgroups			
	**Post-intervention or change from baseline?**	post intervention, mean differences			
	**Unit of measurement**	Energy (kcal), Protein (g)			
	**Results Energy intake**	**Intervention group**			
		**Time point**	**Mean**	**SD**	**No. participants**
		Baseline	1461.58	899.45	30
		After intervention	1364.2	465.32	30
		Mean differences	39.17	495.98	30
		**Comparison group**			
		**Time point**	**Mean**	**SD**	**No. participants**
		Baseline	1416.33	526.5	32
		After intervention	1644.2	471.78	32
		Mean differences	332.3	578.94	32
	**Results Protein intake**	**Intervention group**			
		**Time point**	**Mean**	**SD**	**No. participants**
		Baseline	45.19	19.64	30
		After intervention	53.67	38.73	30
		Mean differences	7.9	37.91	30
		**Comparison group**			
		**Time point**	**Mean**	**SD**	**No. participants**
		Baseline	73.31	105.39	32
		After intervention	51.32	16.01	32
		Mean differences	-4.76	45.44	32
	**Any other results reported**	There were no significant differences in energy, protein, vitamin D, K, C, calcium, phosphorus, magnesium, and zinc intakes changes among the 3 groups, during the intervention (P > 0.05).			

		**Description**
**Outcome 6.-12.**	**Outcome name**	Intake of vitamin D, vitamin K, vitamin C, calcium, phosphorus, magnesium, zinc
	**Outcome definition** *(with diagnostic criteria if relevant)*	-
	**Time point** *(specify from start or end of intervention) for example 3,6,9,12*	0, 12 weeks
	**Unit of analysis** *(individuals, cluster/ groups or body parts)*	individuals, subgroups
	**Post-intervention or change from baseline?**	post intervention, mean differences

	**Unit of measurement**	vitamin D (mcg), vitamin K (mcg), vitamin C (mg), calcium (mg), phosphorus (mg), magnesium (mg), zinc (mg)			
	**Results** vitamin D	**Intervention group**			
		**Time point**	**Mean**	**SD**	**No. participants**
		Baseline	0.91	1.51	30
		After intervention	0.81	1.37	30
		Mean differences	-0.15	1.69	30
		**Comparison group**			
		**Time point**	**Mean**	**SD**	**No. participants**
		Baseline	0.42	0.9	32
		After intervention	23.16	88.09	32
		Mean differences	26.47	93.9	32
	**Results** vitamin K	**Intervention group**			
		**Time point**	**Mean**	**SD**	**No. participants**
		Baseline	49.2	50.39	30
		After intervention	49.73	54.18	30
		Mean differences	-4.16	61.83	30
		**Comparison group**			
		**Time point**	**Mean**	**SD**	**No. participants**
		Baseline	137.48	297.77	32
		After intervention	56.67	56.2	32
		Mean differences	-168.87	409.14	32
	**Results** vitamin C	**Intervention group**			
		**Time point**	**Mean**	**SD**	**No. participants**
		Baseline	105.42	97.2	30
		After intervention	78.7	90.78	30
		Mean differences	-27.05	102.05	30
		**Comparison group**			
		**Time point**	**Mean**	**SD**	**No. participants**
		Baseline	143.29	102.44	32
		After intervention	140.77	107.6	32
		Mean differences	-5.6	182.57	32
	**Results** calcium	**Intervention group**			
		**Time point**	**Mean**	**SD**	**No. participants**
		Baseline	429.44	234.42	30
		After intervention	442.6	352.96	30
		Mean differences	18.96	349.96	30
		**Comparison group**			
		**Time point**	**Mean**	**SD**	**No. participants**
		Baseline	758.82	1675.08	32
		After intervention	353.18	161.68	32
		Mean differences	-115.57	316.82	32
	**Results** phosphorus	**Intervention group**			
		**Time point**	**Mean**	**SD**	**No. participants**
		Baseline	708.5	359.39	30
		After intervention	1247.55	2422.15	30
		Mean differences	519.51	2331.88	30
		**Comparison group**			
		**Time point**	**Mean**	**SD**	**No. participants**
		Baseline	779.65	584.99	32
		After intervention	698.26	154.3	32
		Mean differences	-167.51	788.66	32
	**Results** magnesium	**Intervention group**			
		**Time point**	**Mean**	**SD**	**No. participants**
		Baseline	160.61	72.35	30
		After intervention	178.74	115.77	30
		Mean differences	22	110.25	30
		**Comparison group**			
		**Time point**	**Mean**	**SD**	**No. participants**
		Baseline	228.26	264.2	32
		After intervention	198.48	65.28	32
		Mean differences	-8.06	212.25	32
	**Results** zinc	**Intervention group**			
		**Time point**	**Mean**	**SD**	**No. participants**
		Baseline	4.85	2.04	30
		After intervention	5.63	3.22	30

		Mean differences	0.84	3.03	30
		**Comparison group**			
		**Time point**	**Mean**	**SD**	**No. participants**
		Baseline	7.55	8.21	32
		After intervention	6.11	2.42	32
		Mean differences	-0.34	4.34	32
	**Any other results reported** *(e.g. mean difference, CI, P value)*	There were no significant differences in energy, protein, vitamin D, K, C, calcium, phosphorus, magnesium, and zinc intakes changes among the 3 groups, during the intervention (P > 0.05).			

**Outcome 13.-16.**		**Description**			
	**Outcome name**	Total cholesterol (CHOL) low density lipoprotein (LDL); high density lipoprotein (HDL); Triglyceride (TG)			
	**Outcome definition** *(with diagnostic criteria if relevant)*	-			
	**Time point** *(specify from start or end of intervention) for example 3,6,9,12*	0, 12 weeks			
	**Unit of analysis** *(individuals, cluster/ groups or body parts)*	individuals, subgroups			
	**Post-intervention or change from baseline?**	post intervention, mean differences			
	**Unit of measurement**	CHOL (mg/dl), LDL (mg/dl), HDL (mg/dl), TG (mg/dl)			
	**Results** CHOL	**Intervention group**			
		**Time point**	**Mean**	**SD**	**No. participants**
		Baseline	147.03	29.01	30
		After intervention	150.23	32.96	30
		Mean differences	3.2	15.65	30
		**Comparison group**			
		**Time point**	**Mean**	**SD**	**No. participants**
		Baseline	154.78	10.25	32
		After intervention	149.87	27.79	32
		Mean differences	-4.9	21.86	32
	**Results** LDL	**Intervention group**			
		**Time point**	**Mean**	**SD**	**No. participants**
		Baseline	78.86	19.91	30
		After intervention	84.96	23.08	30
		Mean differences	6.10	11.88	30
		**Comparison group**			
		**Time point**	**Mean**	**SD**	**No. participants**
		Baseline	85.28	18.61	32
		After intervention	84.25	18.80	32
		Mean differences	-1.03	14.93	32
	**Results** HDL	**Intervention group**			
		**Time point**	**Mean**	**SD**	**No. participants**
		Baseline	45.76	8.63	30
		After intervention	44.90	6.96	30
		Mean differences	-0.86	6.28	30
		**Comparison group**			
		**Time point**	**Mean**	**SD**	**No. participants**
		Baseline	46.21	9.68	32
		After intervention	43.78	9.02	32
		Mean differences	-2.43	6.06	32
	**Results** TG	**Intervention group**			
		**Time point**	**Mean**	**SD**	**No. participants**
		Baseline	145.40	136.59	30
		After intervention	115.73	66.42	30
		Mean differences	-29.66	100.79	30
		**Comparison group**			
		**Time point**	**Mean**	**SD**	**No. participants**
		Baseline	137.81.	91.10	32
		After intervention	113.96	61.82	32
		Mean differences	-23.84	56.08	32
	**Any other results reported**	-			

Conclusion	Conclusion
	„Consumption of 1000 IU vitamin D per day for 12 weeks as a supplement or fortified oil could enhance the serum vitamin D in main population. However, the protective effect of vitamin D supplementation and oil fortification was seen just in vitamin D sufficient subgroup, not vitamin D deficient one. Besides, this dose of vitamin did not have a noticeable effect on bone turnover markers in this period."

		Description	
Study Characteristics	**Study ID**	#2073	
	**Author, date**	**Keller, 2017**	
	**Publication type** *(e.g. full report, abstract, letter)*	full report	
	**Language of publication**	English	
	**Funding**	„The study is a part of the four-year project 'D-tect' funded by the Programme Commission on Health, Food and Welfare under the Danish Council for Strategic research (grant no. 0603- 00453B). Financial support has further been granted by the Danish Diabetes Academy supported by the Novo Nordisk Foundation and The Lundbeck Foundation (grant no. R170- 2014-643)."	
	**Conflict** *(notable conflicts of interest of study authors)*	"The authors declare that there are no conflicts of interest."	
Methods	**Aim of study**	"The aim of the present study was to analyse whether exposure during fetal life, to extra vitamin A from food fortification, was related to subsequent risk of developing type 2 diabetes mellitus (T2DM) in adulthood (before age 49 years)."	
	**Study design** *(RCT/ CCT/ cohort/ controlled before-after/ interrupted time series)*	prospective, birth cohort	
	**Unit of allocation** *(by individuals, cluster/ groups or body parts)*	groups	
	**Number of study arms**	2 (exposed, less-exposed)<br>higher prenatal vitamin A exposure (born 1 December 1962–31 March 1964) n=101.178<br>lower prenatal exposure (born 1 September 1959–31 December 1960) n=92.625	
	**Number of study centres**	not applicable	
	**Start date of study**	not applicable	
	**End date of study**	not applicable	
Participants	**Population description** *(from which study participants are drawn)*	Total population born in Denmark between September 1959–December 1960 and December 1962–March 1962<br>extracted from the Danish Civil Registration System	
	**Country where trial was performed**	Denmark	
	**Location/Setting**	-	
	**Inclusion criteria**	born in Denmark between September 1959–December 1960 and December 1962–March 1962	
	**Exclusion criteria**	Loss to follow-up<br>Death<br>"Individuals born in 1961 were excluded as it is unclear whether the increase in vitamin A added to margarine already became partially effective between 1961 and 1962."	
	**Number of participants**	Number of contacted persons	not applicable
		Agreed to participate	not applicable
		Sample size *(started the study)*	193803
		Lost to follow-up/withdrawals/drop outs	loss to follow up: 3188<br>death: 3978 (exposed: n=1947, less-exposed: n=2032)
		Number of participant analysed	180447
		Sample size of total group	-
		Sample size of each groups	Exposed: 94131<br>less exposed: 86316
	**Sex** *(Male/Female; Boys/Girls)*	male (52 %) and female (48 %)	
	**Mean age (age range)**	not applicable	
	**Race/Ethnicity**	not defined	
	**Other relevant baseline data** *(e.g. BMI, health status, pregnant ect.)*	not defined	
	**Subgroups measure**	age groups for developing T2DM	
	**Subgroups reported**	age groups for developing T2DM	

**Interventions**	**Intervention(s)**	Fortification of margarine with vitamin D and higher dose of vitamin A (vitamin A added to margarine by 25 % from 4.2 μg/g of retinol and 3.6 μg/g of β-carotene (equivalent to 0·6 % of the current RDA) to 6 μg/g of retinol and 3 μg/g of β-carotene (equivalent to 0.8 % of the current RDA)
	**Comparator(s)**	Fortification of margarine with vitamin D and less dose of vitamin A
	**Dosage** *(e.g recorded by FFQ)*	"Data from food disappearance show that an average of 18 kg margarine/person per year was bought in Denmark in the 1960s, suggesting that personal daily intake from fortified margarine increased from 207 to 296 μg of retinol and decreased from 178 to 148 μg of β-carotene"
	**Fortified oil** *(e.g type, name ect.)*	margarine
	**Duration of intervention**	not applicable
	**Duration of follow-up**	not applicable<br>„...were followed up with regard to development of T2DM before 31 December 2012 in the Danish National Diabetes Registry and National Patient Register."<br>"A total of 193 803 individuals were followed up until midlife."
	**Run-in period**	not applicable
	**Concomitant intervention**	"Fortification of margarine with vitamins A and D became compulsory in Denmark in 1937."
	**Timing** *(e.g. frequency, duration of each episode)*	not applicable

		Description
**Outcomes**	**Primary outcomes defined by the authors**	T2DM cases
	**Secondary outcomes defined by the authors**	Other diabetes
	**Confounder** *(uncontrolled, but not adjusted; list of variables authors adjusted variables; Methods used to control to confounders)*	-

		Description		
**Outcome 1.**	**Outcome name**	T2DM cases		
	**Outcome definition** *(with diagnostic criteria if relevant)*	Individuals were defined as T2DM cases if they were alive, at risk and diabetes free until age 36 years and had a first diagnosis of diabetes corresponding to International Classification of Diseases (ICD) 8 code 250 or ICD 10 code E11 between 36 years and 48 years and 9 months (48-75 years) of age. In addition, individuals were also classified as having T2DM when one of the following criterion as first diagnosis, combined with a following diagnosis of ICD 10 code E11, was met(:<br>• chiropody for diabetic patient; ·<br>• date of fifth blood glucose measurement within a year;<br>• second purchase of oral glucose-lowering drugs;<br>• second purchase of insulin.		
	**Time point** *(specify from start or end of intervention) for example 3,6,9,12*	not applicable		
	**Unit of analysis** *(individuals, cluster/ groups or body parts)*	individuals		
	**Post-intervention or change from baseline?**	not applicable		
	**Unit of measurement**	%		
	**Results**	**Intervention group (Exposed)**		
		**Number of participants**	**Number of individuals with T2DM**	**%**
		94131	1273	1.35
		**Comparison group (Less-exposed)**		
		**Number of participants**	**Number of individuals with T2DM**	**%**
		86316	1322	1.53
	**Any other results reported** *(e.g. mean difference, CI, P value)*	"The mean age for developing T2DM was 43.2 (SD 3.5) and 43.6 (SD 3.4) years in the exposed and less-exposed groups, respectively (P=0.15)."<br>The individuals exposed to higher vitamin A from fortification were less likely to develop T2DM (OR 0·88; 95 % CI 0·81, 0·95, P= 0·001)<br>Women were generally at a lower risk of developing T2DM than men (OR 0·70; 95 % CI 0·65, 0·76, P<0·001). However, there was no interaction between exposure status and sex (OR 0·94; 95 % CI 0·81, 1·10, P=0·47), suggesting that substantial effect modification by sex was unlikely.		

Outcome 2.		Description		
	**Outcome name**	Other diabetes case		
	**Outcome definition** *(with diagnostic criteria if relevant)*	not defined		
	**Time point** *(specify from start or end of intervention) for example 3,6,9,12*	not applicable		
	**Unit of analysis** *(individuals, cluster/ groups or body parts)*	individuals		
	**Post-intervention or change from baseline?**	not applicable		
	**Unit of measurement**	%		
	**Results**	**Intervention group (Exposed)**		
		**Number of participants**	**Number of individuals with T2D**	**%**
		94131	2075	2.2
		**Comparison group (Less-exposed)**		
		**Number of participants**	**Number of individuals with T2D**	**%**
		86316	1519	1.76
	**Any other results reported** *(e.g. mean difference, CI, P value)*			

Conclusion	Conclusion
	„This study suggests that fetal exposure to extra vitamin A from fortified margarine may have lowered the risk of developing T2DM in adulthood, and that this effect was similar for men and women and stronger for those developing T2DM at an older age."

		Description
**Study Characteristics**	**Study ID**	#3986
	**Author, date**	**Stougaard, 2018**
	**Publication type** *(e.g. full report, abstract, letter)*	Full report
	**Language of publication**	English
	**Funding**	"Funded by the Danish Agency for Science, Technology and Innovation, the Ministry of Science, Higher Education, under the instrument "Strategic Research Projects" (grant previous 11-116213 now 0603-00453B) and by the PhD School of Faculty of Health Sciences the University of Southern Denmark."
	**Conflict** *(notable conflicts of interest of study authors)*	None
**Methods**	**Aim of study**	„The main objective of the present study was to investigate if pregnant women who received small, but consistent extra vitamin D doses from food fortification had a lower risk of developing preeclampsia than pregnant women who did not receive this extra vitamin D."
	**Study design** *(RCT/ CCT/ cohort/ controlled before-after/ interrupted time series)*	Birth cohort study
	**Unit of allocation** *(by individuals, cluster/ groups or body parts)*	group
	**Number of study arms**	2<br>Exposed group - unexposed group
	**Number of study centres**	National data sources<br><br>The Danish Civil Registration System (CSR), Denmark with a 10-digit civil person register (CPR), The Danish Medical Birth Registry (MBR), The Danish National Patient Registry (DNPR)
	**Start date of study**	1st of June 1983
	**End date of study**	31st of August 1988
**Participants**	**Population description** *(from which study participants are drawn)*	Nulliparous women, who gave births from the 1st of June 1983 to the 31st of May 1985 (exposed group);<br><br>and births from the 1st of September 1986 to the 31st of August 1988 (unexposed group)
	**Country where trial was performed**	Denmark
	**Location/Setting**	national data, Denmark
	**Inclusion criteria**	„All women in Denmark who gave birth in the period from the 1st of June 1983 to the 31st of August 1988—in total 284,179 births"

		"The study population was further restricted only to include nulliparous women who gave birth after 22 completed gestational weeks"	
	**Exclusion criteria**	„68,271 births that occurred during the wash-out period were excluded"<br>"excluded births with misclassification of the offspring's birth weight, as well as those with missing information on maternal age at delivery, gestational age, single or multiple pregnancy, birth weight or gender of offspring"	
	**Number of participants**	Number of contacted persons	no contacts, national data
		Agreed to participate	–<br><br>national data<br><br>"The Danish Data Protection Agency (J.no. 2012-41-1156) approved data access and retrieval from the registries. As the study was based on already collected data, approval from the ethical committee was not required."
		Sample size<br>*(started the study)*	284 179
		Lost to follow-up/withdrawals/dropouts	Women excluded: 68 271<br>*„Deliveries excluded during the 15 months' wash-out period from June 1985 to August 1986."*<br><br>Excluded deliveries: 67 357+ 74 505= 141862<br>*„Births which were not the women first, misclassification of the offspring's birth weight, or gestational weeks was <22 weeks."*<br>Excluded deliveries:<br>289+ 340= 629<br>*„Excluded due to missing information on maternal age at delivery, singleton and multiple births, gestational age at delivery, birth weight or offspring gender."*<br>Total: 210 762
		Number of participants analysed	73,237<br>(analyzed exposed women 35 124+ analysed unexposed women 38 113)
		Sample size of total group	73,237
		Sample size of each group	Exposed: 35124<br>Unexposed: 38113<br><br>Pregnancy related hypertension complication among analyzed exposed women: 1,976<br><br>Pregnancy related hypertension complication among analyzed unexposed women: 2,061<br><br>• Group 1: Gestational hypertension exposed n=399 unexposed n=396<br>• Group 2: Mild preeclampsia, Preeclampsia, unspecified & Toxemia exposed n=1310 and unexposed n=1370<br>• Group 3: Severe preeclampsia, Eclampsia, exposed n=267 unexposed n=295
	**Sex**<br>*(Male/Female; Boys/Girls)*	Female<br>Offspring (male, female)	
	**Mean age**<br>**(age range)**	not mentioned	
	**Race/Ethnicity**	not mentioned	

	**Other relevant baseline data** *(e.g. BMI, health status, pregnant ect.)*	Baseline characteristics:<br>age at delivery<br>gender of new-born/offspring (female, male)<br>singleton or multiple pregnancy<br>smoking status during pregnancy<br>season of delivery<br><br>„The distribution of hypertensive syndromes by their severity between the exposed and unexposed women did not differ (p = 0.2). Unexposed women were older than the exposed women (p<0.0001). Other characteristics of the unexposed and exposed women or their offspring did not differ."
	**Subgroups measure**	Gestational hypertension, Mild and unspecified preeclampsia or toxemia, Severe preeclampsia and eclampsia
	**Subgroups reported**	smoking and nonsmoking women; by type of pre-eclampsia
**Interventions**	**Intervention(s)**	Mandatory vitamin D fortification of margarine in Denmark until June 1985
	**Comparator(s)**	No mandatory fortification of margarine in Denmark
	**Dosage** *(e.g recorded by FFQ)*	1.25µg of vitamin D per 100g margarine
	**Fortified oil** *(e.g type, name ect.)*	Margarine<br><br>„A Danish fortification policy that required adding of 1.25µg of vitamin D per 100g margarine, corresponding to approximately 13% (3–29%) of the daily vitamin D intake, was terminated June 1st 1985"
	**Duration of intervention**	-<br>(from June 1983 to August 1988)
	**Duration of follow-up**	-
	**Run-in period**	„15 months' 'wash-out period' (consisting of a full 9 months of pregnancy and additional 6 months, securing that the fortified margarine was no longer available at home or in stores) was introduced and lasted from the 1st of June 1985 to the 31st of August 1986"
	**Concomitant intervention**	"During the study period margarine was also fortified with vitamin A, and the new evidence reveals that the vitamin A dose was increased by 25 % (from 20 to 25 IU/g) in 1962. There were also minor increases in 1971 from 25 to 26 IU/g and in 1985 from 26 to 28 IU/g." (Jensen, 2016)
	**Timing** *(e.g. frequency, duration of each episode)*	–<br>15-month wash-out period from June 1985 to September 1986.

		Description
**Outcomes**	**Primary outcomes defined by the authors**	„Definition of gestational hypertension, preeclampsia and eclampsia by ICD8 codes"<br><br>Group 1 Gestational hypertension (code O13.9)<br>Group 2 Mild to moderate pre-eclampsia (code O14.0)<br>Pre-eclampsia, unspecified (code O14.9)<br>Group 3 Severe pre-eclampsia (code O14.1)<br>HELLP syndrome (code O14.2)<br>Eclampsia in pregnancy (code O15.0)<br>Eclampsia in labour (code O15.2)<br>Eclampsia, unspecified as to time period (O15.9)
	**Secondary outcomes defined by the authors**	–
	**Confounder** *(uncontrolled, but not adjusted; list of variables authors adjusted variables; Methods used to control to confounders)*	"Pregnancies were categorized as either singletons or multiple pregnancies."<br>"Gender of the offspring, male vs. female, was considered in the analyses"<br>"Months of delivery were categorized into 4 seasons: November to January (Winter); February to April (Spring); May to July (Summer) and August to October (Fall)"

		Description
**Outcomes**	**Outcome name**	Gestational hypertension
	**Outcome definition (with diagnostic criteria if relevant)**	Group 1 Gestational hypertension (code O13.9)
	**Time point (specify from start or end of intervention) for example 3,6,9,12**	–
	**Unit of analysis (individuals, cluster/ groups or body parts)**	group<br>Exposed group - unexposed group

	**Post-intervention or change from baseline?**	–			
	**Unit of measurement**	N, n, %			
	**Results**	**Intervention group** Exposed			
		**Time point**	**Mean** *(if it Median than correct this part please to that)*	**SD** *(or other variance, specify)*	**No. participants**
					N = 35,124<br>n= 399<br>1.1 %<br>p=0.2
		**Comparison group** Unexposed			
		**Time point**	**Mean** *(if it Median than correct this part please to that)*	**SD** *(or other variance, specify)*	**No. participants**
					N = 38,113<br>396<br>1.0 %<br>p=0.2
	**Unit of measurement**	n, OR(95%CI)			
		**Exposed and Unexposed**			
		Crude and adjusted odds ratio for the risk of pregnancy related hypertensive disorders among women exposed vs unexposed to extra vitamin D during their pregnancy.	Crude model OR (95%CI)		
			1.09(0.95,1.26)		n=795
			Adjusted model OR (95%CI)		
			1.11(0.97,1.28)		n=795
	**Any other results reported** *(e.g. mean difference, CI, P value)*				

		**Description**			
**Outcome 1.**	**Outcome name**	"risk of preeclampsia"			
	**Outcome definition** *(with diagnostic criteria if relevant)*	Group 2: Mild preeclampsia (code 63703)<br>Preeclampsia, unspecified (code 63709)<br>Toxemia (63799)<br><br>Group 3:<br>Severe preeclampsia (code 63704)<br>Eclampsia (code 63719)			
	**Time point** *(specify from start or end of intervention) for example 3,6,9,12*	–			
	**Unit of analysis** *(individuals, cluster/ groups or body parts)*	group<br>Exposed group - unexposed group			
	**Post-intervention or change from baseline?**	–			
	**Unit of measurement**	N, n, %			
	**Results**	**Intervention group**			
		**Time point**	**Mean** *(if it Median than correct this part please to that)*	**SD** *(or other variance, specify)*	**No. participants**
		Group 2			N = 35,124<br>n= 1,310<br>3.7 %
		Group 3			N = 35,124<br>n= 267<br>0.8 %
		All Cases			N = 35,124<br>n= 1976<br>5.6 %
		**Comparison group**			
		**Time point**	**Mean** *(if it Median than correct this part please to that)*	**SD** *(or other variance, specify)*	**No. participants**

		Group 2		N = 35,124<br>n= 1,370<br>3.6 %
		Group 3		N = 35,124<br>n= 295<br>0.8 %
		All Cases		N = 35,124<br>n= 2061<br>5.4 %<br>p=0.2
	**Unit of measurement**	OR (95%CI)		
		**Exposed vs unexposed**		
	Group 2	Crude and adjusted odds ratio for the risk of pregnancy related hypertensive disorders among women exposed vs unexposed to extra vitamin D during their pregnancy.	Crude model OR (95%CI)	
			1.04(0.96,1.12)	n=2680
			Adjusted model OR (95%CI)	
			1.03(0.95,1.11)	n=2680
	Group 3	Crude and adjusted odds ratio for the risk of pregnancy related hypertensive disorders among women exposed vs unexposed to extra vitamin D during their pregnancy.	Crude model OR (95%CI)	
			0.98(0.83,1.16)	n=562
			Adjusted model OR (95%CI)	
			0.98(0.83,1.16)	n=562
	All cases	Crude and adjusted odds ratio for the risk of pregnancy related hypertensive disorders among women exposed vs unexposed to extra vitamin D during their pregnancy.	Crude model OR (95%CI)	
			1.04(0.98,1.11)	n= 4,037
			Adjusted model OR (95%CI)	
			1.04(0.98,1.10)	n= 4,037
	**Any other results reported** *(e.g. mean difference, CI, P value)*	"no associations between the exposure status and gestational hypertension or severe preeclampsia and eclampsia."<br>„women who gave birth during summer and fall (May to October) had the lowest risk of mild±unspecified preeclampsia, or toxemia"<br>„ between exposure status and season of preeclampsia diagnosis did not reveal any consisting findings"		

	**Conclusion**
**Conclusion**	„In conclusion, the extra vitamin D from the mandatory vitamin D fortification did not influence the risk of preeclampsia."<br>"The present study found no evidence to support that the extra vitamin D from a mandatory food fortification program resulted in a lower preeclampsia risk. Nevertheless, the observed seasonality pattern in preeclampsia incidence indicates that vitamin D from the sun may have a role in preeclampsia prevention, and such a possibility needs to be examined in further studies."

Danish study

		Description	
**Study Characteristics**	**Study ID**	#2798	
	**Author, date**	**Moos, 2020**	
	**Publication type** *(e.g. full report, abstract, letter)*	full reports	
	**Language of publication**	English	
	**Funding**	Furthermore, Knud og Edith Eriksens Mindefond and Københavns Kommunes uddannelseslegater (Georg og Emilie Petersen Legatfond) supported the conducting of this study.	
	**Conflict** *(notable conflicts of interest of study authors)*	The authors declare no conflict of interest.	
**Methods**	**Aim of study**	The objectives of this study were as follows:<br>(i) To investigate if season of birth and the concomitant production of vitamin D from sunlight was associated with the risk of developing coeliac disease (CD)<br>(ii) To investigate if individuals with fetal exposure to extra vitamin D from the mandatory vitamin D food fortification policy had a decreased risk of developing CD later in life compared to individuals with no fetal exposure<br>(iii) To examine if the risk of developing CD related to prenatal exposure to extra vitamin D from the fortification policy was dependent on season of birth and if risk reduction is potentially stronger for summer born compared to winter born children	
	**Study design** *(RCT/ CCT/ cohort/ controlled before-after/ interrupted time series)*	prospective, birth cohort study<br>semi-ecological study	
	**Unit of allocation** *(by individuals, cluster/ groups or body parts)*	groups	
	**Number of study arms**	2 (Exposed, Unexposed)	
	**Number of study centres**	not applicable	
	**Start date of study**	not applicable	
	**End date of study**	not applicable	
**Participants**	**Population description** *(from which study participants are drawn)*	„The exposed study population consisted of all children born in Denmark in the two-year period between 01 June 1983 and 31 May 1985 (these children were exposed to the vitamin D fortification policy prenatally) and all children in Denmark born in the two year period between 01 September 1986 and 31 August 1988 (these children were not exposed to vitamin D fortification policy prenatally)."	
	**Country where trial was performed**	Denmark	
	**Location/Setting**	-	
	**Inclusion criteria**	all children born in Denmark in the two-year period between 01 June 1983 and 31 May 1985 (these children were exposed to the vitamin D fortification policy prenatally) and all children in Denmark born in the two year period between 01 September 1986 and 31 August 1988 (these children were not exposed to vitamin D fortification policy prenatally).	
	**Exclusion criteria**	Individuals who died or emigrated	
	**Number of participants**	Number of contacted persons	not applicable
		Agreed to participate	not applicable
		Sample size<br>*(started the study)*	217249 (exposed 103606, nonexposed 113643)
		Lost to follow-up/withdrawals/drop outs	3699 dead (exposed 1811, nonexposed 1888)<br>6650 emigrated<br>(exposed 2939, nonexposed 3711)
		Number of participant analysed	-
		Sample size of total group	206900
		Sample size of each groups	Exposed: 98856<br>Not exposed: 108044
	**Sex** *(Male/Female; Boys/Girls)*	girls and boys	
	**Mean age (age range)**	not applicable	
	**Race/Ethnicity**	not defined	
	**Other relevant baseline data**	not mention	

	(e.g. BMI, health status, pregnant ect.)	
	**Subgroups measure**	-
	**Subgroups reported**	-
**Interventions**	**Intervention(s)**	In Denmark, margarine fortification with 1.25 µg vitamin D/100 g margarine was mandatory until 1st June 1985, when the fortification policy was terminated due to a political decision.
	**Comparator(s)**	children were not exposed to vitamin D fortification policy prenatally
	**Dosage** *(e.g recorded by FFQ)*	margarine fortification with 1.25 µg vitamin D/100 g margarine
	**Fortified oil** *(e.g type, name ect.)*	margarine
	**Duration of intervention**	two-year period between 01 June 1983 and 31 May 1985
	**Duration of follow-up**	"long follow-up period of 30 years"
	**Run-in period**	not applicable
	**Concomitant intervention**	During the study period margarine was also fortified with vitamin A, and the new evidence reveals that the vitamin A dose was increased by 25 % (from 20 to 25 IU/g) in 1962. There were also minor increases in 1971 from 25 to 26 IU/g and in 1985 from 26 to 28 IU/g. (Jensen, 2016)
	**Timing** *(e.g. frequency, duration of each episode)*	not applicable

		**Description**
**Outcomes**	**Primary outcomes defined by the authors**	CD (coeliac disease) diagnosis
	**Secondary outcomes defined by the authors**	
	**Confounder** *(uncontrolled, but not adjusted; list of variables authors adjusted variables; Methods used to control to confounders)*	sex of the offspring (man, woman), season of birth

		**Description**		
**Outcome 2.**	**Outcome name**	CD diagnosis (coeliac disease)		
	**Outcome definition** *(with diagnostic criteria if relevant)*	two or more records in the DNPR of ICD-8 code 269 and/or ICD-10 code K900		
	**Time point** *(specify from start or end of intervention) for example 3,6,9,12*	not applicable		
	**Unit of analysis** *(individuals, cluster/ groups or body parts)*	individuals		
	**Post-intervention or change from baseline?**	not applicable		
	**Unit of measurement**	%		
	**Results**	**Intervention group**		
		**Number of participants**	**Number of individuals with CD**	**%**
		98856	148	0.15
		**Comparison group**		
		**Number of participants**	**Number of individuals with CD**	**%**
		108044	199	0.18
	**Any other results reported** *(e.g. mean difference, CI, P value)*	"There was a higher number of women than men who developed CD, with the majority being diagnosed after the age of 15 years."<br>"Season of birth was significantly associated with a change in the odds of developing CD, particularly for subjects born in autumn (OR = 1.60, 95% CI 1.16; 2.21 p = 0.004) and summer (OR = 1.51, 95% CI 1.10; 2.09 p = 0.01) compared to subjects born in winter. The odds ratio for developing CD was 0.81 (95% CI 0.66; 1.00 p = 0.054), comparing those with fetal exposure to mandatory vitamin D fortification policy of margarine to those without after adjusting for gender and season of birth. There was no significant interaction between season of birth and the prenatal exposure to the mandatory vitamin D fortification policy on the odds of developing CD later in life (p = 0.56)."		

	**Conclusion**
**Conclusion**	Although this study did not find evidence to support the premise that prenatal exposure to small extra amounts of vitamin D from a mandatory food fortification policy lowered risk of developing CD, the small number of CD and observed association between season of birth and CD suggest that environmental exposure ought to be further explored.

<table>
<tr><td></td><td colspan="3">Description</td></tr>
<tr><td rowspan="7">Study Characteristics</td><td>Study ID</td><td colspan="2">#1064</td></tr>
<tr><td>Author, date</td><td colspan="2">Duus, 2021</td></tr>
<tr><td>Publication type (e.g. full report, abstract, letter)</td><td colspan="2">full reports</td></tr>
<tr><td>Language of publication</td><td colspan="2">English</td></tr>
<tr><td>Funding</td><td colspan="2">This study was a part of the D-tect study funded by the Danish Agency for Science, Technology, and Innovation, the Ministry of Science, Higher Education, under the instrument “Strategic Research Projects” (grant 0603-00453B) as a part of the D-tecting disease Project.
Furthermore, Knud og Edith Eriksens Mindefond and Københavns Kommunes uddannelseslegater (Georg og Emilie Petersen Legatfond) supported the conducting of this study.</td></tr>
<tr><td>Conflict
(notable conflicts of interest of study authors)</td><td colspan="2">The authors declare no conflict of interest.</td></tr>
<tr><td rowspan="7">Methods</td><td>Aim of study</td><td colspan="2">This study aimed to investigate if exposure to a small extra dose of vitamin D from fortified margarine during gestation and in early life would lower the risk of offspring Inflammatory bowel disease (IBD) later in life. We also explored whether individuals born in summer and autumn would have a higher risk of IBD and if the extra vitamin D from fortification prenatally would benefit, in particular, individuals born in summer and autumn.</td></tr>
<tr><td>Study design
(RCT/ CCT/ cohort/ controlled before-after/ interrupted time series)</td><td colspan="2">prospective, birth cohort study
“societal experiment”</td></tr>
<tr><td>Unit of allocation (by individuals, cluster/ groups or body parts)</td><td colspan="2">groups</td></tr>
<tr><td>Number of study arms</td><td colspan="2">2 (Exposed, Unexposed)</td></tr>
<tr><td>Number of study centres</td><td colspan="2">not applicable</td></tr>
<tr><td>Start date of study</td><td colspan="2">not applicable</td></tr>
<tr><td>End date of study</td><td colspan="2">not applicable</td></tr>
<tr><td rowspan="19">Participants</td><td>Population description
(from which study participants are drawn)</td><td colspan="2">two full-year birth cohorts from the Danish Medical Birth Registry immediately before and after the fortification policy’s termination.</td></tr>
<tr><td>Country where trial was performed</td><td colspan="2">Denmark</td></tr>
<tr><td>Location/Setting</td><td colspan="2">-</td></tr>
<tr><td>Inclusion criteria</td><td colspan="2">All individuals were selected from two full-year birth cohorts from the Danish Medical Birth Registry immediately before and after the fortification policy’s termination. Exposed individuals were born between 1st June 1983, and 31st May 1985; unexposed individuals were born between 1st September 1986, and 31st August 1988.</td></tr>
<tr><td>Exclusion criteria</td><td colspan="2">Individuals who died or emigrated</td></tr>
<tr><td rowspan="7">Number of participants</td><td>Number of contacted persons</td><td>not applicable</td></tr>
<tr><td>Agreed to participate</td><td>not applicable</td></tr>
<tr><td>Sample size
(started the study)</td><td>217249 (exposed 103606, nonexposed 113643)</td></tr>
<tr><td>Lost to follow-up/withdrawals/drop outs</td><td>3699 dead (exposed 1811, nonexposed 1888)
6650 emigrated
(exposed 2939, nonexposed 3711)</td></tr>
<tr><td>Number of participant analysed</td><td>-</td></tr>
<tr><td>Sample size of total group</td><td>206900</td></tr>
<tr><td>Sample size of each groups</td><td>Exposed: 98856
Not exposed: 108044</td></tr>
<tr><td>Sex
(Male/Female; Boys/Girls)</td><td colspan="2">girls and boys</td></tr>
<tr><td>Mean age
(age range)</td><td colspan="2">not applicable</td></tr>
<tr><td>Race/Ethnicity</td><td colspan="2">not defined</td></tr>
<tr><td>Other relevant baseline data
(e.g. BMI, health status, pregnant ect.)</td><td colspan="2">not mention</td></tr>
<tr><td>Subgroups measure</td><td colspan="2">-</td></tr>
<tr><td>Subgroups reported</td><td colspan="2">-</td></tr>
<tr><td rowspan="4">Interventions</td><td>Intervention(s)</td><td colspan="2">In Denmark, margarine fortification with 1.25 µg vitamin D/100 g margarine was mandatory until 1st June 1985, when the fortification policy was terminated due to a political decision.</td></tr>
<tr><td>Comparator(s)</td><td colspan="2">children were not exposed to vitamin D fortification policy prenatally</td></tr>
<tr><td>Dosage
(e.g recorded by FFQ)</td><td colspan="2">margarine fortification with 1.25 µg vitamin D/100 g margarine</td></tr>
<tr><td>Fortified oil
(e.g type, name ect.)</td><td colspan="2">margarine</td></tr>
</table>

	**Duration of intervention**	wo-year period between 01 June 1983 and 31 May 1985
	**Duration of follow-up**	followed for 30 years from the day of birth
	**Run-in period**	not applicable
	**Concomitant intervention**	During the study period margarine was also fortified with vitamin A, and the new evidence reveals that the vitamin A dose was increased by 25 % (from 20 to 25 IU/g) in 1962. There were also minor increases in 1971 from 25 to 26 IU/g and in 1985 from 26 to 28 IU/g. (Jensen, 2016)
	**Timing** *(e.g. frequency, duration of each episode)*	not applicable

**Outcomes**		**Description**
	**Primary outcomes defined by the authors**	IBD diagnosis
	**Secondary outcomes defined by the authors**	
	**Confounder** *(uncontrolled, but not adjusted; list of variables authors adjusted variables; Methods used to control to confounders)*	month of birth, and type of IBD (Crohn's disease, ulcerative colitis).

**Outcome 1**.		**Description**		
	**Outcome name**	IBD (two types of IBD: CD+ UC = Crohn's disease, ulcerative colitis)		
	**Outcome definition** *(with diagnostic criteria if relevant)*	Two or more records in the Danish National Patient Registry (DNPR) of ICD-8 codes 563.01, 563.02, 563.08, 563.09, 563.19, and 569.04 until the end of 1993 and, hereafter, ICD-10 codes K50 and K51.		
	**Time point** *(specify from start or end of intervention) for example 3,6,9,12*	not applicable		
	**Unit of analysis** *(individuals, cluster/ groups or body parts)*	individuals		
	**Post-intervention or change from baseline?**	not applicable		
	**Unit of measurement**	%		
	**Results**	**Intervention group Exposed**		
		**Number of participants**	**Number of individuals with IBD**	**%**
		100667	875	0.87
		**Comparison group Unexposed**		
		**Number of participants**	**Number of individuals with IBD**	**%**
		109932	1102	1
	**Any other results reported** *(e.g. mean difference, CI, P value)*	13% lower odds for incident IBD among those exposed prenatally to extra vitamin D from fortified margarine compared to those not exposed<br>"At the end of the 30-year follow-up, 47.8% among those without IBD had been exposed to extra vitamin D from fortification, compared to 44.3% of those diagnosed with IBD (Table 1). More women were diagnosed with IBD (56.9%) than men. No differences in IBD incidence were seen for season or month of birth." „No overall association with month of birth was found (p = 0.18)." „The overall likelihood ratio test showed no interaction between being exposed to the fortification policy and season of birth (p = 0.28). Among the autumn-born, extra vitamin D from the fortification policy reduced the odds of developing IBD (OR = 0.75 (95% CI: 0.63; 0.89)."		

**Conclusion**	**Conclusion**
	"In conclusion, our study suggests that the Danish vitamin D fortification policy, providing a small extra dose of vitamin D during gestation, lowered the odds of developing IBD over the subsequent 30 years. It was indicated that an insufficient intake of vitamin D during gestation might contribute to IBD development."

		Description
Study Characteristics	**Study ID**	#2075
	**Author, date**	**Keller, 2018**
	**Publication type** *(e.g. full report, abstract, letter)*	full text report and registry (NCT03330301)
	**Language of publication**	English
	**Funding**	The study was part of the 4-year project "D-tect" funded by the Programme Commission on Health, Food, and Welfare under the Danish Council for Strategic research (grant number 0603-00453B). The Lundbeck Foundation (grant number R170-2014-643). The Danish Diabetes Academy supported by the Novo Nordisk Foundation. The funders had no role in the design of the study and collection, analysis, and interpretation of data and in writing the manuscript.
	**Conflict** *(notable conflicts of interest of study authors)*	The authors declare no conflict of interest.
Methods	**Aim of study**	„The primary aim of this study was to assess whether exposure during fetal life to extra vitamin D from food fortification was associated with a decreased risk of subsequently developing gestational diabetes mellitus (GDM). Furthermore, we examined whether the effect of the margarine fortification differed by women's season of birth."
	**Study design** *(RCT/ CCT/ cohort/ controlled before-after/ interrupted time series)*	prospective, observational, birth cohort study, semi-ecological study
	**Unit of allocation** *(by individuals, cluster/ groups or body parts)*	group<br>„All women born in Denmark around the fortification termination, between June 1983 and August 1988 and who later gave birth were included in this study."
	**Number of study arms**	2
	**Number of study centres**	not applicable
	**Start date of study**	not applicable
	**End date of study**	not abblicable
Participants	**Population description** *(from which study participants are drawn)*	„All women born in Denmark around the fortification termination, between June 1983 and August 1988 and who later gave birth were included in this study."
	**Country where trial was performed**	Denmark
	**Location/Setting**	-
	**Inclusion criteria**	"The main analyses were restricted to nulliparous women who gave birth for the first time to a singleton infant between January 2004 and February 2016 after at least 22 weeks of gestation.<br>In addition, women had to be between 20.6 and 27.5 years of age at time of delivery, to ensure same length of follow-up in both exposure groups"<br>"The inclusion of all women from entire birth cohorts of the whole Danish population was made possible by the complete registration of every citizen via a CPR number into the Danish national health registers."
	**Exclusion criteria**	• missing information on the following variables: age at delivery, whether it was a singleton or multiple births and offspring's gestational age at delivery.<br>• women who were born during the 15-month's wash-out period (from June 1985 to August 1986) were excluded.<br>• Women with a pre-pregnancy BMI ≤15 or ≥ 50 kg/m2 were also excluded.<br>• own birth weight was registered as 0 g<br>• women < 20.6 and > 27.5 years old or missing.<br>• gestational weeks was < 22 weeks
	**Number of participants**	Number of identified persons: 162397<br>Agreed to participate: not applicable<br>Sample size *(started the study)*: 112311<br>Lost to follow-up/withdrawals/drop outs: excluded:<br>27003: women born during the 15 months' wash-out period from June 1985 to August 1986<br>39926: Women < 20.6 and > 27.5 years old or missing<br>16511: gestational weeks was < 22 weeks or missing, pre-pregnancy BMI was < 15 kg/m2 or

			> 50 kg/m2, women's own birth weight was 0 g, multiple births, stillbirths, multiparous women
		Number of participant analysed	28871
		Sample size of total group	28871
		Sample size of each groups	Exposed women: 14016<br>Unexposed women: 14855
	**Sex**<br>*(Male/Female; Boys/Girls)*	Female	
	**Median age (age range)**	25.2 (20.6 - 27.5 years)	
	**Race/Ethnicity**	not defined	
	**Other relevant baseline data**<br>*(e.g. BMI, health status, pregnant ect.)*	Unexposed women were more likely to be former smokers and non-smokers compared to exposed women (5.6% vs. 5.2% and 81.3% vs.79.1%, respectively). Exposed women more often gave birth after 42 weeks of gestation (6.0% vs. 3.4%), and slightly more often had a pre-pregnancy BMI between 18.5 and 25 kg/m2 than unexposed women (60.7% vs. 59.6%) (p = 0.01). There was no difference in the distribution of the women's season of birth between the two exposure groups (p = 0.8). Women born between 1983 and 1985 were exposed to more hours of bright sunshine during gestation than women born after the vitamin D fortification termination, median (5; 95 percentiles) 1108 (752;1311) and 1004 (772;1320) hours respectively (p < 0.0001).	
	**Subgroups measure**	-	
	**Subgroups reported**	-	

**Interventions**	**Intervention(s)**	„In Denmark, fortification of margarine with vitamin A and D started in 1937, and fortification with vitamin D was stopped by law on the 1st June 1985. Between 1962 and 1985, margarine was fortified with 1.25 µg vitamin D/100 g of margarine, representing up to 29% of total dietary vitamin D intake (average 13%)."
	**Comparator(s)**	"the unexposed group was truly unexposed, a 15-month wash-out period was introduced, from June 1985 to September 1986, accounting for the 9 months of pregnancy and an additional 6 months to ensure vitamin D fortified margarine was no longer available on the Danish market or households. Thus, women born between September 1986 and August 1988 were considered to be unexposed."
	**Dosage**<br>*(e.g recorded by FFQ)*	„Between 1962 and 1985, margarine was fortified with 1.25 µg vitamin D/100 g of margarine, representing up to 29% of total dietary vitamin D intake (average 13%)."
	**Fortified oil**<br>*(e.g type, name ect.)*	margarine
	**Duration of intervention**	between 01 June 1983 and 31 May 1985
	**Duration of follow-up**	-
	**Run-in period**	not applicable
	**Concomitant intervention**	During the study period margarine was also fortified with vitamin A, and the new evidence reveals that the vitamin A dose was increased by 25 % (from 20 to 25 IU/g) in 1962. There were also minor increases in 1971 from 25 to 26 IU/g and in 1985 from 26 to 28 IU/g. (Jensen, 2016)
	**Timing**<br>*(e.g. frequency, duration of each episode)*	not applicable

**Outcomes**		**Description**
	**Primary outcomes defined by the authors**	incidence of gestational diabetes mellitus
	**Secondary outcomes defined by the authors**	-
	**Confounder** *(uncontrolled, but not adjusted; list of variables authors adjusted variables; Methods used to control to confounders)*	smoking status during pregnancy, age at time of delivery, birth weight, pre-pregnancy BMI, singleton and multiple births, offspring gender, gestational age and parity

**Outcome 1.**		**Description**
	**Outcome name**	incidence of gestational diabetes mellitus (GDM)
	**Outcome definition** *(with diagnostic criteria if relevant)*	"A 75 g 2-h oral glucose tolerance test (OGTT) is performed at 27–30 weeks and also in early pregnancy (14–20 weeks). The diagnostic criteria for GDM are a 2-h capillary blood or venous plasma glucose ≥9 mmol/L."<br>To ensure GDM diagnosis was harmonized all over Denmark, GDM diagnoses from 1st January 2004 and onwards were included in this study.
	**Time point**<br>*(specify from start or end of intervention) for example 3,6,9,12*	not applicable
	**Unit of analysis**<br>*(individuals, cluster/ groups or body parts)*	individuals
	**Post-intervention or change from baseline?**	not applicable

	**Unit of measurement**	%		
	**Results**	**Intervention group**		
		**Number of participants**	**Number of women with GDM**	**%**
		14016	297	2.1
		**Comparison group**		
		**Number of participants**	**Number of women with GDM**	**%**
		14855	361	2.4
	**Any other results reported** *(e.g. mean difference, CI, P value)*	Difference between exposed and unexposed group (p-value): 0.08<br>"Women prenatally exposed to the vitamin D fortification tended to have a lower risk of developing GDM than unexposed women (Crude model (no adjustment): OR 0.87, 95%CI 0.74,1.02, p = 0.08; Model 1 (adjustment for women's season of birth): OR 0.87, 95%CI 0.74,1.02, P = 0.08)"<br>Women's season of birth, exposed women born in spring (winter pregnancies) had a lower risk of developing GDM compared to those unexposed to extra vitamin D (OR 0.68, 95%CI 0.50,0.94, p = 0.02)<br>There was a higher GDM incidence with higher age at delivery (p = 0.004), and both women's (p = 0.04) and offspring's years of birth (p < 0.0001).		

	**Conclusion**
**Conclusion**	„This study suggests that prenatal exposure to extra vitamin D from mandatory fortification may lower the risk of developing gestational diabetes among spring-born women, eg. from winter pregnancies, when the small extra amount of vitamin D from fortification seemed particularly beneficial. Our results may have public health relevance as they demonstrate that mothers consuming extra vitamin D from food fortification had daughters who were at lower subsequent risk of developing GDM."

		Description
Study Characteristics	**Study ID**	#4133
	**Author, date**	**Thorsteinsdottir, 2019**
	**Publication type** *(e.g. full report, abstract, letter)*	full text report
	**Language of publication**	English
	**Funding**	The research was funded by the Danish Agency for Science, Technology and Innovation, the Ministry of Science, Higher Education (grant 0603-00453B)
	**Conflict** *(notable conflicts of interest of study authors)*	The authors declare no conflict of interest.
Methods	**Aim of study**	"The objective of the present study was to examine if children born to women exposed to the margarine fortification policy with a small dose of extra vitamin D during pregnancy had a reduced risk of developing asthma until age 9 years compared to children whose mothers were unexposed to the fortification policy during pregnancy. Furthermore, this study examined whether the association between exposure and asthma risk varied by age, sex, and month of birth."
	**Study design** *(RCT/ CCT/ cohort/ controlled before-after/ interrupted time series)*	prospective, observational, birth cohort study,
	**Unit of allocation** *(by individuals, cluster/ groups or body parts)*	groups ("All children born alive in Denmark from June 1983–May 1985 and from September 1986–August 1988 were identified using the Danish Civil Registration System (CRS) and included in this study.")
	**Number of study arms**	2
	**Number of study centres**	not applicable
	**Start date of study**	not applicable
	**End date of study**	not abblicable
Participants	**Population description** *(from which study participants are drawn)*	All children born alive in Denmark from June 1983–May 1985 and from September 1986–August 1988 were identified using the Danish Civil Registration System (CRS) and included in this study
	**Country where trial was performed**	Denmark
	**Location/Setting**	-
	**Inclusion criteria**	„Briefly, all individuals born in Denmark during the two years before the termination of the vitamin D policy in 1985 were considered as exposed to vitamin D fortification during prenatal life, and all individuals born during the two years after the termination (excluding a wash-out period- May 1985-September 1986), were considered unexposed to vitamin D fortification."<br><br>„All children born alive in Denmark from June 1983–May 1985 and from September 1986–August 1988 were identified using the Danish Civil Registration System (CRS) and included in this study."
	**Exclusion criteria**	• births during washout period<br>• dead or lost, emigrated
	**Number of participants**	Number of identified persons: 293014<br>Agreed to participate: not applicable<br>Sample size *(started the study)*:<br>Lost to follow-up/withdrawals/drop outs: Dead or lost to follow-up before birth or on the day of birth n=410<br>Number of participant analysed: -<br>Sample size of total group: 222247<br>Sample size of each groups: Exosed: 106347 unexposed: 115900
	**Sex** *(Male/Female; Boys/Girls)*	boys and girls
	**Median age (age range)**	not defined
	**Race/Ethnicity**	not defined
	**Other relevant baseline data** *(e.g. BMI, health status, pregnant ect.)*	not define
	**Subgroups measure**	-
	**Subgroups reported**	-

**Interventions**	**Intervention(s)**	"In Denmark, between 1937 and 1985, it was mandatory to fortify margarine with 1.25 µg vitamin D per 100 g. The fortification accounted for on average 13% (3–29%) of total vitamin D intake from food in the Danish population."
	**Comparator(s)**	unexposed group:<br>All children born in Denmark from September 1986 to August 1988.
	**Dosage**<br>*(e.g recorded by FFQ)*	„Between 1962 and 1985, margarine was fortified with 1.25 µg vitamin D/100 g of margarine, representing up to 29% of total dietary vitamin D intake (average 13%)."
	**Fortified oil**<br>*(e.g type, name ect.)*	margarine
	**Duration of intervention**	between 01 June 1983 and 31 May 1985
	**Duration of follow-up**	-
	**Run-in period**	not applicable
	**Concomitant intervention**	During the study period margarine was also fortified with vitamin A, and the new evidence reveals that the vitamin A dose was increased by 25 % (from 20 to 25 IU/g) in 1962. There were also minor increases in 1971 from 25 to 26 IU/g and in 1985 from 26 to 28 IU/g. (Jensen, 2016)
	**Timing**<br>*(e.g. frequency, duration of each episode)*	not applicable

**Outcomes**		**Description**
	**Primary outcomes defined by the authors**	asthma diagnoses from birth to the age of 9 years
	**Secondary outcomes defined by the authors**	-
	**Confounder** *(uncontrolled, but not adjusted; list of variables authors adjusted variables; Methods used to control to confounders)*	sex and month of birth

**Outcome 2.**		**Description**		
	**Outcome name**	incidence of asthma diagnoses from birth to the age of 9 years		
	**Outcome definition** *(with diagnostic criteria if relevant)*	Asthma was defined based on ICD-8 codes 493.00, 493.01, 493.08 and 493.09; and from 1994 onwards on ICD-10 codes DJ45, DJ45.0, DJ45.1, DJ45.8, DJ45.9, DJ46.0. The registry diagnoses of asthma have been previously validated against medical records.		
	**Time point**<br>*(specify from start or end of intervention) for example 3,6,9,12*	not applicable		
	**Unit of analysis**<br>*(individuals, cluster/ groups or body parts)*	individuals		
	**Post-intervention or change from baseline?**	not applicable		
	**Unit of measurement**	%		
	**Results**	**Intervention group**		
		**Number of participants**	**Number of children with asthma**	**%**
		106347	1427	1.34
		**Comparison group**		
		**Number of participants**	**Number of children with asthma**	**%**
		115900	1613	1.39
	**Girls Results**	**Intervention group**		
		**Number of participants**	**Number of children with asthma**	**%**
		51724	515	1
		**Comparison group**		
		**Number of participants**	**Number of children with asthma**	**%**
		56036	562	1
	**Boys Results**	**Intervention group**		
		**Number of participants**	**Number of children with asthma**	**%**
		54623	912	1.67
		**Comparison group**		
		**Number of participants**	**Number of children with asthma**	**%**
		59864	1051	65.2
	**Any other results reported**<br>*(e.g. mean difference, CI, P value)*	**Whole population:**		

Hazard ratio: 0.96; (95% CI): (0.9-1.03); Adjusted hazard ratio: 0.96; (95% CI): (0.9-1.04); p for interaction with month of birth: 0.28
Exposed:
Time at risk: 938797 years; Rate per 100,000 years at risk: 152
Unexposed:
Time at risk: 102211 years; Rate per 100,000 years at risk: 157.8

0-3 years old population:
Hazard ratio: 0.86; (95% CI): (0.75-0.98); Adjusted hazard ratio: 0.86; (95% CI): (0.75-0.98); p for interaction with month of birth: 0.63
Exposed:
Admissions: 393
Time at risk: 315879 years; Rate per 100,000 years at risk: 124.4
Unexposed:
Admissions: 498
Time at risk: 343928 years; Rate per 100,000 years at risk: 144.8

4-6 years old population:
Hazard ratio: 0.95; (95% CI): (0.85-1.06); Adjusted hazard ratio: 0.95; (95% CI): (0.85-1.06); p for interaction with month of birth: 0.02
Exposed:
Admissions: 596
Time at risk: 312640 years; Rate per 100,000 years at risk: 190.6
Unexposed:
Admissions: 682
Time at risk: 340339 years; Rate per 100,000 years at risk: 200.4

7-9 years old population:
Hazard ratio: 1.1; (95% CI): (0.96-1.26); Adjusted hazard ratio: 1.1; (95% CI): (0.96-1.26); p for interaction with month of birth: 0.78
Exposed:
Admissions: 438
Time at risk: 310278 years; Rate per 100,000 years at risk: 141.2
Unexposed:
Admissions: 433
Time at risk: 337843 years; Rate per 100,000 years at risk: 128.2

Girls:
Hazard ratio: 0.99; (95% CI): (0.88-1.12); Adjusted hazard ratio: 0.99; (95% CI): (0.88-1.12); p for interaction with month of birth: 0.28
Exposed:
Time at risk: 457866 years; Rate per 100,000 years at risk: 112.5
Unexposed:
Time at risk: 496107 years; Rate per 100,000 years at risk: 113.3

0-3 years old girl population:
Hazard ratio: 1.03; (95% CI): (0.82-1.3); Adjusted hazard ratio: 1.03; (95% CI): (0.82-1.3); p for interaction with month of birth: 0.98
Exposed:
Admissions: 146
Time at risk: 153849 years; Rate per 100,000 years at risk: 94.9
Unexposed:
Admissions: 153
Time at risk: 166624 years; Rate per 100,000 years at risk: 91.8

4-6 years old girl population:
Hazard ratio: 0.97; (95% CI): (0.81-1.16); Adjusted hazard ratio: 0.97; (95% CI): (0.81-1.16); p for interaction with month of birth: 0.14
Exposed:
Admissions: 221
Time at risk: 152494 years; Rate per 100,000 years at risk: 144.9
Unexposed:
Admissions: 248
Time at risk: 165223 years; Rate per 100,000 years at risk: 150.1

7-9 years old girl population:
Hazard ratio: 1.0; (95% CI): (0.8-1.25); Adjusted hazard ratio: 1.0; (95% CI): (0.8-1.24); p for interaction with month of birth: 0.41
Exposed:
Admissions: 148
Time at risk: 151523 years; Rate per 100,000 years at risk: 97.7
Unexposed:
Admissions: 161
Time at risk: 164259 years; Rate per 100,000 years at risk: 98

Boys:
Hazard ratio: 0.95; (95% CI): (0.87-1.04); Adjusted hazard ratio: 0.95; (95% CI): (0.87-1.04); p for interaction with month of birth: 0.5
Exposed:
Time at risk: 480931 years; Rate per 100,000 years at risk: 189.6
Unexposed:
Time at risk: 526003 years; Rate per 100,000 years at risk: 199.8

0-3 years old boy population:

	Hazard ratio: 0.78; (95% CI): (0.67-0.92); Adjusted hazard ratio: 0.78; (95% CI): (0.67-0.92); p for interaction with month of birth: 0.24<br>**Exposed:**<br>Admissions: 247<br>Time at risk: 177304 years; Rate per 100,000 years at risk: 194.6<br>**Unexposed:**<br>Admissions: 345<br>Time at risk: 162030 years; Rate per 100,000 years at risk: 152.4<br>**4-6 years old boy population:**<br>Hazard ratio: 0.94; (95% CI): (0.82-1.08); Adjusted hazard ratio: 0.95; (95% CI): (0.82-1.09); p for interaction with month of birth: 0.1<br>**Exposed:**<br>Admissions: 375<br>Time at risk: 175116 years; Rate per 100,000 years at risk: 247.8<br>**Unexposed:**<br>Admissions: 434<br>Time at risk: 160146 years; Rate per 100,000 years at risk: 234.2<br>**7-9 years old girl population:**<br>Hazard ratio: 1.17; (95% CI): (0.99-1.38); Adjusted hazard ratio: 1.16; (95% CI): (0.99-1.37); p for interaction with month of birth: 0.71<br>**Exposed:**<br>Admissions: 290<br>Time at risk: 173583 years; Rate per 100,000 years at risk: 156.7<br>**Unexposed:**<br>Admissions: 272<br>Time at risk: 158755 years; Rate per 100,000 years at risk: 182.7

	**Conclusion**
**Conclusion**	"Our study, based on the societal experiment concerning margarine fortification with vitamin D in Denmark, suggests that prenatal exposure to a small dose of extra vitamin D from fortification may be associated with a lower risk of childhood asthma among boys aged 0–3 years, but not among older children or the youngest girls. Asthma phenotypes with very early onset that have different vitamin D sensitivity and/or sex differences in lung development or immune responses may explain our findings. However, residual confounding effects due to the semi-ecological design of the study cannot be ruled out."

		Description	
**Study Characteristics**	**Study ID**	#1859	
	**Author, date**	***Jacobsen, 2015***, Jacobsen, 2016	
	**Publication type** *(e.g. full report, abstract, letter)*	full text	
	**Language of publication**	English	
	**Funding**	The study is a part of the 4 years project "D-tect" funded by the Programme Commission on Health, Food, and Welfare under the Danish Council for Strategic research (grant number 0603-00453B).<br>The funders had no role in study design, data collection and analysis, decision to publish, or preparation of the manuscript.	
	**Conflict** *(notable conflicts of interest of study authors)*	**Jacobsen**: The authors have declared that no competing interests exist.	
**Methods**	**Aim of study**	**Jacobsen (2016)**:<br>The aim of this study was to assess whether gestational and early infancy exposure to small extra doses of vitamin D coming from fortified margarine influenced the risk of developing T1D later in life. We hypothesised that T1D risk will be lower among individuals born in Denmark during the period of obligatory margarine fortification—and therefore exposed to vitamin D fortified food during gestation or infancy—compared to individuals born in years when margarine was not fortified—and consequently unexposed to extra vitamin D coming from fortified margarine at any period of early life.<br>**Jacobsen (2015)**:<br>The objective of the study was to assess whether gestational and early infancy exposure to low dose vitamin D from a mandatory margarine fortification programme in Denmark influenced the risk of developing type 1 diabetes (T1D) before age of 15 years.	
	**Study design** *(RCT/ CCT/ cohort/ controlled before-after/ interrupted time series)*	prospective, birth cohort	
	**Unit of allocation** *(by individuals, cluster/ groups or body parts)*	birth cohort	
	**Number of study arms**	2	
	**Number of study centres**	not applicable	
	**Start date of study**	not applicable	
	**End date of study**	not applicable	
**Participants**	**Population description** *(from which study participants are drawn)*	All individuals born in Denmark in 1983–1988.	
	**Country where trial was performed**	Denmark	
	**Location/Setting**	-	
	**Number of study centers**	not applicable	
	**Inclusion criteria**	All individuals born alive in Denmark from 1 January 1983 to 31 December 1988 were included in the study.	
	**Exclusion criteria**	not define	
	**Number of participants**	Number of contacted persons	not applicable
		Agreed to participate	not applicable
		Sample size *(started the study)*	not applicable
		Lost to follow-up/withdrawals/drop outs	not applicable
		Number of participant analysed	not defined
		Sample size of total group	331,623
		Sample size of each groups	**Jacobsen**:<br>Exposure: 127207<br>Washout: 69667<br>Non-exposure: 134749
	**Sex** *(Male/Female; Boys/Girls)*	boys and girls	
	**Mean age (age range)**	**Jacobsen**: 0-15 years	
	**Race/Ethnicity**	not define	
	**Other relevant baseline data** *(e.g. BMI, health status, pregnant ect.)*	not define	
	**Subgroups measure**	not define	
	**Subgroups reported**	subgroups by sex and age groups: until 15 years, 0-4 years, 5-9 years, 10-15 years	

		birth period in relation to exposure to vitamin D fortification during gestation and birth period in relation to exposure to vitamin D fortification during first year of postnatal life
**Interventions**	**Intervention(s)**	„The fact that until 1 June 1985 it was mandatory in Denmark to fortify all margarine with vitamin D. Margarine was fortified with 1·25 µg/100 g and approximately 13 % (3–29 %) of all dietary vitamin D is estimated to have come from the fortified margarine."
	**Comparator(s)**	not exposed period
	**Dosage** *(e.g recorded by FFQ)*	margarine fortification with 1.25 µg vitamin D/100 g margarine
	**Fortified oil** *(e.g type, name ect.)*	margarine
	**Duration of intervention**	January 1, 1983 to May 31, 1985
	**Duration of follow-up**	until age 15 years
	**Run-in period**	5 mounths
	**Concomitant intervention**	„During the study period margarine was also fortified with vitamin A, and the new evidence reveals that the vitamin A dose was increased by 25 % (from 20 to 25 IU/g) in 1962. There were also minor increases in 1971 from 25 to 26 IU/g and in 1985 from 26 to 28 IU/g" (Jensen 2016)
	**Timing** *(e.g. frequency, duration of each episode)*	not applicable

**Outcomes**		**Description**
	**Primary outcomes defined by the authors**	**Jacobsen:** Incidence of type 1 diabetes mellitus (before age of 15 years)
	**Secondary outcomes defined by the authors**	-
	**Confounder** *(uncontrolled, but not adjusted; list of variables authors adjusted variables; Methods used to control to confounders)*	sunshine hours in Copenhagen

**Outcome 1.**		**Description**		
	**Outcome name**	Type 1 diabetes mellitus (T1D) diagnosis		
	**Outcome definition** *(with diagnostic criteria if relevant)*	defined as date of the first insulin injection		
	**Time point** *(specify from start or end of intervention) for example 3,6,9,12*	not applicable		
	**Unit of analysis** *(individuals, cluster/ groups or body parts)*	individuals		
	**Post-intervention or change from baseline?**	not applicable		
	**Unit of measurement**	%		
	**Vitamin D fortification during gestation Results**	**Intervention group (Exposure)**		
		**Number of participants**	**Number of children with T1D**	**%**
		127207	298	0,23
		**Comparison group (Non-exposure)**		
		**Number of participants**	**Number of children with T1D**	**%**
		134749	404	0,3
	**Vitamin D fortification during gestation Male Results**	**Intervention group (Exposure)**		
		**Number of participants**	**Number of children with T1D**	**%**
		65266	157	0,24
		**Comparison group (Non-exposure)**		
		**Number of participants**	**Number of children with T1D**	**%**
		69436	224	0,3
	**Vitamin D fortification during gestation Female Results**	**Intervention group (Exposure)**		
		**Number of participants**	**Number of children with T1D**	**%**
		61941	141	0,23
		**Comparison group (Non-exposure)**		
		**Number of participants**	**Number of children with T1D**	**%**
		65313	180	0,28
		**Intervention group (Exposure)**		

**Vitamin D fortification during gestation<br><br>0-4 years group<br>Results**	**Number of participants**	**Number of children with T1D**	**%**
	127207	32	0,02
	**Comparison group (Non-exposure)**		
	**Number of participants**	**Number of children with T1D**	**%**
	134749	54	0,04
**Vitamin D fortification during gestation<br><br>0-4 years group<br>Male<br><br>Results**	**Intervention group (Exposure)**		
	**Number of participants**	**Number of children with T1D**	**%**
	65266	21	0,03
	**Comparison group (Non-exposure)**		
	**Number of participants**	**Number of children with T1D**	**%**
	69436	35	0,05
**Vitamin D fortification during gestation<br><br>0-4 years group<br>Female<br><br>Results**	**Intervention group (Exposure)**		
	**Number of participants**	**Number of children with T1D**	**%**
	61941	11	0,02
	**Comparison group (Non-exposure)**		
	**Number of participants**	**Number of children with T1D**	**%**
	65313	19	0,03
**Vitamin D fortification during gestation<br><br>5-9 years group<br>Results**	**Intervention group (Exposure)**		
	**Number of participants**	**Number of children with T1D**	**%**
	127207	83	0,07
	**Comparison group (Non-exposure)**		
	**Number of participants**	**Number of children with T1D**	**%**
	134749	131	0,1
**Vitamin D fortification during gestation<br><br>5-9 years group<br>Male<br>Results**	**Intervention group (Exposure)**		
	**Number of participants**	**Number of children with T1D**	**%**
	65266	38	0,06
	**Comparison group (Non-exposure)**		
	**Number of participants**	**Number of children with T1D**	**%**
	69436	75	0,11
**Vitamin D fortification during gestation<br><br>5-9 years group<br>Female<br>Results**	**Intervention group (Exposure)**		
	**Number of participants**	**Number of children with T1D**	**%**
	61941	45	0,07
	**Comparison group (Non-exposure)**		
	**Number of participants**	**Number of children with T1D**	**%**
	65313	56	0,09
**Vitamin D fortification during gestation<br><br>10-15 years group<br>Results**	**Intervention group (Exposure)**		
	**Number of participants**	**Number of children with T1D**	**%**
	127207	183	0,14
	**Comparison group (Non-exposure)**		
	**Number of participants**	**Number of children with T1D**	**%**
	134749	219	0,16
**Vitamin D fortification during gestation<br><br>10-15 years group<br>Male<br>Results**	**Intervention group (Exposure)**		
	**Number of participants**	**Number of children with T1D**	**%**
	65266	98	0,15
	**Comparison group (Non-exposure)**		
	**Number of participants**	**Number of children with T1D**	**%**
	69436	114	0,16
**Vitamin D fortification during gestation<br><br>10-15 years group<br>Female<br>Results**	**Intervention group (Exposure)**		
	**Number of participants**	**Number of children with T1D**	**%**
	61941	85	0,14
	**Comparison group (Non-exposure)**		
	**Number of participants**	**Number of children with T1D**	**%**
	65313	105	0,16
	**Intervention group (Exposure)**		

	Vitamin D fortification during first year of postnatal life<br><br>Results	**Number of participants**	**Number of children with T1D**	**%**
		73986	156	0,23
		**Comparison group (Non-exposure)**		
		**Number of participants**	**Number of children with T1D**	**%**
		134749	404	0,3
	Vitamin D fortification during first year of postnatal life<br><br>Male<br>Results	**Intervention group (Exposure)**		
		**Number of participants**	**Number of children with T1D**	**%**
		37977	82	0,22
		**Comparison group (Non-exposure)**		
		**Number of participants**	**Number of children with T1D**	**%**
		69436	224	0,32
	Vitamin D fortification during first year of postnatal life<br><br>Female<br>Results	**Intervention group (Exposure)**		
		**Number of participants**	**Number of children with T1D**	**%**
		36009	74	0,21
		**Comparison group (Non-exposure)**		
		**Number of participants**	**Number of children with T1D**	**%**
		65313	180	0,28
	Vitamin D fortification during first year of postnatal life<br><br>0-4 years<br><br>Results	**Intervention group (Exposure)**		
		**Number of participants**	**Number of children with T1D**	**%**
		73986	15	0,02
		**Comparison group (Non-exposure)**		
		**Number of participants**	**Number of children with T1D**	**%**
		134749	54	0,04
	Vitamin D fortification during first year of postnatal life<br>0-4 years<br><br>Male<br>Results	**Intervention group (Exposure)**		
		**Number of participants**	**Number of children with T1D**	**%**
		37977	7	0,02
		**Comparison group (Non-exposure)**		
		**Number of participants**	**Number of children with T1D**	**%**
		69436	35	0,05
	Vitamin D fortification during first year of postnatal life<br>0-4 years<br><br>Female<br>Results	**Intervention group (Exposure)**		
		**Number of participants**	**Number of children with T1D**	**%**
		36009	8	0,02
		**Comparison group (Non-exposure)**		
		**Number of participants**	**Number of children with T1D**	**%**
		65313	19	0,03
	Vitamin D fortification during first year of postnatal life<br><br>5-9 years<br><br>Results	**Intervention group (Exposure)**		
		**Number of participants**	**Number of children with T1D**	**%**
		73986	49	0,07
		**Comparison group (Non-exposure)**		
		**Number of participants**	**Number of children with T1D**	**%**
		134749	131	0,1
	Vitamin D fortification during first year of postnatal life<br><br>5-9 years<br>Male<br>Results	**Intervention group (Exposure)**		
		**Number of participants**	**Number of children with T1D**	**%**
		37977	19	0,05
		**Comparison group (Non-exposure)**		
		**Number of participants**	**Number of children with T1D**	**%**
		69436	75	0,11
	Vitamin D fortification during first year of postnatal life<br><br>5-9 years<br>Female<br>Results	**Intervention group (Exposure)**		
		**Number of participants**	**Number of children with T1D**	**%**
		36009	30	0,08
		**Comparison group (Non-exposure)**		
		**Number of participants**	**Number of children with T1D**	**%**
		65313	56	0,09
		**Intervention group (Exposure)**		

	Vitamin D fortification during first year of postnatal life<br><br>**10-15 years**<br><br>**Results**	**Number of participants**	**Number of children with T1D**	**%**
		73986	92	0,14
		**Comparison group (Non-exposure)**		
		**Number of participants**	**Number of children with T1D**	**%**
		134749	219	0,16
	**Vitamin D fortification during first year of postnatal life**<br><br>**10-15 years**<br>**Male**<br>**Results**	**Intervention group (Exposure)**		
		**Number of participants**	**Number of children with T1D**	**%**
		37977	56	0,15
		**Comparison group (Non-exposure)**		
		**Number of participants**	**Number of children with T1D**	**%**
		69436	114	0,16
	**Vitamin D fortification during first year of postnatal life**<br><br>**10-15 years**<br>**Female**<br>**Results**	**Intervention group (Exposure)**		
		**Number of participants**	**Number of children with T1D**	**%**
		36009	36	0,1
		**Comparison group (Non-exposure)**		
		**Number of participants**	**Number of children with T1D**	**%**
		65313	115	0,18
	**Any other results reported**<br>*(e.g. mean difference, CI, P value)*	The beta coefficients (95% CI), or slopes, for linear increase in the risk of developing T1D until age 15 years, after adjustment for sex, was 0.007 (0.003/0.0,010), p<0.001, log Hazard Ratio (HR) per one month of birth.<br><br>Erratum: „We found that after the adjustment for pregnancy sunshine hours, the seasonality of birth in T1D cases was now absent in both groups. Indeed, when comparing boys born in spring vs. boys born in autumn in the exposed group (i.e. born during a two-year-period before the fortification cancellation) the Hazard ratio (HR; 95% CI) for developing T1D before age 15 years was 0.78 (0.39–1.59). Similarly, HR; 95% CI in the unexposed group (i.e. born during a two-year-period after the fortification cancelation) was 0.93 (0.49–1.77). For boys who developed T1D at age 5–9 years, the respective results were: 2.10 (0.56–7.87) for the exposed and 2.80 (0.81–9.60) for unexposed. We also found that there were more sunshine hours during the pregnancy of the mothers to boys exposed to fortification compared to the mothers of unexposed boys: mean difference (SD) being 38 (0.8) hours.”		

**Conclusion**	**Conclusion**
	„We did not find that gestational or early infancy exposure to small extra doses of vitamin D coming from the Danish mandatory margarine fortification programme influenced the risk of developing T1D later in life.”

		Description	
**Study Characteristics**	**Study ID**	#1523	
	**Author, date**	**Händel, 2017**	
	**Publication type** *(e.g. full report, abstract, letter)*	full text	
	**Language of publication**	English	
	**Funding**	This study was supported by the Danish Council for Strategic Research (11-116213); and the University of Southern Denmark	
	**Conflict** *(notable conflicts of interest of study authors)*	C. C. has received consultancy, lecture fees and honoraria from Alliance for Better Bone Health, Amgen, Eli Lilly, GSK, Medtronic, Merck, Novartis, Pfizer, Roche, Servier, Takeda and Union Chimique Belge (UCB). B. A. conducts epidemiological studies through research contracts between his institution and Novartis and UCB Pharma. All other authors have nothing to disclose.	
**Methods**	**Aim of study**	"We hypothesised that individuals born during the last 2 years of the mandatory vitamin D fortification had a reduced risk of sustaining fractures of the forearm, wrist or scaphoid bone, clavicle and ankle in late childhood, compared with those born 2 years after the termination of vitamin D fortification, allowing for a washout period after termination. In addition, it was hypothesised that the vitamin D fortification during sundeprived months of gestation would be associated with the greatest risk reduction of offspring childhood fractures"	
	**Study design** *(RCT/ CCT/ cohort/ controlled before-after/ interrupted time series)*	prospective, birth cohort<br>observational study, exposure (natural experiment)	
	**Unit of allocation** *(by individuals, cluster/ groups or body parts)*	birth cohort<br>exposure (natural experiment)	
	**Number of study arms**	5: run-in, exposed, washout, non-exposed, late period	
	**Number of study centres**	not applicable	
	**Start date of study**	not applicable	
	**End date of study**	not applicable	
**Participants**	**Population description** *(from which study participants are drawn)*	"All individuals born alive in Denmark from 1 January 1983 to 31 December 1988 were included in the study."	
	**Country where trial was performed**	Denmark	
	**Location/Setting**	-	
	**Number of study centers**	not applicable	
	**Inclusion criteria**	All individuals born alive in Denmark from 1 January 1983 to 31 December 1988 were included in the study.	
	**Exclusion criteria**	born before and born after the exposure time	
	**Number of participants**	Number of contacted persons	not applicable
		Agreed to participate	not applicable
		Sample size *(started the study)*	327,254 (ages: 10-18)
		Lost to follow-up/withdrawals/drop outs	not applicable
		Number of participant analysed	not defined
		Sample size of total group	217 983,<br>exposed and non-exposed: 104 406+113 577
		Sample size of each groups	Run-in: 21432<br>Exposed: 104406<br>Washout: 68888<br>Non-exposed: 113577<br>Late: 18951
	**Sex** *(Male/Female; Boys/Girls)*	boys and girls	
	**Mean age (age range)**	10-18 years	
	**Race/Ethnicity**	not define	
	**Other relevant baseline data** *(e.g. BMI, health status, pregnant ect.)*	not define	
	**Subgroups measure**	not define	
	**Subgroups reported**	subgroups by sex	
**Interventions**	**Intervention(s)**	„The fact that until 1 June 1985 it was mandatory in Denmark to fortify all margarine with vitamin D. Margarine was fortified with 1·25 μg/100 g and approximately 13 % (3–29 %) of all dietary vitamin D is estimated to have come from the fortified margarine."	
	**Comparator(s)**	not exposed period	
	**Dosage** *(e.g recorded by FFQ)*	margarine fortification with 1.25 μg vitamin D/100 g margarine	
	**Fortified oil**	margarine	

	(e.g type, name ect.)	
	**Duration of intervention**	run-in (Jan 1983 – May 1983), exposed (June 1983 – May 1985), washout June 1985 – Aug 1986), non-exposed (Sep 1986 – Aug 1988), late period (Sep 1988 – Dec 1988)
	**Duration of follow-up**	Follow-up time for fractures for each participant started at age 10 years and ended at death, emigration, disappearance or age 18 years, whichever came first.
	**Run-in period**	5 mounths
	**Concomitant intervention**	"We did not identify other abrupt societal changes during 1983–1988 that potentially could influence our results, neither in relation to fortification practices in other food products for consumption, nor in relation to margarine intake in the Danish population or in relation to national recommendations for vitamin D supplementation to pregnant women or infants."<br>"prenatal exposure to extra vitamin D from fortification is assumed to be the only parameter that separates the individuals in the two exposure groups"<br><br>"During the study period margarine was also fortified with vitamin A, and the new evidence reveals that the vitamin A dose was increased by 25 % (from 20 to 25 IU/g) in 1962. There were also minor increases in 1971 from 25 to 26 IU/g and in 1985 from 26 to 28 IU/g." (Jensen, 2016)
	**Timing** *(e.g. frequency, duration of each episode)*	not applicable

		**Description**
**Outcomes**	**Primary outcomes defined by the authors**	Number of fracture events<br>fracture of the forearm, wrist, scaphoid, clavicle bone or ankle
	**Secondary outcomes defined by the authors**	Risk time (person-years)
	**Confounder** *(uncontrolled, but not adjusted; list of variables authors adjusted variables; Methods used to control to confounders)*	sunshine hours in Copenhagen

		**Description**		
**Outcome 1.**	**Outcome name**	number of fracture events		
	**Outcome definition** *(with diagnostic criteria if relevant)*	"The main study outcome was fracture of the forearm, wrist or scaphoid bone (International Classification of Diseases (ICD)-10: S52, S62·0); fracture of the clavicle (ICD-10: S42·0); and fracture of the ankle (ICD-10: S82·5, S82·6, S82·8). From 1994, ICD-10 diagnoses were classified according to the WHO International Classification of Diseases, and from 1 January 1995 the outpatient and emergency room contacts were mandatorily included in the registers"		
	**Time point** *(specify from start or end of intervention) for example 3,6,9,12*	not applicable		
	**Unit of analysis** *(individuals, cluster/ groups or body parts)*	individuals		
	**Post-intervention or change from baseline?**	not applicable		
	**Unit of measurement**	%		
	**Results**	**Intervention group (Exposed)**		
		**Number of participants**	**Number of fracture events**	**%**
		104406	12330	11.81
		**Comparison group (Non-exposed)**		
		**Number of participants**	**Number of fracture events**	**%**
		113577	16058	14.14
	**Boys Results**	**Intervention group (Exposed)**		
		**Number of participants**	**Number of fracture events**	**%**
		53555	7636	14.26
		**Comparison group (Non-exposed)**		
		**Number of participants**	**Number of fracture events**	**%**
		58542	9623	16.44
	**Girls Results**	**Intervention group (Exposed)**		
		**Number of participants**	**Number of fracture events**	**%**
		50851	4694	9.23
		**Comparison group (Non-exposed)**		
		**Number of participants**	**Number of fracture events**	**%**

		55035	6435	11.69
	**Forearm, wrist or scaphoid bone fracture**<br><br>**Results**	**Intervention group (Exposed)**		
		**Number of participants**	**Number of fracture events**	**%**
		104406	9315	8.92
		**Comparison group (Non-exposed)**		
		**Number of participants**	**Number of fracture events**	**%**
		113577	12469	10.98
	**Forearm, wrist or scaphoid bone fracture**<br><br>**Boys**<br>**Results**	**Intervention group (Exposed)**		
		**Number of participants**	**Number of fracture events**	**%**
		53555	5706	10.65
		**Comparison group (Non-exposed)**		
		**Number of participants**	**Number of fracture events**	**%**
		58542	7361	12.57
	**Forearm, wrist or scaphoid bone fracture**<br><br>**Girls**<br>**Results**	**Intervention group (Exposed)**		
		**Number of participants**	**Number of fracture events**	**%**
		50851	3609	7.1
		**Comparison group (Non-exposed)**		
		**Number of participants**	**Number of fracture events**	**%**
		55035	5108	9.28
	**Ankle fracture**<br><br>**Results**	**Intervention group (Exposed)**		
		**Number of participants**	**Number of fracture events**	**%**
		104406	1906	1.83
		**Comparison group (Non-exposed)**		
		**Number of participants**	**Number of fracture events**	**%**
		113577	2159	1.9
	**Ankle fracture**<br><br>**Boys**<br>**Results**	**Intervention group (Exposed)**		
		**Number of participants**	**Number of fracture events**	**%**
		53555	1101	2.06
		**Comparison group (Non-exposed)**		
		**Number of participants**	**Number of fracture events**	**%**
		58542	1241	2.12
	**Ankle fracture**<br><br>**Girls**<br>**Results**	**Intervention group (Exposed)**		
		**Number of participants**	**Number of fracture events**	**%**
		50851	805	1.58
		**Comparison group (Non-exposed)**		
		**Number of participants**	**Number of fracture events**	**%**
		55035	918	1.67
	**Clavicle fracture**<br><br>**Results**	**Intervention group (Exposed)**		
		**Number of participants**	**Number of fracture events**	**%**
		104406	1109	1.06
		**Comparison group (Non-exposed)**		
		**Number of participants**	**Number of fracture events**	**%**
		113577	1430	1.26
	**Clavicle fracture**<br><br>**Boys**<br>**Results**	**Intervention group (Exposed)**		
		**Number of participants**	**Number of fracture events**	**%**
		53555	829	1.55
		**Comparison group (Non-exposed)**		
		**Number of participants**	**Number of fracture events**	**%**
		58542	1021	1.74
	**Clavicle fracture**<br><br>**Girls**<br>**Results**	**Intervention group (Exposed)**		
		**Number of participants**	**Number of fracture events**	**%**
		50851	280	0.55
		**Comparison group (Non-exposed)**		
		**Number of participants**	**Number of fracture events**	**%**
		55035	409	0.74

	**Any other results reported** *(e.g. mean difference, CI, P value)*	"sustained a fracture with an overall fracture rate of 19·4 (95 % CI 19·1, 19·7) and 18·4 (95 % CI 18·1, 18·7) per 1000 person-years among the exposed and non-exposed individuals, respectively"<br><br>*<u>Fracture risk across age groups and seasonality in fracture occurrence</u>*:<br>• Boys: overall average fracture rate was 22·5/1000 person years, with a peak fracture rate of 35·1/1000 person-years between the ages of 13 and 14 years<br>• Girls: overall average fracture rate was 14·6/1000 person years, with a peak fracture rate of 27·9/1000 person-years between the ages of 11 and 12 years (<br>• The peak age of the ankle and clavicle fracture rates occurred later compared with the overall fracture rate, but was similar for both girls (range of 12–14 years) and boys (range of 15–16 years).<br>• Ankle: overall average fracture rate was 2·7/1000 person-years<br>• Clavicle: overall average fracture rate 1·7/1000 person-years<br>• Forearm, wrist or scaphoid bone fractures showed a similar pattern as the overall analysis, resulting from the high incidence rate of forearm, wrist or scaphoid bone fractures in children, which was the main contribution to the fracture outcome<br>• For both girls and boys: almost 2-fold increase in the fracture rate when comparing the months with the highest rates (April, May or August) and the month with the lowest rate (December)<br>*„The estimates from the age-period model revealed a seasonal fracture pattern with increased risk during spring and early fall and with a nadir during winter in the period from 1996 to 2007*<br><br><u>Fracture rates compared between individuals potentially exposed to vitamin D fortification and non-exposed individuals:</u><br>- Girls: exposed was 1·15 (95% CI 1·11, 1·20) compared with the non-exposed<br>- Boys: exposed compared with the non-exposed was 1·11 (95% CI 1·07, 1·14)<br>- Girls: RR 1·01 (95% CI 0·96, 1·05)<br>- Boys: RR 1·01 (95% CI 0·98, 1·04)<br>no interaction between season of birth and the exposure to vitamin D fortification in relation to overall fracture risk (girls: P 0·23; boys: P 0·44)<br><br>"In total, 12 330 exposed and 16 058 non-exposed individuals sustained a fracture with an overall fracture rate of 19·4 (95% CI 19·1, 19·7) and 18·4 (95% CI 18·1, 18·7) per 1000 person-years among the exposed and non-exposed individuals, respectively."<br>„The fracture type with the highest incidence was forearm, wrist or scaphoid bone, with 9315 events in the exposed group and 12 469 events in the non-exposed group."

	**Conclusion**
**Conclusion**	**Händel (2017):** „The study did not provide evidence that prenatal exposure to extra vitamin D from a mandatory fortification programme, adding1·25 µg vitamin D/100 g margarine, was sufficient to influence the risk of fractures in late childhood, regardless of season of birth. Replication studies are needed. There was a decreasing trend in fracture events occurring in the birth cohort of 1983–1988, which might be explained by secular trends of bicycle accidents in the period, rather than by differences in the birth cohorts."

		Description
**Study Characteristics**	**Study ID**	#1922
	**Author, date**	**Jensen, 2015**
	**Publication type** *(e.g. full report, abstract, letter)*	Full report
	**Language of publication**	English
	**Funding**	„funded by the Danish Agency for Science Technology and Innovation, the Ministry of Science, Innovation and Higher Education, under the instruments 'Strategic Research Projects' and by a research grant from the Danish PhD School of Molecular Metabolism funded by the Novo Nordisk Foundation."
	**Conflict** *(notable conflicts of interest of study authors)*	no conflict
**Methods**	**Aim of study**	„The aim of the present study was to elaborate on these results, and to investigate whether prenatal exposure to vitamin-D fortification of margarine and low-fat milk also was associated with body size at 7 years of age."
	**Study design** *(RCT/ CCT/ cohort/ controlled before-after/ interrupted time series)*	birth cohort<br>societal experiment<br><br>Study design has been described in detail previously. [10, 11]<br><br>*[10] Jacobsen R, Abrahamsen B, Bauerek M, Holst C, Jensen CB, Knop J et al. The influence of early exposure to vitamin D for development of diseases later in life. BMC Public Health 2013; 13: 515.*<br>*[11] Jensen CB, Berentzen TL, Gamborg M, Sørensen TIA, Heitmann BL. Does prenatal exposure to vitamin D-fortified margarine and milk alter birth weight? A societal experiment. Br J Nutr 2014; 112: 785–793*
	**Unit of allocation** *(by individuals, cluster/ groups or body parts)*	groups
	**Number of study arms**	2<br>exposed, non-exposed
	**Number of study centres**	–<br>national fortification program, selected from a register<br><br>„selected from the Copenhagen School Health Record Register (CSHRR)"
	**Start date of study**	–<br>Margarine fortification was initiated on 1 January 1961
	**End date of study**	–<br>Margarine fortification was terminated on 31 May 1985.
**Participants**	**Population description** *(from which study participants are drawn)*	Exposed and non-exposed population of boys and girls (schoolchildren) in the CSHRR, Copenhagen born during 1930–1989
	**Country where trial was performed**	Denmark
	**Location/Setting**	Copenhagen
	**Inclusion criteria**	„all children attending Copenhagen public and private schools were included."<br>"The CSHRR includes all schoolchildren in Copenhagen Municipality born in 1930–1989 and comprises of 372 636 records."
	**Exclusion criteria**	- children were born >2 years (plus the wash-out period) before or after a fortification event<br>- because of missing information on BMI Z-score.
	**Number of participants**	Number of contacted persons: no contact, selected from a register: 372 636<br>Agreed to participate: -<br>Sample size *(started the study)*: 61 914<br>Lost to follow-up/withdrawals/drop outs: 318 335 (310 691 + 7 644)<br>Number of participant analysed: 54 270<br>Sample size of total group: 30 004<br>Sample size of each groups: Initiation of margarine fortification: 18 357; Termination of milk fortification: 10 832
	**Sex** *(Male/Female; Boys/Girls)*	boys, girls
	**Mean age (age range)**	–<br>(7 years of age)
	**Race/Ethnicity**	–
	**Other relevant baseline data**	–

	(e.g. BMI, health status, pregnant ect.)	
	**Subgroups measure**	–
	**Subgroups reported**	–
**Interventions**	**Intervention(s)**	„Margarine was fortified with 1.25 µg vitamin-D per 100 g from 1st January 1961 to 1st June 1985, and it was allowed to fortify low-fat milk with 0.25–0.38 µg vitamin-D per 100 g from 1st January 1972 to 1st January 1976.”
	**Comparator(s)**	„compared the two groups of children from each fortification event”
	**Dosage** *(e.g recorded by FFQ)*	margarine was fortified with 1.25 µg vitamin-D per 100 g from 1st January 1961 to 1st June 1985<br><br>„*During the study period margarine was also fortified with vitamin A, and the new evidence reveals that the vitamin A dose was increased by 25% (from 20 to 25 IU/g) in 1962(3). There were also minor increases in 1971 from 25 to 26 IU/g(8) and in 1985 from 26 to 28 IU/g(4). (Jensen, 2016 Erratum)*”
	**Fortified oil** *(e.g type, name ect.)*	–
	**Duration of intervention**	01 June 1983 and 31 May 1985
	**Duration of follow-up**	–
	**Run-in period**	‘wash-out’ period of 6 months (margarine)
	**Concomitant intervention**	“In a nationwide dietary study from 1985 the vitamin-D fortification of margarine was estimated to have contributed to ~ 13% (3–29%) of the total vitamin-D intake in the Danish population”<br><br>“For margarine a ‘wash-out’ period of 6 months was applied after which it was assumed that all margarine produced before the fortification became mandatory had been replaced from stores and households with fortified margarine in 1961 and the opposite in 1985. Margarine had an average shelf life of about 4 months and an additional 2 months were added to account for the margarine that was used to prepare foods that could be stored beyond the shelf life of margarine”<br><br>“During the study period margarine was also fortified with vitamin A, and the new evidence reveals that the vitamin A dose was increased by 25 % (from 20 to 25 IU/g) in 1962. There were also minor increases in 1971 from 25 to 26 IU/g and in 1985 from 26 to 28 IU/g.”<br>*Therefore, we request the editor and readers to disregard the estimates based on analyses in 1961 and 1976, and to keep in mind that changes in vitamin A fortification also took place (Fig. 1). (Jensen 2016, Erratum)*
	**Timing** *(e.g. frequency, duration of each episode)*	four fortification events<br><br>/that is, initiation and termination of margarine and milk fortification we selected 2-year birth cohorts before and after/

		Description
**Outcomes**	**Primary outcomes defined by the authors**	Birth weight, BMI, BMI Z-score, prevalence of overweight and obesity (at 7 years of age)
	**Secondary outcomes defined by the authors**	–
	**Confounder** *(uncontrolled, but not adjusted; list of variables authors adjusted variables; Methods used to control to confounders)*	“Adjustment for birth weight in all analyses showed that birth weight was a statistically significant predictor of BMI Z-score, overweight and obesity (P-value o0.05), but associations between prenatal vitamin-D and body size at age 7 years were essentially similar before and after the inclusion of this variable.”

		Description			
**Outcome 1.**	**Outcome name**	Birth weight			
	**Outcome definition** *(with diagnostic criteria if relevant)*	–			
	**Time point** *(specify from start or end of intervention) for example 3,6,9,12*	Termination of margarine fortification			
	**Unit of analysis** *(individuals, cluster/ groups or body parts)*	exposed cohort vs. nonexposed cohort			
	**Post-intervention or change from baseline?**	Post intervention and mean differences			
	**Unit of measurement**	Mean values and standard deviations; number of subjects and percentages			
	**Results**	**Intervention group**			
		**Time point**	**Mean** *(if it Median than correct*	**SD** *(or other*	**No. participants**

			this part please to that)	*variance, specify)*	
		**Birth weight (g) Total**	3333	551	54 270
		**Margarin fortification, inititation, exposed**	3334	560	8717
		**Margarin fortification, termination, exposed**	3324	548	5029
		**Comparison group**			
		**Time point**	**Mean** *(if it Median than correct this part please to that)*	**SD** *(or other variance, specify)*	**No. participants**
		**Margarin fortification, inititation, non-exposed**	3307	567	9640
		**Margarin fortification, termination, non-exposed**	3365	549	5803
	**Any other results reported** *(e.g. mean difference, CI, P value)*	–			

**Outcome 1**.		**Description**			
	**Outcome name**	BMI, BMI Z-score			
	**Outcome definition** *(with diagnostic criteria if relevant)*	"Body mass index (BMI) was calculated for each child as weight (kg)/height2 (m) and transformed to a Z-score based on an internal age and sex-specific reference that was computed using the LMS method"			
	**Time point** *(specify from start or end of intervention) for example 3,6,9,12*	Not applicable			
	**Unit of analysis** *(individuals, cluster/ groups or body parts)*	groups			
	**Post-intervention or change from baseline?**	Post intervention			
	**Unit of measurement**	mean ± SD; kg/m2			
	**Results**	**Intervention group**			
		**Time point**	**Mean** *(if it Median than correct this part please to that)*	**SD** *(or other variance, specify)*	**No. participants**
		**BMI** initiation	15.4	1.4	8717
		**BMI** termination	15.9	1.7	5029
		**BMI Z-score** (mean ± SD) termination	0.3	1.1	5029
		Number of 7-year olds with birth weight (%) initiation, exposed	8010	92	8717
		Number of 7-year olds with birth weight (%) termination, exposed	4432	88	5029
		**Comparison group**			
		**Time point**	**Mean** *(if it Median than correct this part please to that)*	**SD** *(or other variance, specify)*	**No. participants**
		**BMI** initiation	15.4	1.4	9640
		**BMI** termination,	16.0	1.8	5803
		**BMI Z-score** (mean ± SD) termination	0.3	1.2	5803

		Number of 7-year olds with birth weight (%) initiation, non-exposed	8733	91	9640
		Number of 7-year olds with birth weight (%) termination, non-exposed	5190	89	5803
	**Any other results reported** *(e.g. mean difference, CI, P value)*	"Mean BMI Z-score at age 7 years (mean = 0 in 1955–1960) increased across the period from − 0.1 in 1946 to 0.4 in 1989 (202 075 observations)"<br>"by conventional standards almost all children at 7 years of age were within the normal weight range, limiting the potential for vitamin-D fortification to have an impact."			

**Outcome 1.**		**Description**			
	**Outcome name**	Prevalence of overweight and obesity (at 7 years of age)			
	**Outcome definition** *(with diagnostic criteria if relevant)*	"Children were classified as overweight and obese if their BMI Z-score exceeded the Z-scores equivalent to the 85th percentile (1.0364) and the 95th percentile (1.6449) in the reference population, respectively."			
	**Time point** *(specify from start or end of intervention) for example 3,6,9,12*	initiation vs. termination			
	**Unit of analysis** *(individuals, cluster/ groups or body parts)*	individuals			
	**Post-intervention or change from baseline?**	post intervention			
	**Unit of measurement**	%, N, n			
	**Results**	**Intervention group**			
		**Time point**	**Mean** *(if it Median than correct this part please to that)*	**SD** *(or other variance, specify)*	**No. participants**
		Overweight Total			N=54 270<br>n=9606<br>17.7 %
		Obesity<br>Total			N=54 270<br>n=3962<br>7.3 %
		termination<br>Overweight			N=5029<br>n=1172<br>23.3 %
		termination<br>Obesity			N=5029<br>n=523<br>10.4%
		**Comparison group**			
		**Time point**	**Mean** *(if it Median than correct this part please to that)*	**SD** *(or other variance, specify)*	**No. participants**
		termination<br>Overweight			N=5803<br>n=1468<br>25.3%
		termination<br>Obesity			N=5803<br>n=708<br>12.2%
	**Any other results reported** *(e.g. mean difference, CI, P value)*	"Mean BMI Z-score at age 7 years (mean = 0 in 1955–1960) increased across the period from − 0.1 in 1946 to 0.4 in 1989 (202 075 observations). Odds of overweight and obesity also increased across the period."<br>"compared BMI z-scores and odds ratio of overweight and obesity at 7 years of age among children born around each fortification event: none of these measures showed any association with exposure to vitamin-D-fortified foods during pregnancy."<br><br>"Odds ratio of overweight from the meta-analyses of the four fortification events was 0.97 (95% CI: 0.89–1.06) in the crude analyses and 0.97 (95% CI: 0.93–1.02) in the analyses adjusted for secular trend in overweight. Odds ratio of obesity was 0.96 (95% CI: 0.82–1.13) in the meta-analyses of the crude estimates and 1.00 (95% CI: 0.91–1.09) in the analyses adjusted for secular trend in			

		obesity"<br>"pooled estimate of mean difference in BMI Z-scores between exposed and non-exposed children of − 0.02 (95% CI: − 0.07 to 0.03) in the crude analyses and of − 0.01 (95% CI: − 0.03 to 0.01) in the analyses adjusted for secular trend in mean BMI Z-score"<br><br>Meta-analyses of the four fortification events showed a pooled estimate of mean difference in BMI Z-scores between exposed and non-exposed children of − 0.02 (95% CI: − 0.07 to 0.03) in the crude analyses and of − 0.01 (95% CI: − 0.03 to 0.01) in the analyses adjusted for secular trend in mean BMI Z-score.

**Conclusion**	**Conclusion**
	We could not demonstrate that vitamin-D during prenatal life, from the national vitamin-D fortification programs of margarine and low-fat milk, influenced development of overweight and obesity at 7 years of age.

		Description	
**Study Characteristics**	**Study ID**	#1921	
	**Author, date**	***Jensen 2014***, Jensen 2016	
	**Publication type** *(e.g. full report, abstract, letter)*	Full report, erratum (Jensen 2016)	
	**Language of publication**	English	
	**Funding**	„funded by the Danish Agency for Science Technology and Innovation, the Ministry of Science, Innovation and Higher Education, under the instruments ‘Strategic Research Projects’ and by a research grant from the Danish PhD School of Molecular Metabolism funded by the Novo Nordisk Foundation.”	
	**Conflict** *(notable conflicts of interest of study authors)*	no conflict	
**Methods**	**Aim of study**	“In the present study, we aimed to study the impact of the Danish vitamin D fortification programmes on mean birth weight, as well as on the risk of high or low birth weight."	
	**Study design** *(RCT/ CCT/ cohort/ controlled before-after/ interrupted time series)*	birth cohort<br><br>Study design has been described in detail previously. [10, 11]<br><br>*[10] Jacobsen R, Abrahamsen B, Bauerek M, Holst C, Jensen CB, Knop J et al. The influence of early exposure to vitamin D for development of diseases later in life. BMC Public Health 2013; 13: 515.*<br>*[11] Jensen CB, Berentzen TL, Gamborg M, Sørensen TIA, Heitmann BL. Does prenatal exposure to vitamin D-fortified margarine and milk alter birth weight? A societal experiment. Br J Nutr 2014; 112: 785–793*<br><br>Study protocol:<br>*[44]Jacobsen R, Abrahamsen B, Bauerek M, et al. (2013) The influence of early exposure to vitamin D for development of diseases later in life. BMC Public Health 13, 515.*	
	**Unit of allocation** *(by individuals, cluster/ groups or body parts)*	groups	
	**Number of study arms**	2<br>exposed, non-exposed	
	**Number of study centres**	–<br>national fortification program, selected from a register<br><br>„selected from the Copenhagen School Health Record Register (CSHRR)”, „The CSHRR includes all schoolchildren in Copenhagen Municipality born in 1930–1989 and comprises of 372 636 records.”	
	**Start date of study**	–<br>Margarine fortification was initiated on 1 January 1961	
	**End date of study**	–<br>Margarine fortification was terminated on 31 May 1985.	
**Participants**	**Population description** *(from which study participants are drawn)*	Exposed and non-exposed population of boys and girls (schoolchildren) in the CSHRR, Copenhagen born during 1930–1989	
	**Country where trial was performed**	Denmark	
	**Location/Setting**	Copenhagen	
	**Inclusion criteria**	“The study population was selected from the CSHRR, which includes virtually every school child in Copenhagen born from 1930 to 1989 and comprises in total 372 636 records.”<br>“included all children selected from the CSHRR who were born during a 2-year period before or subsequent to the initiation and termination of the fortification programmes.”	
	**Exclusion criteria**	- “Children for whom information on birth weight was not available and children born outside the period relevant for studying the effects of the vitamin D fortification programmes (1959–1988)”<br>- “Extreme values of birth weight (<1.5 and>5.5 kg) were excluded as part of the data cleaning of the register.”<br>- “Children who did not experience an entire trimester during winter were excluded from the secondary analyses, as were children who experienced more than one entire trimester during winter.”	
	**Number of participants**	Number of contacted persons	no contact, selected from a register:<br>372 636
		Agreed to participate	–
		Sample size *(started the study)*	61 914
		Lost to follow-up/withdrawals/drop outs	320 753 (310 722 + 10 031)
		Number of participant analysed	51 883
		Sample size of total group	28 198

		Sample size of each groups	Initiation of margarine fortification:<br>17 646<br>Termination of milk fortification:<br>10 552
	**Sex**<br>*(Male/Female; Boys/Girls)*	boys, girls	
	**Mean age**<br>**(age range)**	–	
	**Race/Ethnicity**	–	
	**Other relevant baseline data**<br>*(e.g. BMI, health status, pregnant ect.)*	"by conventional standards almost all children at 7 years of age were within the normal weight range, limiting the potential for vitamin-D fortification to have an impact." (*Jensen, 2016 Erratum*)	
	**Subgroups measure**	–	
	**Subgroups reported**	–	
**Interventions**	**Intervention(s)**	„Margarine was fortified with 1.25 μg vitamin-D per 100 g from 1st January 1961 to 1st June 1985, and it was allowed to fortify low-fat milk with 0.25–0.38 μg vitamin-D per 100 g from 1st January 1972 to 1st January 1976."<br><br>*Therefore, we request the editor and readers to disregard the estimates based on analyses in 1961 and 1976, and to keep in mind that changes in vitamin A fortification also took place (Fig. 1). (Jensen 2016, Erratum)*	
	**Comparator(s)**	„compared the two groups of children from each fortification event"	
	**Dosage**<br>*(e.g recorded by FFQ)*	Margarine was fortified with 1.25 μg vitamin-D per 100 g from 1st January 1961 to 1st June 1985<br><br>„*During the study period margarine was also fortified with vitamin A, and the new evidence reveals that the vitamin A dose was increased by 25% (from 20 to 25 IU/g) in 1962(3). There were also minor increases in 1971 from 25 to 26 IU/g(8) and in 1985 from 26 to 28 IU/g(4). (Jensen, 2016 Erratum)*"	
	**Fortified oil**<br>*(e.g type, name ect.)*	–	
	**Duration**<br>**of intervention**	–	
	**Duration of**<br>**follow-up**	–	
	**Run-in period**	'wash-out' period of 6 months (margarine)	
	**Concomitant intervention**	"In a nationwide dietary study from 1985 the vitamin-D fortification of margarine was estimated to have contributed to ~ 13% (3–29%) of the total vitamin-D intake in the Danish population"<br><br>"For margarine a 'wash-out' period of 6 months was applied after which it was assumed that all margarine produced before the fortification became mandatory had been replaced from stores and households with fortified margarine in 1961 and the opposite in 1985. Margarine had an average shelf life of about 4 months and an additional 2 months were added to account for the margarine that was used to prepare foods that could be stored beyond the shelf life of margarine"<br><br>"During the study period margarine was also fortified with vitamin A, and the new evidence reveals that the vitamin A dose was increased by 25 % (from 20 to 25 IU/g) in 1962. There were also minor increases in 1971 from 25 to 26 IU/g and in 1985 from 26 to 28 IU/g." (*Jensen, 2016 Erratum)*	
	**Timing**<br>*(e.g. frequency, duration of each episode)*	four fortification events<br><br>/that is, initiation and termination of margarine and milk fortification we selected 2-year birth cohorts before and after/	

		**Description**
**Outcomes**	**Primary outcomes defined by the authors**	Birth weight, prevalence of low and high birth weight
	**Secondary outcomes defined by the authors**	–
	**Confounder** *(uncontrolled, but not adjusted; list of variables authors adjusted variables; Methods used to control to confounders)*	"Adjustment for birth weight in all analyses showed that birth weight was a statistically significant predictor of BMI Z-score, overweight and obesity (P-value o0.05), but associations between prenatal vitamin-D and body size at age 7 years were essentially similar before and after the inclusion of this variable."

		**Description**
**Outcome 1.**	**Outcome name**	Birth weight, prevalence of low and high birth weight
	**Outcome definition** *(with diagnostic criteria if relevant)*	Low birth weight (<2500 g), High birth weight (>4000 g)

	**Time point** *(specify from start or end of intervention) for example 3,6,9,12*	Termination of margarine fortification				
	**Unit of analysis** *(individuals, cluster/ groups or body parts)*	exposed cohort vs. nonexposed cohort				
	**Post-intervention or change from baseline?**	Post intervention and mean differences				
	**Unit of measurement**	Mean values and standard deviations; number of subjects and percentages				
	**Results** *Jensen, 2014* *See in Text in Table1*	**Intervention group**				
		**Time point**	**Mean** *(if it Median than correct this part please to that)*	**SD** *(or other variance, specify)*	**No. participants**	
		Birth weight (g)	3324	549	4836	
		Low birth weight (<2500 g)			301	6.2%
		High birth weight (>4000 g)			407	8.4%
		**Comparison group**				
		**Time point**	**Mean** *(if it Median than correct this part please to that)*	**SD** *(or other variance, specify)*	**No. participants**	
		Birth weight (g)	3367	549	5716	
		Low birth weight (<2500 g)			308<br>5.4%	
		High birth weight (>4000 g)			583<br>10.2%	
	**Any other results reported** *(e.g. mean difference, CI, P value)*	We found no difference in risk of low and high birth weight between exposed and non-exposed children. Contrary to our expectations, we found inconsistent effects of the vitamin D fortification on mean birth weight; the mean birth weight was lower among exposed than non-exposed in all analyses except in the analyses based on margarine fortification in 1961 (Fig. 2). However, the mean difference in birth weight did not exceed 60 grams, and we questioned the clinical relevance." *(Jensen 2016, Erratum)*				

**Conclusion**	**Conclusion**
	"In summary, prenatal exposure to vitamin D from fortified margarine and milk altered birth weight, but the effect on birth weight was small and inconsistent."

		Description	
Study Characteristics	**Study ID**	#3986	
	**Author, date**	**Stougaard, 2017**	
	**Publication type** *(e.g. full report, abstract, letter)*	Full report	
	**Language of publication**	English	
	**Funding**	"Funded by the Danish Agency for Science, Technology and Innovation, the Ministry of Science, Higher Education, under the instrument "Strategic Research Projects" (grant previous 11-116213 now 0603-00453B) and by the PhD School of Faculty of Health Sciences the University of Southern Denmark."	
	**Conflict** *(notable conflicts of interest of study authors)*	None	
Methods	**Aim of study**	The purpose of the present study was to examine if fetal exposure to extra vitamin D from fortified margarine lowered the risk of pre-eclampsia later in life.	
	**Study design** *(RCT/ CCT/ cohort/ controlled before-after/ interrupted time series)*	Birth cohort study	
	**Unit of allocation** *(by individuals, cluster/ groups or body parts)*	group	
	**Number of study arms**	2<br>Exposed group - unexposed group	
	**Number of study centres**	National data sources<br>The Danish Civil Registration System (CSR), Denmark with a 10-digit civil person register (CPR), The Danish Medical Birth Registry (MBR), The Danish National Patient Registry (DNPR)	
	**Start date of study**	1st of June 1983	
	**End date of study**	not applicable	
Participants	**Population description** *(from which study participants are drawn)*	Women born from June 1983 to May 1985 and giving birth at age 14·5 to 27·5 years and delivering their offspring after gestational week 22, was defined as exposed to extra vitamin D during fetal development born from September 1986 to August 1988 and giving birth at age 14·5 to 27·5 years and delivering their offspring after gestational week 22, was defined as unexposed.	
	**Country where trial was performed**	Denmark	
	**Location/Setting**	national data, Denmark	
	**Inclusion criteria**	"the cohort of women born in the two years immediately before June 1985, born from June 1983 to May 1985, was defined as exposed to extra vitamin D during fetal development and the cohort of women born after the washout period, born from September 1986 to August 1988, was defined as unexposed."<br>„women giving birth at age 14·5 to 27·5 years and delivering their offspring after gestational week 22 to ensure similar age in both cohorts."	
	**Exclusion criteria**	"Newborns with birth weight of 0 g and very young women, as well as very young women with pre-pregnancy BMI below the established cut-offs for this age group"<br>"missing information on singleton and multiple births, offspring birth weight or gender, smoking status or gestational age were also excluded"	
	**Number of participants**	Number of contacted persons	no contacts, national data<br>Stougaard B:<br>*n* 162 397
		Agreed to participate	not applicable
		Sample size *(started the study)*	Total: 85 308<br>Exposed: 52 873<br>("the number of births by women who, due to the mandatory margarine fortification, were exposed to additional vitamin D during fetal life was")<br>Non-exposed: 32 435<br>(The number of births by women unexposed to the extra vitamin D during fetal development was)
		Lost to follow-up/withdrawals/drop outs	Excluded births n=27,003 (Women excluded because they gave birth during the 15-month wash-out period from June 1985 to September 1986)<br>n=36,792 (Births where the woman was either below 14·5 or above 27·5 years of age, the offspring's

			birth weight was misclassified, gestational weeks was <22 weeks or pre-pregnancy BMI was <15·46 kg/m2)<br>n=348 (Excluded due to missing information on age at delivery, smoking habits,<br>singleton and multiple births, gestational age at delivery or offspring gender.)<br>n=15,267 (Births where the woman was either below 14·5 or above 27·5 years of age, the offspring's<br>birth weight was misclassified, gestational weeks was <22 weeks or pre-pregnancy BMI was <15·46 kg/m2.)<br>n=280 (Excluded due to missing information on age at delivery, smoking habits,<br>singleton and multiple births, gestational age at delivery or offspring gender.)<br>Total: 116.482
		Number of participant analysed	32 621<br>(15 733 exposed nulliparous women and<br>16 888 unexposed nulliparous women)
		Sample size of total group	32621
		Sample size of each groups	Stougaard B:<br>Exposed: 15733<br>Unexposed: 16888
	**Sex**<br>*(Male/Female; Boys/Girls)*	Female	
	**Mean age**<br>**(age range)**	„Despite the age restrictions, the age at delivery was slightly higher for the exposed women compared with unexposed; the median (5th–95th percentile) age being 24.9 (19.5–27.3) and 24.7 (19.4–27.3) years, respectively (P<0·0001)."<br>"the study population was restricted to women giving birth at age 14.5 to 27.5 years and delivering their offspring after gestational week 22 to ensure similar age in both cohorts."	
	**Race/Ethnicity**	not mentioned	
	**Other relevant baseline data**<br>*(e.g. BMI, health status, pregnant ect.)*	"Furthermore, exposed compared with unexposed women were more often current smokers (18·9 v. 15·8 %, respectively) and more often gave birth at late gestational age (6·1 v. 3·6 %, respectively). Moreover, exposed compared with unexposed women more often had a BMI<18·5 kg/m2 (4·4 v. 5·2 %, respectively) and less often had a BMI≥30·0 kg/m2 (13·6 v. 13·9 %, respectively). Other characteristics were not different between exposed and unexposed women."	
	**Subgroups measure**	smoking and non-smoking women ; by type of pre-eclampsia	
	**Subgroups reported**	smoking and non-smoking women ; by type of pre-eclampsia	
	**Intervention(s)**	Mandatory vitamin D fortification of margarine in Denmark until June 1985	
	**Comparator(s)**	No mandatory fortification of margarine in Denmark	
	**Dosage**<br>*(e.g recorded by FFQ)*	1.25µg of vitamin D per 100g margarine	
	**Fortified oil**<br>*(e.g type, name ect.)*	Margarine<br><br>„A Danish fortification policy that required adding of 1.25µg of vitamin D per 100g margarine, corresponding to approximately 13% (3–29%) of the daily vitamin D intake, was terminated June 1st 1985"	
	**Duration of intervention**	-<br>(from June 1983 to August 1988)	
**Interventions**	**Duration of follow-up**	-	
	**Run-in period**	„15 months' 'wash-out period' (consisting of a full 9 months of pregnancy and additional 6 months, securing that the fortified margarine was no longer available at home or in stores) was introduced and lasted from the 1st of June 1985 to the 31st of August 1986"	
	**Concomitant intervention**	During the study period margarine was also fortified with vitamin A, and the new evidence reveals that the vitamin A dose was increased by 25 % (from 20 to 25 IU/g) in 1962. There were also minor increases in 1971 from 25 to 26 IU/g and in 1985 from 26 to 28 IU/g. (Jensen, 2016)	
	**Timing**<br>*(e.g. frequency, duration of each episode)*	-	

		**Description**
**Outcomes**	**Primary outcomes defined by the authors**	„Definition of gestational hypertension, preeclampsia and eclampsia by ICD8 codes"<br><br>Group 1 Gestational hypertension (code 63700)<br>Group 2 Mild preeclampsia (code 63703)<br>Preeclampsia, unspecified (code 63709)<br>Toxemia (63799)

		Group 3 Severe preeclampsia (code 63704)<br>Eclampsia (code 63719)<br><br>"A woman could only be assigned one diagnosis; if a woman had more than one of the defined diagnoses, she was grouped according to the diagnosis code indicating the most severe diagnosis of the included outcomes."
	**Secondary outcomes defined by the authors**	-<br>"
	**Confounder** *(uncontrolled, but not adjusted; list of variables authors adjusted variables; Methods used to control to confounders)*	Months of delivery were categorized into 4 seasons: November to January (Winter); February to April (Spring); May to July (Summer) and August to October (Fall)"

**Outcome 1.**		**Description**			
	**Outcome name**	Gestational hypertension			
	**Outcome definition (with diagnostic criteria if relevant)**	Gestational hypertension (code 63700)			
	**Time point (specify from start or end of intervention) for example 3,6,9,12**	-			
	**Unit of analysis (individuals, cluster/ groups or body parts)**	group<br>Exposed group - unexposed group			
	**Post-intervention or change from baseline?**				
	**Unit of measurement**	n, %			
	**Results**	**Intervention group** Exposed			
		**Time point**	**N**	**n**	**%**
			15 733	235	1.5 %
		**Comparison group** Unexposed			
		**Time point**	**N**	**n**	**%**
			16 888	260	1.5 %
	**Any other results reported** *(e.g. mean difference, CI, P value)*	"lower risk of pregnancy-related hypertensive complications among women who were exposed to extra vitamin D in fetal development compared with the unexposed women."<br>„No significant associations were found for the diagnoses of gestational hypertension, severe pre-eclampsia, HELLP syndrome and eclampsia."			

**Outcome 1.**		**Description**			
	**Outcome name**	"risk of preeclampsia"			
	**Outcome definition** *(with diagnostic criteria if relevant)*	Group 2: Mild preeclampsia (code 63703)<br>Preeclampsia, unspecified (code 63709)<br>Toxemia (63799)<br><br>Group 3:<br>Severe preeclampsia (code 63704)<br>Eclampsia (code 63719)			
	**Time point** *(specify from start or end of intervention) for example 3,6,9,12*	-			
	**Unit of analysis** *(individuals, cluster/ groups or body parts)*	group<br>Exposed group - unexposed group			
	**Post-intervention or change from baseline?**	-			
	**Unit of measurement**	n,%			
	**Results**	**Intervention group**			
			**N**	**n**	**%**
		Group 2<br>Mild to moderate and unspecified pre-eclampsia	15,733	475	3.0 %
		Group 3<br>eclampsia, HELLP syndrome and eclampsia¶	15,733	185	1.2 %
		Stougaard B:<br>All Cases	15 733	895	5.7 %

	**Comparison group**			
		**N**	**n**	**%**
	Group 2<br>Mild to moderate and unspecified pre-eclampsia	16888	595	3.5 %
	Group 3<br>Severe pre-eclampsia, HELLP syndrome and eclampsia¶	16888	212	1.3 %
	All Cases	16888	1067	6.3 %
**Unit of measurement**	OR (95%CI)			
Mild to moderate and unspecified pre-eclampsia‡	Crude and adjusted odds for pre-eclampsia among women exposed to extra vitamin D in fetal life; women born between June 1983 and August 1988, who gave birth to their first child at age 14·5 to 27·5 years	Crude model<br>OR (95%CI)		
		0.85 (0.75, 0.96)		n= 1,070
		Adjusted model<br>OR (95%CI)		
		0.86 (0.76, 0.97)		n= 1,070
Severe pre-eclampsia, HELLP syndrome and eclampsia	Crude and adjusted odds for pre-eclampsia among women exposed to extra vitamin D in fetal life; women born between June 1983 and August 1988, who gave birth to their first child at age 14·5 to 27·5 years	Crude model<br>OR (95%CI)		
		0.94 (0.77, 1.14)		n= 397
		Adjusted model<br>OR (95%CI)		
		0.95 (0.78, 1.16)		n= 397
All cases	Crude and adjusted odds for pre-eclampsia among women exposed to extra vitamin D in fetal life; women born between June 1983 and August 1988, who gave birth to their first child at age 14·5 to 27·5 years	Crude model<br>OR (95%CI)		
		0·89 (0·82, 0·98)		n= 1962
		Adjusted model<br>OR (95%CI)		
		0·90 (0·82, 0·99)		n= 1962
**Any other results reported**<br>*(e.g. mean difference, CI, P value)*	- The interaction between exposure to vitamin D and smoking status was significant (χ2=11·82; df =2; P=0·003) and stratifying analyses on smoking status revealed that the strongest association between in utero vitamin D exposure status and pre-eclampsia risk was seen among current smokers, where the risk of all types of pre-eclampsia was halved or more for the exposed women compared with unexposed ones<br>- Sensitivity analyses in these sub-populations were restricted to women of similar age (age 20–27 years) and were adjusted for pre-pregnancy BMI; the results of these analyses were similar to the main findings.<br>- Interactions between exposure status and women's age at delivery (χ2=3·58; df=4; P =0·47), as well as between exposure status and pre-pregnancy BMI, were not significant (χ2 =0·77; df=3; P=0·86).			

	**Conclusion**
**Conclusion**	"The results of our study suggest that small extra doses of vitamin D from food fortification during fetal development may decrease the risk of developing pre-eclampsia during first pregnancy in adulthood. The beneficial effect of the extra vitamin D during fetal development appears to be particularly protective against pre-eclampsia for women who smoke during pregnancy."
