# Supplementary material for: Benefits and Harms of Edible Vegetable Oils and Fats Fortified with Vitamins A and D as a Public Health Intervention in the General Population: A Systematic Review of Interventions
Source: Nutrients. 2023 Dec 18;15(24):5135. doi: 10.3390/nu15245135 (PMC10745565; doi:10.3390/nu15245135)

Supplementary file S4: Risk of bias assessment

Figure 1. Risk of bias graph: review authors' judgements about each risk of bias item presented as percentages across all included randomised trials.

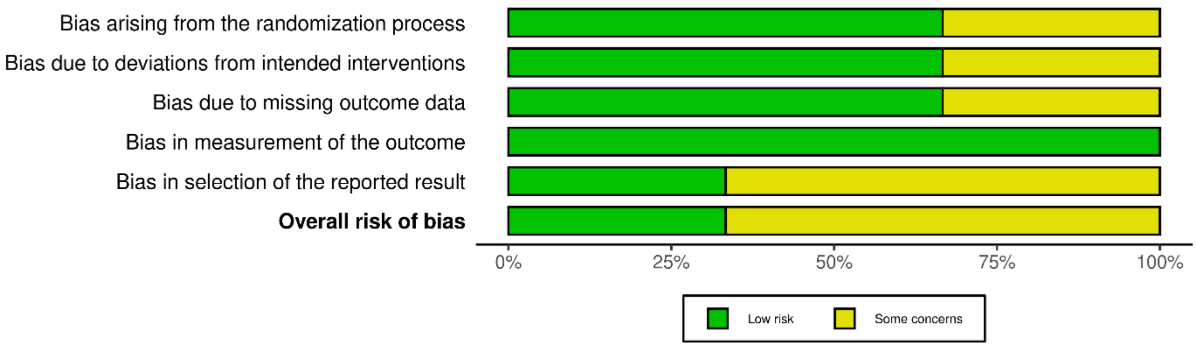

Figure 2. Risk of bias summary: review authors' judgements about each risk of bias item for each included randomised trial

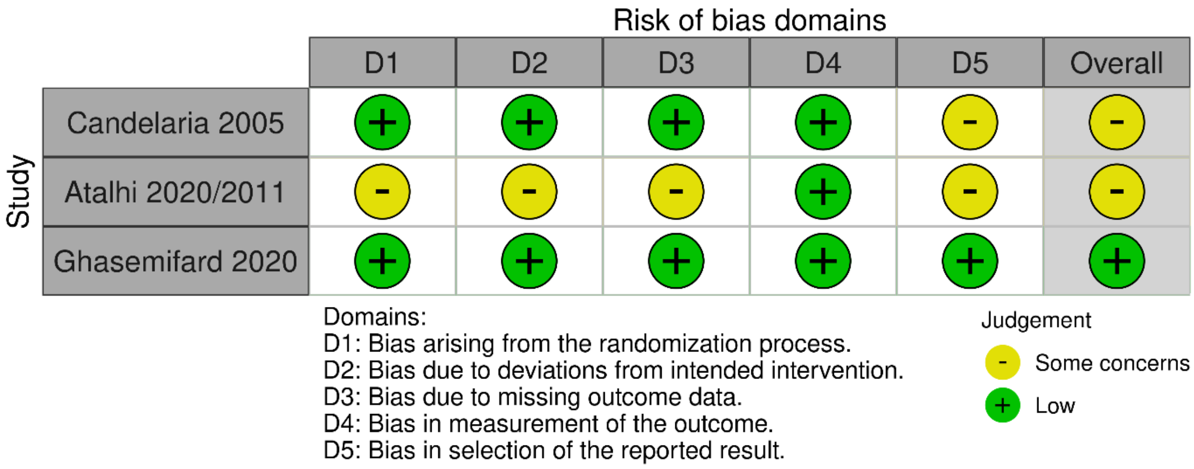

Figure 3. Risk of bias graph: review authors' judgements about each risk of bias item presented as percentages across all included non-randomised trials.

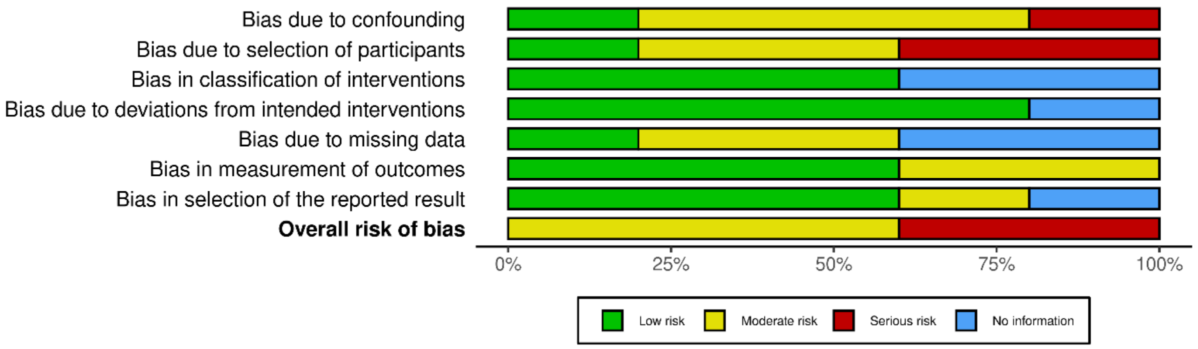

Figure 4. Risk of bias summary: review authors' judgements about each risk of bias item for each included non-randomised trial

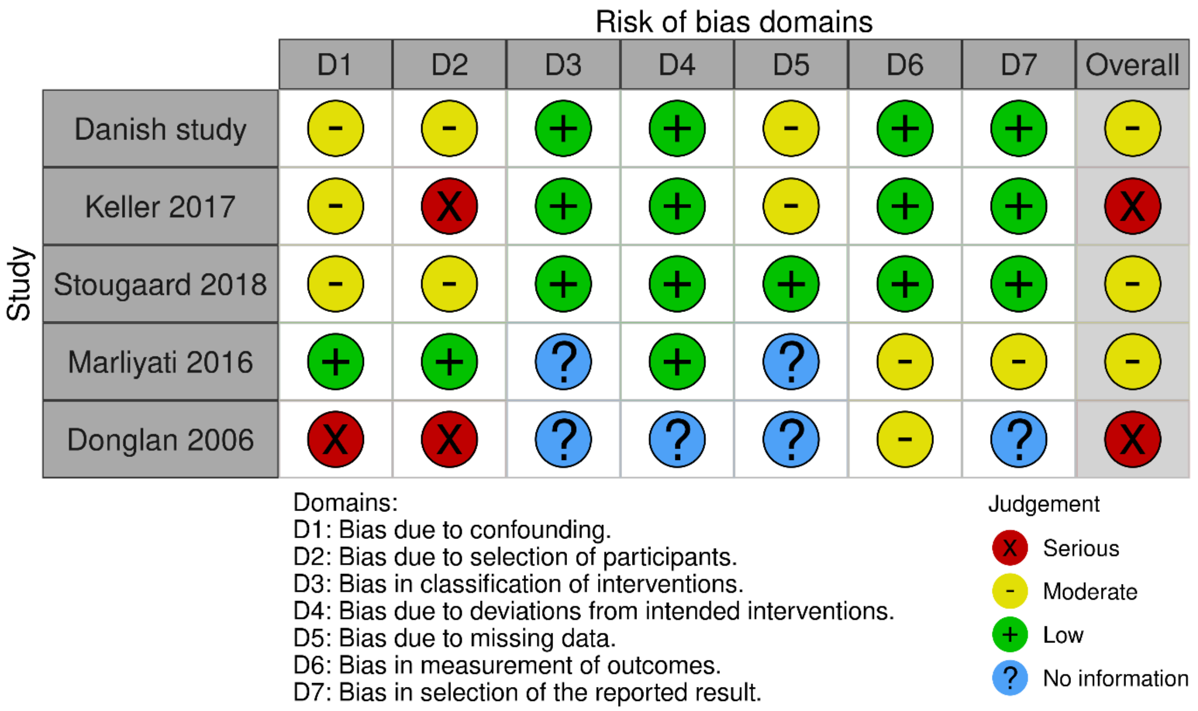

Supplement: Supplementary file 1 [file nutrients-15-05135-s001.zip › Supplementary File S4_RoB.pdf]
