# Supplementary material for: Benefits and Harms of Edible Vegetable Oils and Fats Fortified with Vitamins A and D as a Public Health Intervention in the General Population: A Systematic Review of Interventions
Source: Nutrients. 2023 Dec 18;15(24):5135. doi: 10.3390/nu15245135 (PMC10745565; doi:10.3390/nu15245135)

Supplementary file S5: Effect of Vitamin A fortified versus non-fortified oils and fats

Analysis 1.1 Vitamin A fortified versus non-fortified oils and fats. Outcome: Serum retinol (µmol/L)

	Vitamin A fortified			Non-fortified				Mean Difference
Study or Subgroup	Mean	SD	Total	Mean	SD	Total	Weight	IV, Random, 95% CI
**1.1.1 RCTs**								
Atalhi 2020	3.53	0.57	39	2.78	0.6	62	48.9%	0.75 [0.52, 0.98]
Candelaria 2005	1.32	0.25	268	1.36	0.29	145	51.1%	-0.04 [-0.10, 0.02]
**Subtotal (95% CI)**			**307**			**207**	**100.0%**	**0.35 [-0.43, 1.12]**
Heterogeneity: Tau² = 0.30; Chi² = 41.74, df = 1 (P < 0.00001); I² = 98%								
Test for overall effect: Z = 0.88 (P = 0.38)								
**1.1.2 CCTs**								
Donglan 2006	1.49	0.44	87	0.93	0.29	87	49.7%	0.56 [0.45, 0.67]
Marliyati 2016	0.56	0.11	15	0.5	0.12	16	50.3%	0.06 [-0.02, 0.14]
**Subtotal (95% CI)**			**102**			**103**	**100.0%**	**0.31 [-0.18, 0.80]**
Heterogeneity: Tau² = 0.12; Chi² = 51.03, df = 1 (P < 0.00001); I² = 98%								
Test for overall effect: Z = 1.23 (P = 0.22)								

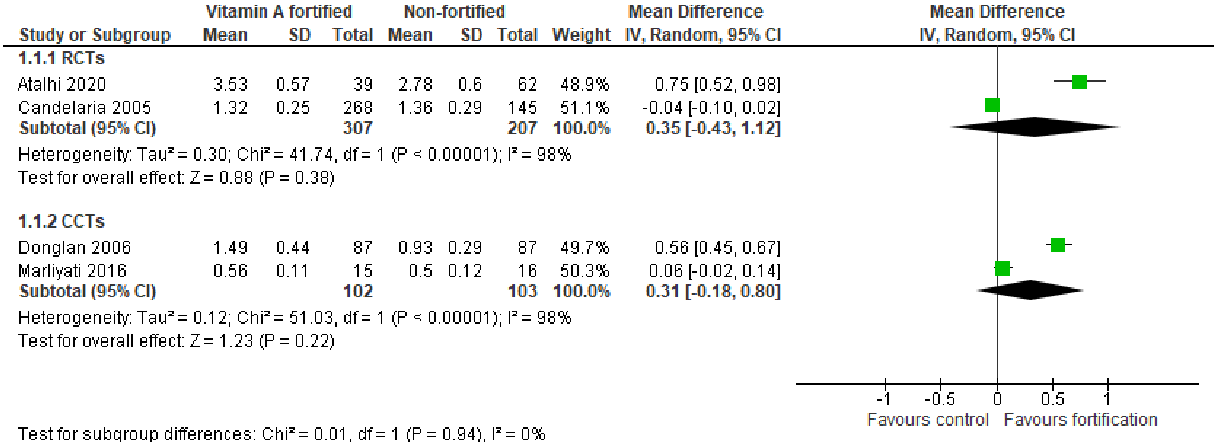

Test for subgroup differences: Chi² = 0.01, df = 1 (P = 0.94), I² = 0%

Analysis 1.2. Vitamin A fortified versus non-fortified oils and fats. Outcome: Serum retinol by age and physiological condition of the population

	Vitamin A fortified			Non-fortified				Mean Difference
Study or Subgroup	Mean	SD	Total	Mean	SD	Total	Weight	IV, Random, 95% CI
**1.2.2 School aged children (5-12 years)**								
Donglan 2006	1.49	0.44	87	0.93	0.29	87		Not estimable
Marliyati 2016	0.56	0.11	15	0.5	0.12	16		Not estimable
**Subtotal (95% CI)**			**0**			**0**		**Not estimable**
Heterogeneity: Not applicable								
Test for overall effect: Not applicable								
**1.2.3 Mixed preschool and school aged children**								
Candelaria 2005	1.32	0.25	268	1.36	0.29	145	100.0%	-0.04 [-0.10, 0.02]
**Subtotal (95% CI)**			**268**			**145**	**100.0%**	**-0.04 [-0.10, 0.02]**
Heterogeneity: Not applicable								
Test for overall effect: Z = 1.40 (P = 0.16)								
**1.2.4 Non-pregnant women of reproductive age (15-49 years)**								
Atalhi 2020	3.53	0.57	39	2.78	0.6	62	100.0%	0.75 [0.52, 0.98]
**Subtotal (95% CI)**			**39**			**62**	**100.0%**	**0.75 [0.52, 0.98]**
Heterogeneity: Not applicable								
Test for overall effect: Z = 6.31 (P < 0.00001)								

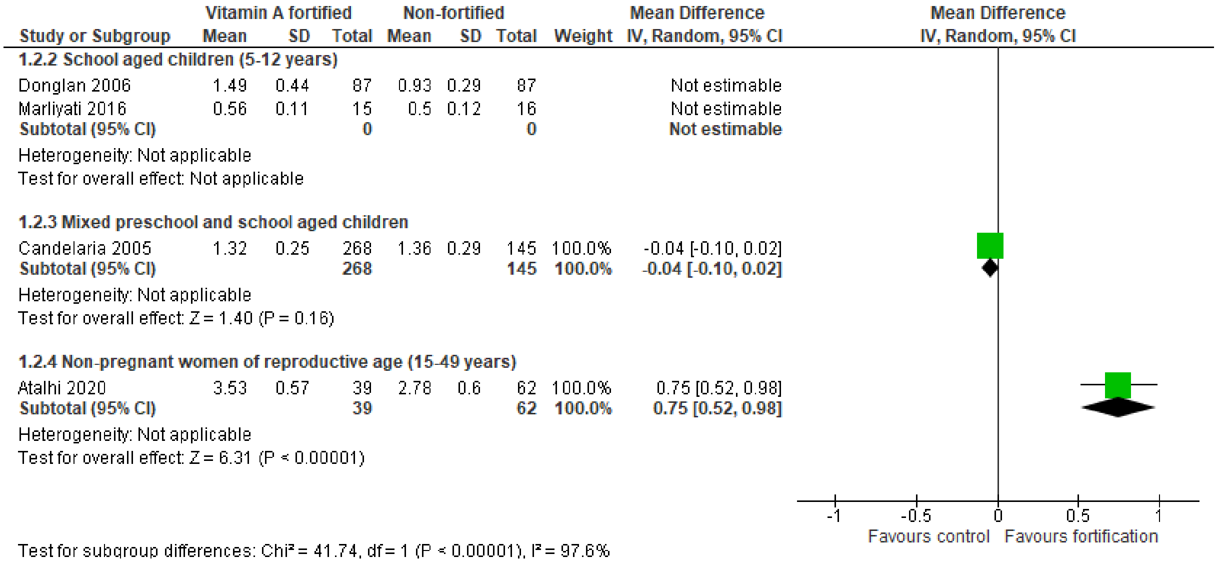

Test for subgroup differences: Chi² = 41.74, df = 1 (P < 0.00001), I² = 97.6%

Analysis 1.3. Vitamin A fortified versus non-fortified oils and fats. Outcome: Serum retinol by vitamin A intake in the population (as defined by inadequate vitamin A intakes by trialists)

	Vitamin A fortified			Non-fortified				Mean Difference
Study or Subgroup	Mean	SD	Total	Mean	SD	Total	Weight	IV, Random, 95% CI
**1.3.1 High risk of inadequate vitamin A intake in the population**								
Atalhi 2020	3.53	0.57	39	2.78	0.6	62	48.9%	0.75 [0.52, 0.98]
Candelaria 2005	1.32	0.25	268	1.36	0.29	145	51.1%	-0.04 [-0.10, 0.02]
Donglan 2006	1.49	0.44	87	0.93	0.29	87		Not estimable
**Subtotal (95% CI)**			**307**			**207**	**100.0%**	**0.35 [-0.43, 1.12]**
Heterogeneity: Tau² = 0.30; Chi² = 41.74, df = 1 (P < 0.00001); I² = 98%								
Test for overall effect: Z = 0.88 (P = 0.38)								
**1.3.2 Low risk of inadequate vitamin A intake in the population**								
Marliyati 2016	0.56	0.11	15	0.5	0.12	16		Not estimable
**Subtotal (95% CI)**			**0**			**0**		**Not estimable**
Heterogeneity: Not applicable								
Test for overall effect: Not applicable								
Test for subgroup differences: Not applicable								

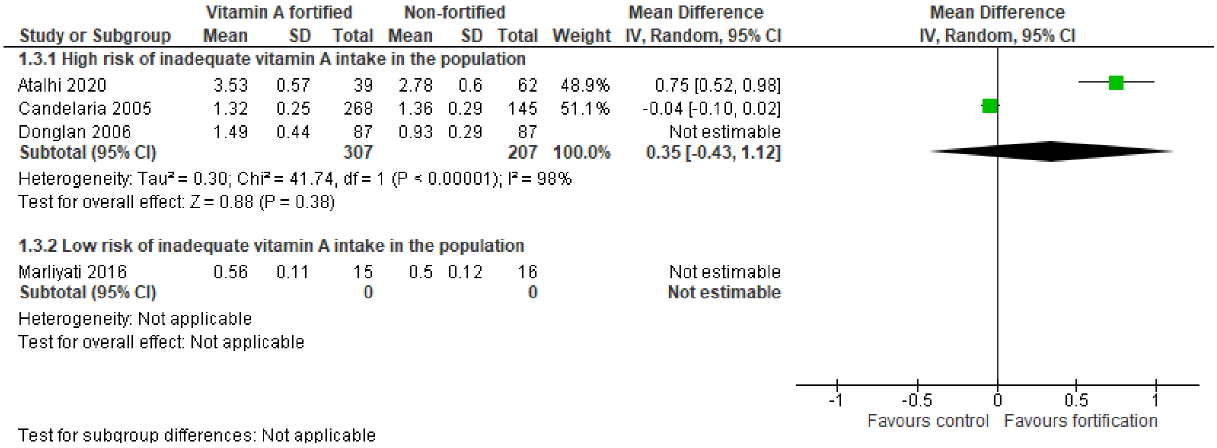

Analysis 1.4. Vitamin A fortified versus non-fortified oils and fats. Outcome: Serum retinol by public health significance

	Vitamin A fortified			Non-fortified				Mean Difference
Study or Subgroup	Mean	SD	Total	Mean	SD	Total	Weight	IV, Random, 95% CI
**1.4.1 Countries where vitamin A deficiency is a severe public health problem**								
Atalhi 2020	3.53	0.57	39	2.78	0.6	62	48.9%	0.75 [0.52, 0.98]
Candelaria 2005	1.32	0.25	268	1.36	0.29	145	51.1%	-0.04 [-0.10, 0.02]
**Subtotal (95% CI)**			**307**			**207**	**100.0%**	**0.35 [-0.43, 1.12]**
Heterogeneity: Tau² = 0.30; Chi² = 41.74, df = 1 (P < 0.00001); I² = 98%								
Test for overall effect: Z = 0.88 (P = 0.38)								
**1.4.2 Countries where vitamin A deficiency is a moderate public health problem**								
Marliyati 2016	0.56	0.11	15	0.5	0.12	16		Not estimable
**Subtotal (95% CI)**			**0**			**0**		**Not estimable**
Heterogeneity: Not applicable								
Test for overall effect: Not applicable								
**1.4.3 Countries where vitamin A deficiency is a mild public health problem**								
Donglan 2006	1.49	0.44	87	0.93	0.29	87		Not estimable
**Subtotal (95% CI)**			**0**			**0**		**Not estimable**
Heterogeneity: Not applicable								
Test for overall effect: Not applicable								
Test for subgroup differences: Not applicable								

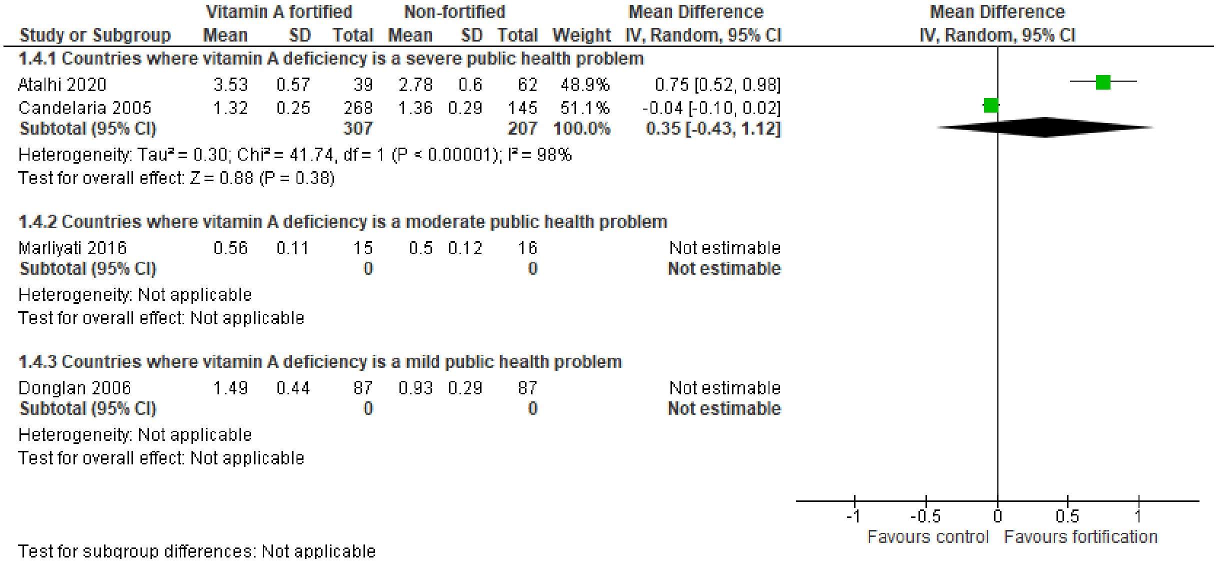

Analysis 1.5. Vitamin A fortified versus non-fortified oils and fats. Outcome: Serum retinol by exposure to additional vitamin A programmes

	Vitamin A fortified			Non-fortified				Mean Difference
Study or Subgroup	Mean	SD	Total	Mean	SD	Total	Weight	IV, Random, 95% CI
**1.5.1 One additional programme**								
Atalhi 2020	3.53	0.57	39	2.78	0.6	62	100.0%	0.75 [0.52, 0.98]
**Subtotal (95% CI)**			**39**			**62**	**100.0%**	**0.75 [0.52, 0.98]**
Heterogeneity: Not applicable								
Test for overall effect: Z = 6.31 (P < 0.00001)								
**1.5.3 Not mentioned**								
Candelaria 2005	1.32	0.25	268	1.36	0.29	145	100.0%	-0.04 [-0.10, 0.02]
Donglan 2006	1.49	0.44	87	0.93	0.29	87		Not estimable
Marliyati 2016	0.56	0.11	15	0.5	0.12	16		Not estimable
**Subtotal (95% CI)**			**268**			**145**	**100.0%**	**-0.04 [-0.10, 0.02]**
Heterogeneity: Not applicable								
Test for overall effect: Z = 1.40 (P = 0.16)								

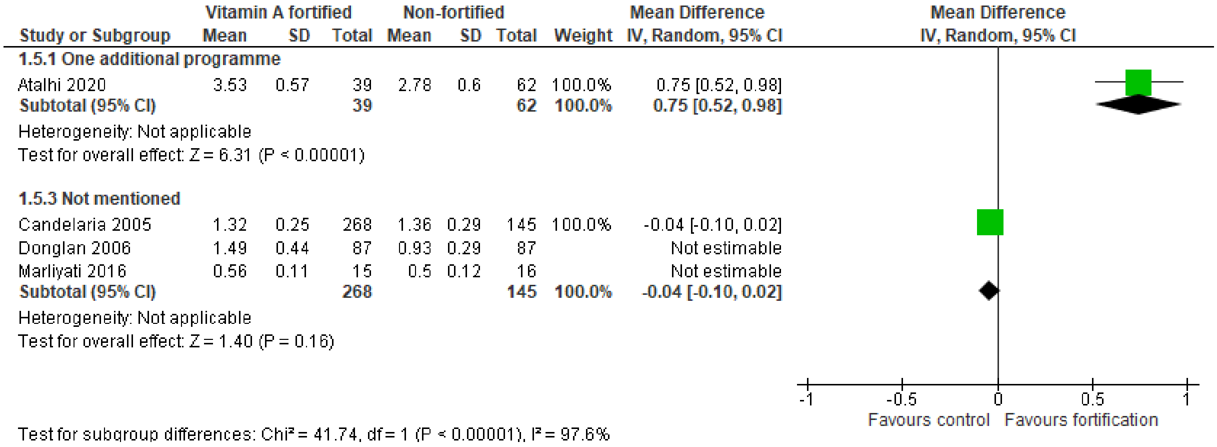

Test for subgroup differences: Chi² = 41.74, df = 1 (P < 0.00001), I² = 97.6%

Analysis 1.6. Vitamin A fortified versus non-fortified oils and fats. Outcome: Serum retinol by vehicle of the intervention (oils or fats)

	Vitamin A fortified			Non-fortified				Mean Difference
Study or Subgroup	Mean	SD	Total	Mean	SD	Total	Weight	IV, Random, 95% CI
**1.6.1 Oils**								
Atalhi 2020	3.53	0.57	39	2.78	0.6	62	48.9%	0.75 [0.52, 0.98]
Candelaria 2005	1.32	0.25	268	1.36	0.29	145	51.1%	-0.04 [-0.10, 0.02]
Donglan 2006	1.49	0.44	87	0.93	0.29	87		Not estimable
Marliyati 2016	0.56	0.11	15	0.5	0.12	16		Not estimable
**Subtotal (95% CI)**			**307**			**207**	**100.0%**	**0.35 [-0.43, 1.12]**
Heterogeneity: Tau² = 0.30; Chi² = 41.74, df = 1 (P < 0.00001); I² = 98%								
Test for overall effect: Z = 0.88 (P = 0.38)								
**1.6.2 Solid fats**								
**Subtotal (95% CI)**			**0**			**0**		**Not estimable**
Heterogeneity: Not applicable								
Test for overall effect: Not applicable								

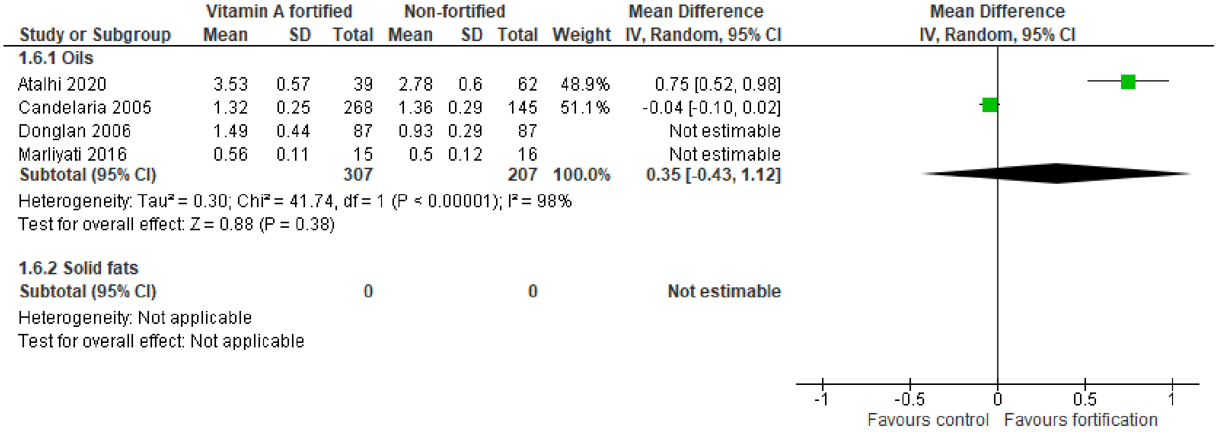

Analysis 1.7. Vitamin A fortified versus non-fortified oils and fats. Outcome: Serum retinol by consumption patterns

	Vitamin A fortification			Control				Mean Difference
Study or Subgroup	Mean	SD	Total	Mean	SD	Total	Weight	IV, Random, 95% CI
**1.7.1 Less than average consumption of their population**								
Marliyati 2016	0.56	0.11	15	0.5	0.12	16		Not estimable
**Subtotal (95% CI)**			**0**			**0**		**Not estimable**
Heterogeneity: Not applicable								
Test for overall effect: Not applicable								
**1.7.2 Not mentioned**								
Atalhi 2020	3.53	0.57	39	2.78	0.6	62	48.9%	0.75 [0.52, 0.98]
Candelaria 2005	1.32	0.25	268	1.36	0.29	145	51.1%	-0.04 [-0.10, 0.02]
Donglan 2006	1.49	0.44	87	0.93	0.29	87		Not estimable
**Subtotal (95% CI)**			**307**			**207**	**100.0%**	**0.35 [-0.43, 1.12]**
Heterogeneity: Tau² = 0.30; Chi² = 41.74, df = 1 (P < 0.00001); I² = 98%								
Test for overall effect: Z = 0.88 (P = 0.38)								

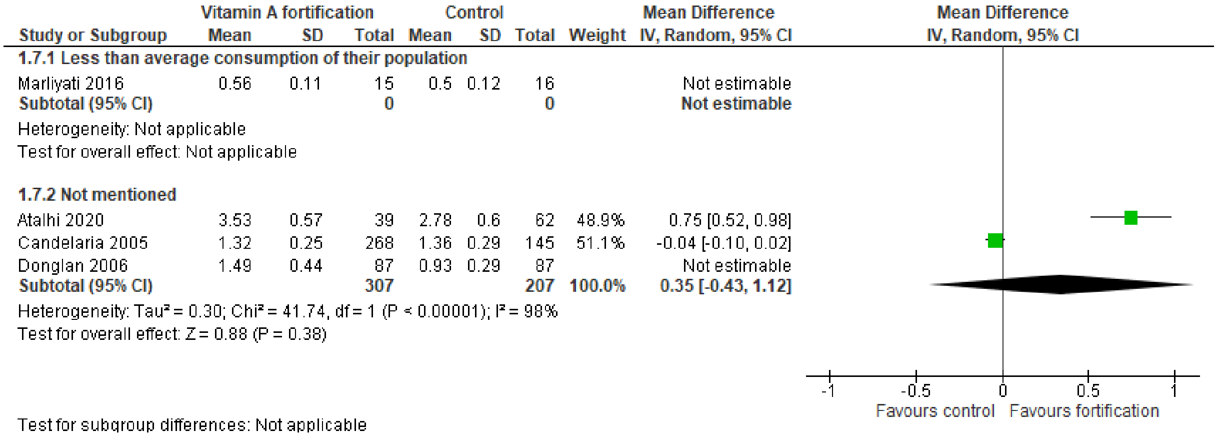

Test for subgroup differences: Not applicable

Analysis 1.8. Vitamin A fortified versus non-fortified oils and fats. Outcome: Serum retinol by intervention duration

Study or Subgroup	Vitamin A fortified Mean	SD	Total	Non-fortified Mean	SD	Total	Weight	Mean Difference IV, Random, 95% CI
**1.8.1 < six months**								
Donglan 2006	1.49	0.44	87	0.93	0.29	87		Not estimable
Marliyati 2016	0.56	0.11	15	0.5	0.12	16		Not estimable
**Subtotal (95% CI)**			**0**			**0**		**Not estimable**
Heterogeneity: Not applicable								
Test for overall effect: Not applicable								
**1.8.2 6 to 12 months**								
Atalhi 2020	3.53	0.57	39	2.78	0.6	62	48.9%	0.75 [0.52, 0.98]
Candelaria 2005	1.32	0.25	268	1.36	0.29	145	51.1%	-0.04 [-0.10, 0.02]
**Subtotal (95% CI)**			**307**			**207**	**100.0%**	**0.35 [-0.43, 1.12]**
Heterogeneity: Tau² = 0.30; Chi² = 41.74, df = 1 (P < 0.00001); I² = 98%								
Test for overall effect: Z = 0.88 (P = 0.38)								
**1.8.3 > 1 year**								
**Subtotal (95% CI)**			**0**			**0**		**Not estimable**
Heterogeneity: Not applicable								
Test for overall effect: Not applicable								
Test for subgroup differences: Not applicable								

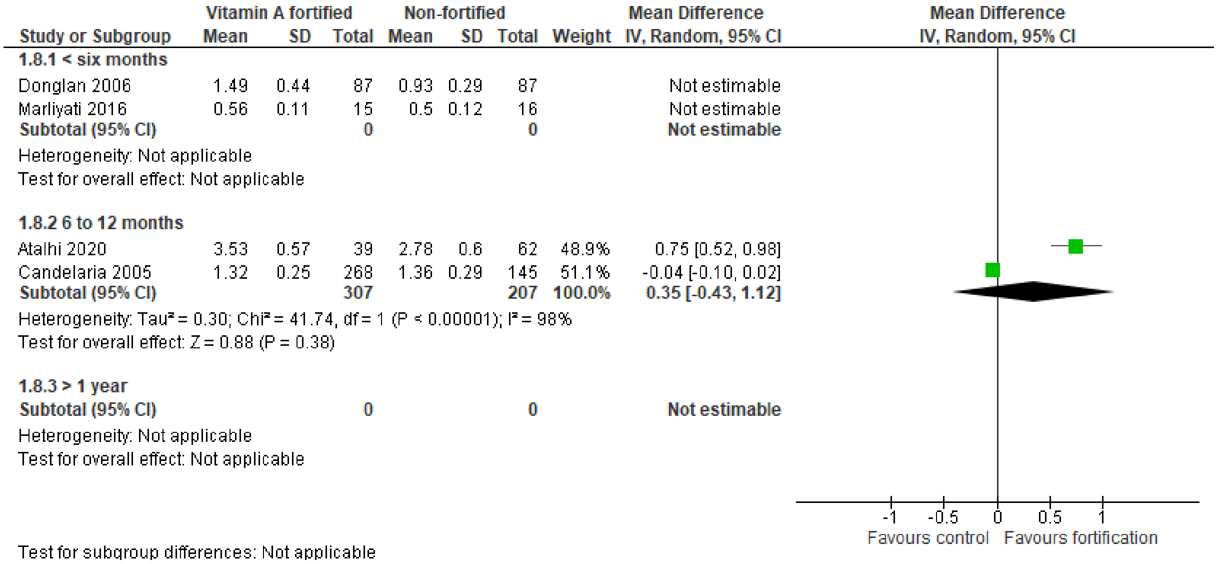

Analysis 1.9. Serum retinol by amount of vitamin A added through fortification

Study or Subgroup	Vitamin A fortified Mean	SD	Total	Non-fortified Mean	SD	Total	Weight	Mean Difference IV, Random, 95% CI
**1.9.1 More than 60 IU vitamin A per gram oil**								
Candelaria 2005	1.32	0.25	268	1.36	0.29	145	100.0%	-0.04 [-0.10, 0.02]
**Subtotal (95% CI)**			**268**			**145**	**100.0%**	**-0.04 [-0.10, 0.02]**
Heterogeneity: Not applicable								
Test for overall effect: Z = 1.40 (P = 0.16)								
**1.9.2 Less than 60 IU vitamin A per gram oil**								
Atalhi 2020	3.53	0.57	39	2.78	0.6	62	100.0%	0.75 [0.52, 0.98]
Donglan 2006	1.49	0.44	87	0.93	0.29	87		Not estimable
Marliyati 2016	0.56	0.11	15	0.5	0.12	16		Not estimable
**Subtotal (95% CI)**			**39**			**62**	**100.0%**	**0.75 [0.52, 0.98]**
Heterogeneity: Not applicable								
Test for overall effect: Z = 6.31 (P < 0.00001)								
Test for subgroup differences: Chi² = 41.74, df = 1 (P < 0.00001), I² = 97.6%								

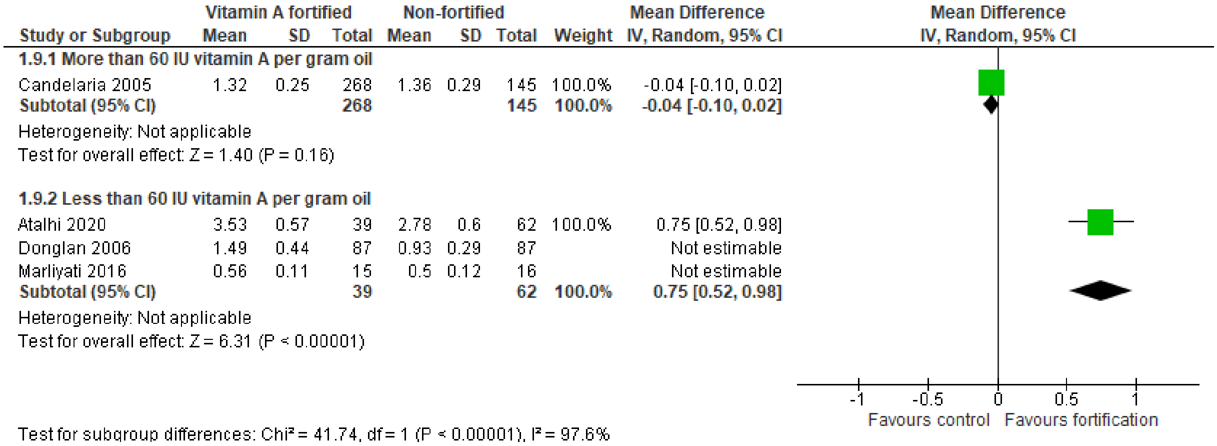

Analysis 1.10. Vitamin A fortified versus non-fortified oils and fats. Outcome: Serum retinol by type of vitamin A compound

Study or Subgroup	Vitamin A fortified Mean	SD	Total	Non-fortified Mean	SD	Total	Weight	Mean Difference IV, Random, 95% CI
**1.10.1 Retinyl acetate**								
**Subtotal (95% CI)**			**0**			**0**		**Not estimable**
Heterogeneity: Not applicable								
Test for overall effect: Not applicable								
**1.10.2 Retinyl palmitate**								
Atalhi 2020	3.53	0.57	39	2.78	0.6	62	100.0%	0.75 [0.52, 0.98]
**Subtotal (95% CI)**			**39**			**62**	**100.0%**	**0.75 [0.52, 0.98]**
Heterogeneity: Not applicable								
Test for overall effect: Z = 6.31 (P < 0.00001)								
**1.10.3 Provitamin A carotenoids**								
Marliyati 2016	0.56	0.11	15	0.5	0.12	16		Not estimable
**Subtotal (95% CI)**			**0**			**0**		**Not estimable**
Heterogeneity: Not applicable								
Test for overall effect: Not applicable								
**1.10.5 Not specified**								
Candelaria 2005	1.32	0.25	268	1.36	0.29	145	100.0%	-0.04 [-0.10, 0.02]
Donglan 2006	1.49	0.44	87	0.93	0.29	87		Not estimable
**Subtotal (95% CI)**			**268**			**145**	**100.0%**	**-0.04 [-0.10, 0.02]**
Heterogeneity: Not applicable								
Test for overall effect: Z = 1.40 (P = 0.16)								

Test for subgroup differences: Chi² = 41.74, df = 1 (P < 0.00001), I² = 97.6%

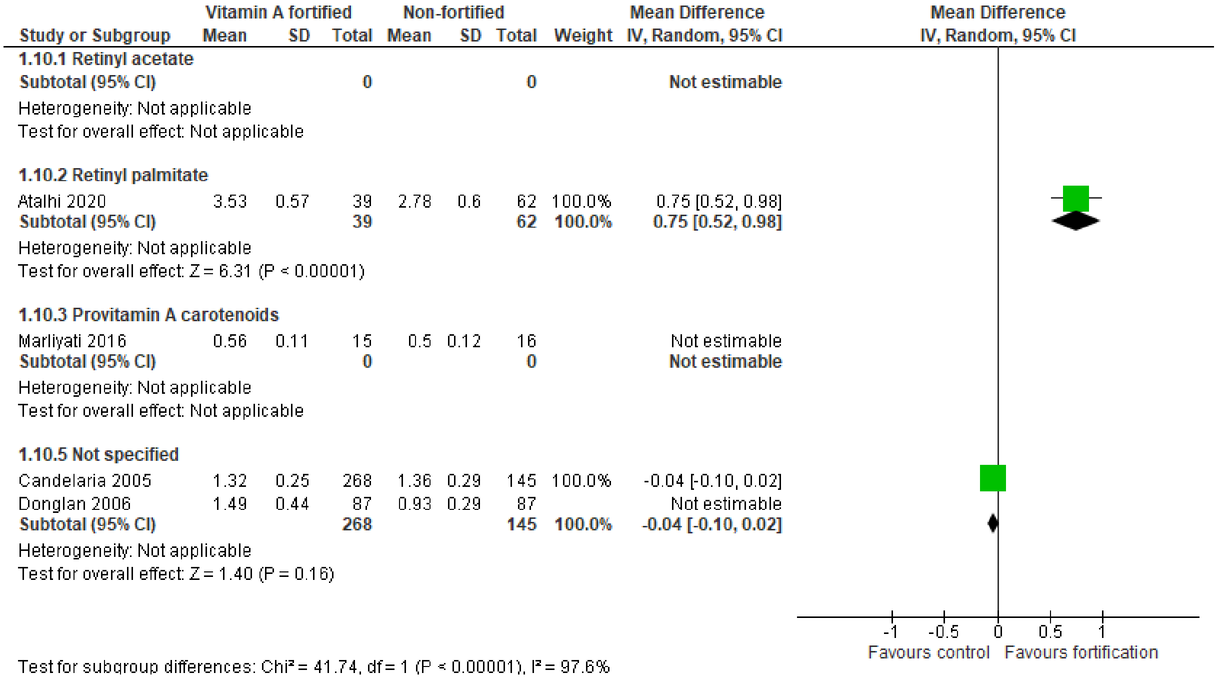

Analysis 1.11. Vitamin A fortified versus non-fortified oils and fats. Outcome: Serum retinol by type of vitamin A fortification intervention

Study or Subgroup	Vitamin A fortification Mean	SD	Total	Non-fortification Mean	SD	Total	Weight	Mean Difference IV, Random, 95% CI
**1.11.1 Mandatory fortification**								
**Subtotal (95% CI)**			**0**			**0**		**Not estimable**
Heterogeneity: Not applicable								
Test for overall effect: Not applicable								
**1.11.2 Market driven (voluntary) fortification**								
Atalhi 2020	3.53	0.57	39	2.78	0.6	62	48.9%	0.75 [0.52, 0.98]
Candelaria 2005	1.32	0.25	268	1.36	0.29	145	51.1%	-0.04 [-0.10, 0.02]
Donglan 2006	1.49	0.44	87	0.93	0.29	87		Not estimable
Marliyati 2016	0.56	0.11	15	0.5	0.12	16		Not estimable
**Subtotal (95% CI)**			**307**			**207**	**100.0%**	**0.35 [-0.43, 1.12]**
Heterogeneity: Tau² = 0.30; Chi² = 41.74, df = 1 (P < 0.00001); I² = 98%								
Test for overall effect: Z = 0.88 (P = 0.38)								
**Total (95% CI)**			**307**			**207**	**100.0%**	**0.35 [-0.43, 1.12]**
Heterogeneity: Tau² = 0.30; Chi² = 41.74, df = 1 (P < 0.00001); I² = 98%								
Test for overall effect: Z = 0.88 (P = 0.38)								
Test for subgroup differences: Not applicable								

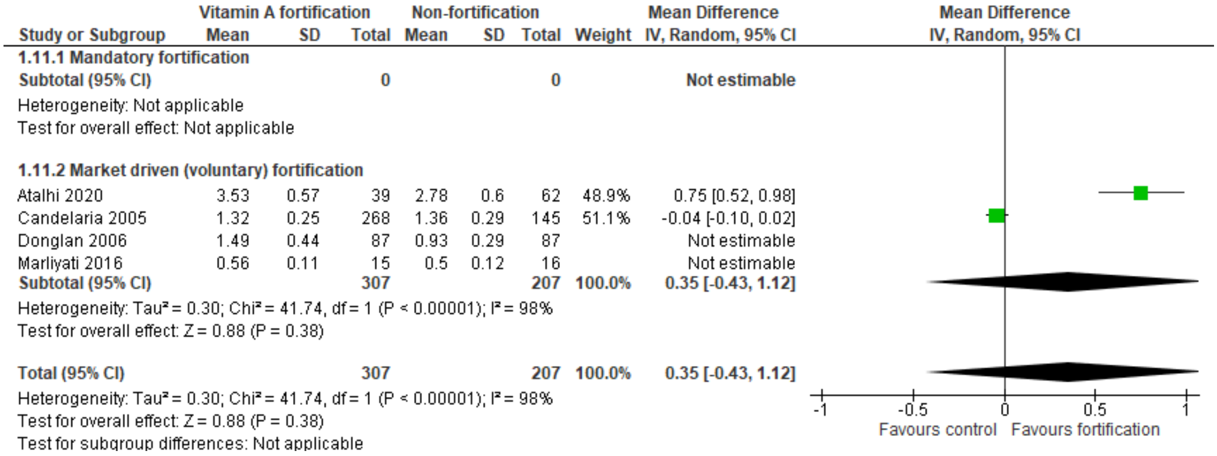

Analysis 1.12. Vitamin A fortified versus non-fortified oils and fats. Outcome: Serum retinol by method of cooking

Study or Subgroup	Vitamin A fortified Mean	SD	Total	Non-fortified Mean	SD	Total	Weight	Mean Difference IV, Random, 95% CI
**1.12.1 >50% of participants re-used cooking oil**								
Candelaria 2005	1.32	0.25	268	1.36	0.29	145	100.0%	-0.04 [-0.10, 0.02]
**Subtotal (95% CI)**			**268**			**145**	**100.0%**	**-0.04 [-0.10, 0.02]**
Heterogeneity: Not applicable								
Test for overall effect: Z = 1.40 (P = 0.16)								
**1.12.2 Not mentioned**								
Atalhi 2020	3.53	0.57	39	2.78	0.6	62	100.0%	0.75 [0.52, 0.98]
Donglan 2006	1.49	0.44	87	0.93	0.29	87		Not estimable
Marliyati 2016	0.56	0.11	15	0.5	0.12	16		Not estimable
**Subtotal (95% CI)**			**39**			**62**	**100.0%**	**0.75 [0.52, 0.98]**
Heterogeneity: Not applicable								
Test for overall effect: Z = 6.31 (P < 0.00001)								

Test for subgroup differences: Chi² = 41.74, df = 1 (P < 0.00001), I² = 97.6%

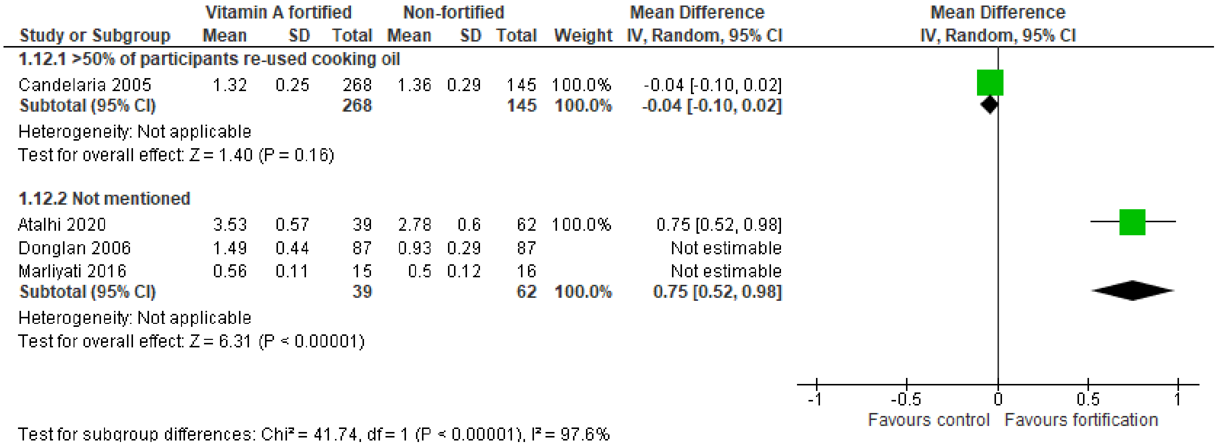

Analysis 1.13. Vitamin A fortified versus non-fortified oils and fats. Outcome: Serum retinol by delivery platform

Study or Subgroup	Vitamin A fortified Mean	SD	Total	Non-fortified Mean	SD	Total	Weight	Mean Difference IV, Random, 95% CI
**1.13.1 Social safety nets**								
Candelaria 2005	1.32	0.25	268	1.36	0.29	145	100.0%	-0.04 [-0.10, 0.02]
**Subtotal (95% CI)**			**268**			**145**	**100.0%**	**-0.04 [-0.10, 0.02]**
Heterogeneity: Not applicable								
Test for overall effect: Z = 1.40 (P = 0.16)								
**1.13.2 Not mentioned**								
Atalhi 2020	3.53	0.57	39	2.78	0.6	62	100.0%	0.75 [0.52, 0.98]
Donglan 2006	1.49	0.44	87	0.93	0.29	87		Not estimable
Marliyati 2016	0.56	0.11	15	0.5	0.12	16		Not estimable
**Subtotal (95% CI)**			**39**			**62**	**100.0%**	**0.75 [0.52, 0.98]**
Heterogeneity: Not applicable								
Test for overall effect: Z = 6.31 (P < 0.00001)								

Test for subgroup differences: Chi² = 41.74, df = 1 (P < 0.00001), I² = 97.6%

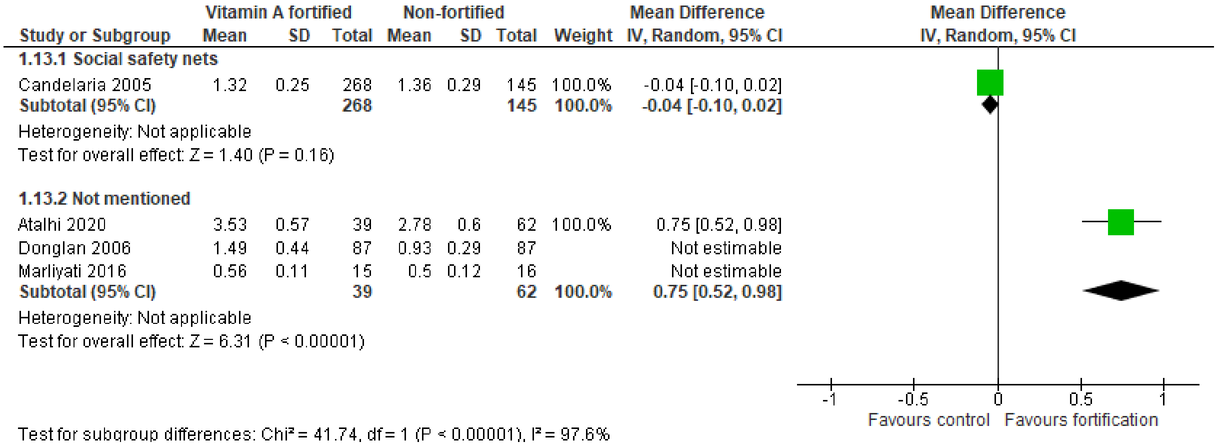

Analysis 1.14. Vitamin A fortified versus non-fortified oils and fats. Outcome: Subclinical vitamin A deficiency (serum retinol ≤ 0.70 μmol/L)

Study or Subgroup	Vitamin A fortified Events	Total	Non-fortified Events	Total	Risk Ratio M-H, Random, 95% CI
**1.14.1 RCTs**					
Candelaria 2005	0	268	0	144	Not estimable
**1.14.2 CCTs**					
Marliyati 2016	0	15	2	16	0.21 [0.01, 4.10]

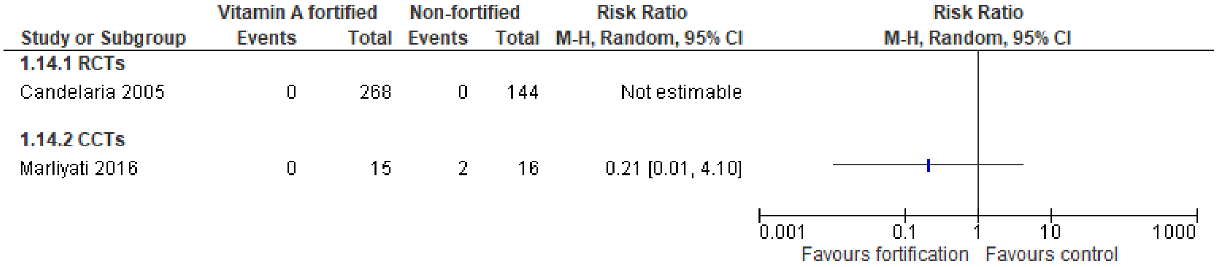

Analysis 1.15. Vitamin A fortified versus non-fortified oils and fats. Outcome: Dietary vitamin A intake

	Vitamin A fortified			Non-fortified			Mean Difference
Study or Subgroup	Mean	SD	Total	Mean	SD	Total	IV, Random, 95% CI
**1.15.1 RCTs**							
Candelaria 2005	936.8	719.9	268	952.5	161.2	144	-15.70 [-105.82, 74.42]
**1.15.2 CCTs**							
Marliyati 2016	464.2	73.8	15	223.6	109.9	16	240.60 [175.07, 306.13]

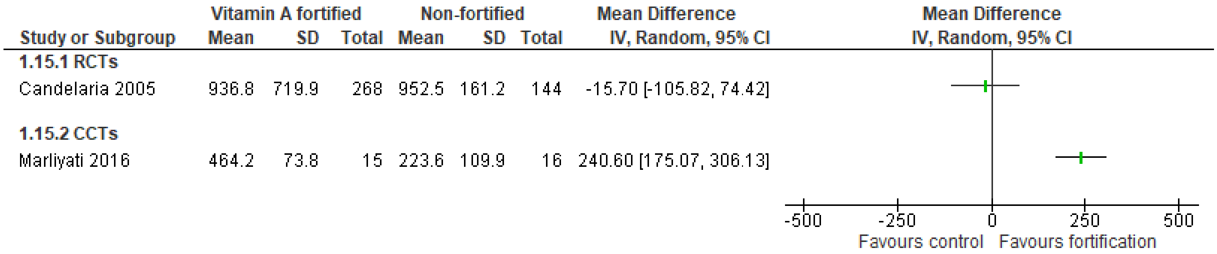

Analysis 1.16. Vitamin A fortified versus non-fortified oils and fats. Outcome: Retinol in breast milk (µmol/L)

	Vitamin A fortified			Non-fortified			Mean Difference
Study or Subgroup	Mean	SD	Total	Mean	SD	Total	IV, Random, 95% CI
Atalhi 2020	1.39	0.18	37	0.6	0.11	26	0.79 [0.72, 0.86]

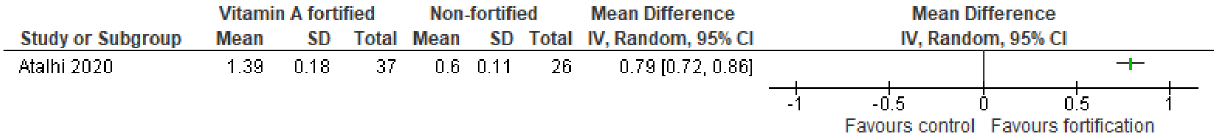

Analysis 1.17. Vitamin A fortified versus non-fortified oils and fats. Outcome: Mothers with low concentrations of retinol in breast milk (<1.05 µmol/L)

	Vitamin A fortified		Non-fortified		Risk Ratio
Study or Subgroup	Events	Total	Events	Total	M-H, Random, 95% CI
Atalhi 2020	2	62	39	39	0.04 [0.01, 0.14]

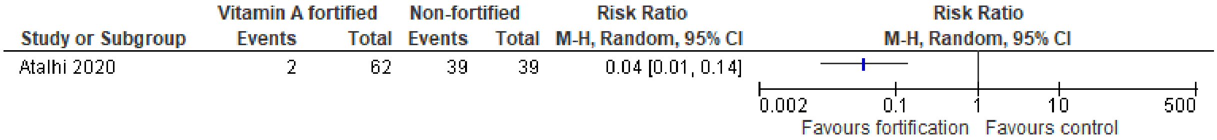

Analysis 1.18. Vitamin A fortified versus non-fortified oils and fats. Outcome: BMI (kg/m^2)

	Vitamin A fortified			Non-fortified			Mean Difference
Study or Subgroup	Mean	SD	Total	Mean	SD	Total	IV, Random, 95% CI
Marliyati 2016	15.19	0.84	15	14.5	0.85	16	0.69 [0.09, 1.29]

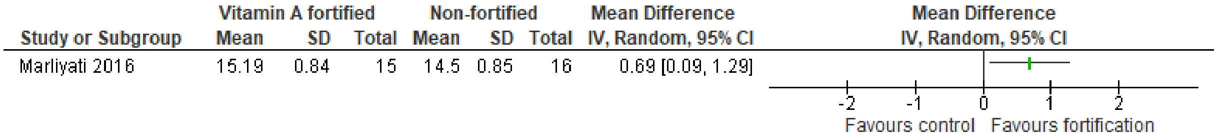

Analysis 1.19. Vitamin A fortified versus non-fortified oils and fats. Outcome: Weight-for-height z-scores

	Vitamin A fortified			Non-fortified			Mean Difference
Study or Subgroup	Mean	SD	Total	Mean	SD	Total	IV, Random, 95% CI
Candelaria 2005	-0.69	0.85	263	-0.67	0.95	145	-0.02 [-0.21, 0.17]

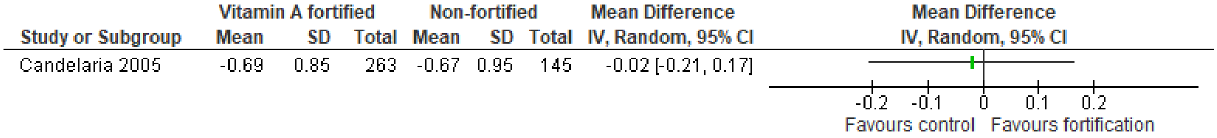

Analysis 1.20. Vitamin A fortified versus non-fortified oils and fats. Outcome: IgG levels (μmol/L)

Study or Subgroup	Vitamin A fortified Mean	SD	Total	Non-fortified Mean	SD	Total	Weight	Mean Difference IV, Random, 95% CI
Donglan 2006	60.04	24.08	40	58.71	21.81	39	61.1%	1.33 [-8.80, 11.46]
Marliyati 2016	19.77	11.88	15	18.66	22.82	16	38.9%	1.11 [-11.59, 13.81]
**Total (95% CI)**			**55**			**55**	**100.0%**	**1.24 [-6.67, 9.16]**

Heterogeneity: Tau² = 0.00; Chi² = 0.00, df = 1 (P = 0.98); I² = 0%
Test for overall effect: Z = 0.31 (P = 0.76)

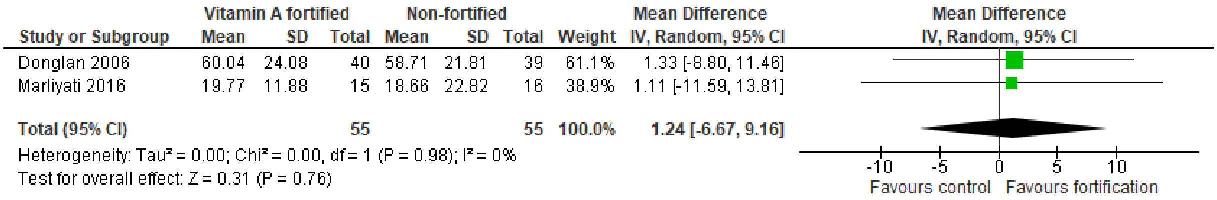

Analysis 1.21. Vitamin A fortified versus non-fortified oils and fats. Outcome: IgA levels (μmol/L)

Study or Subgroup	Vitamin A fortified Mean	SD	Total	Non-fortified Mean	SD	Total	Mean Difference IV, Random, 95% CI
Donglan 2006	8.56	3.25	40	6.63	3	40	1.93 [0.56, 3.30]

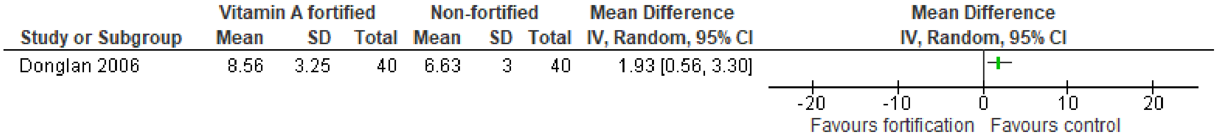

Analysis 1.22. Vitamin A fortified versus non-fortified oils and fats. Outcome: IgM levels (μmol/L)

Study or Subgroup	Vitamin A fortified Mean	SD	Total	Non-fortified Mean	SD	Total	Mean Difference IV, Random, 95% CI
Donglan 2006	0.96	0.45	40	0.99	0.43	40	-0.03 [-0.22, 0.16]

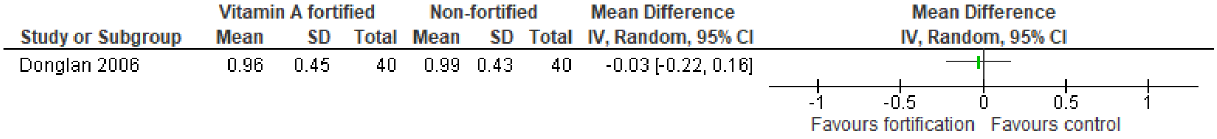

Analysis 1.23. Vitamin A fortified versus non-fortified oils and fats. Outcome: Type-2 diabetes mellitus

Study or Subgroup	Vitamin A fortified Events	Total	Non-fortified Events	Total	Risk Ratio M-H, Random, 95% CI
Keller 2017	1273	101178	1322	92625	0.88 [0.82, 0.95]

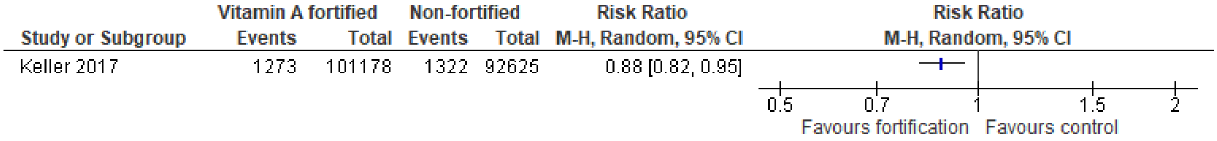

Supplement: Supplementary file 1 [file nutrients-15-05135-s001.zip › Supplementary File S5_VitaminA_results.pdf]
