# Supplementary material for: Benefits and Harms of Edible Vegetable Oils and Fats Fortified with Vitamins A and D as a Public Health Intervention in the General Population: A Systematic Review of Interventions
Source: Nutrients. 2023 Dec 18;15(24):5135. doi: 10.3390/nu15245135 (PMC10745565; doi:10.3390/nu15245135)

Supplementary file S6: Effect of Vitamin D fortified versus non-fortified oils and fats

Analysis 2.1. Vitamin D fortified versus non-fortified oils and fats. Outcome: Serum 25-hydroxy vitamin D

	Vitamin D fortified			Non-fortified			Mean Difference
Study or Subgroup	Mean	SD	Total	Mean	SD	Total	IV, Random, 95% CI
Ghasemifard 2022	83.99	25.66	30	77.4	28.48	32	6.59 [-6.89, 20.07]

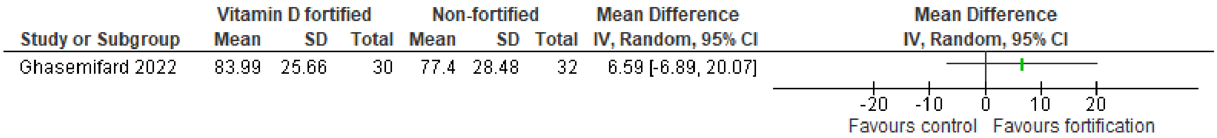

Analysis 2.2. Vitamin D fortified versus non-fortified oils and fats. Outcome: Serum 25-hydroxy vitamin D by vitamin D intake in the population

	Vitamin D fortified			Non-fortified				Mean Difference
Study or Subgroup	Mean	SD	Total	Mean	SD	Total	Weight	IV, Random, 95% CI
**2.2.1 Vitamin D deficient**								
Ghasemifard 2022_vitamin D deficient subgroup	60.28	21.39	12	54.29	15.08	14	41.2%	5.99 [-8.46, 20.44]
**Subtotal (95% CI)**			**12**			**14**	**41.2%**	**5.99 [-8.46, 20.44]**
Heterogeneity: Not applicable								
Test for overall effect: Z = 0.81 (P = 0.42)								
**2.2.2 Vitamin D sufficient**								
Ghasemifard 2022_vitamin D sufficient subgroup	99.81	12.85	18	95.4	22.84	18	58.8%	4.41 [-7.70, 16.52]
**Subtotal (95% CI)**			**18**			**18**	**58.8%**	**4.41 [-7.70, 16.52]**
Heterogeneity: Not applicable								
Test for overall effect: Z = 0.71 (P = 0.48)								
**Total (95% CI)**			**30**			**32**	**100.0%**	**5.06 [-4.22, 14.34]**
Heterogeneity: Tau² = 0.00; Chi² = 0.03, df = 1 (P = 0.87); I² = 0%								
Test for overall effect: Z = 1.07 (P = 0.29)								
Test for subgroup differences: Chi² = 0.03, df = 1 (P = 0.87), I² = 0%								

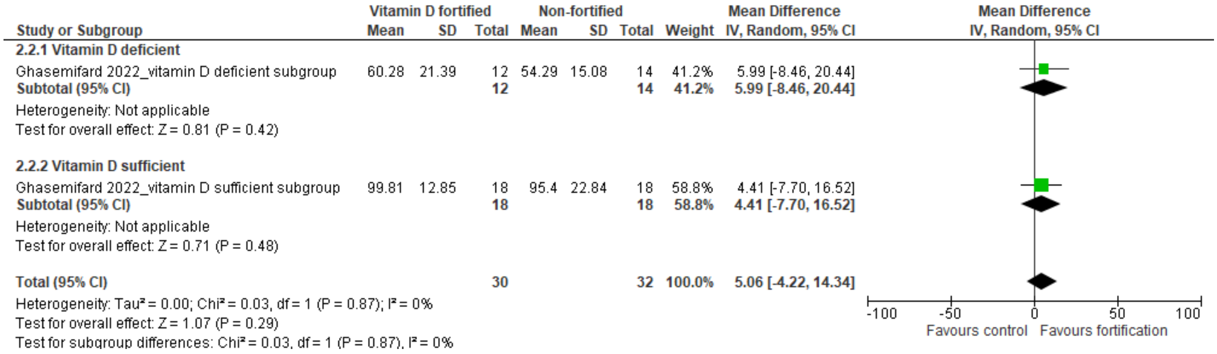

Analysis 2.3. Vitamin D fortified versus non-fortified oils and fats. Outcome: Parathyroid hormone (PTH), pmol/L

	Vitamin D fortified			Non-fortified			Mean Difference
Study or Subgroup	Mean	SD	Total	Mean	SD	Total	IV, Random, 95% CI
Ghasemifard 2022	2.89	1.19	18	2.79	2.04	18	0.10 [-0.99, 1.19]

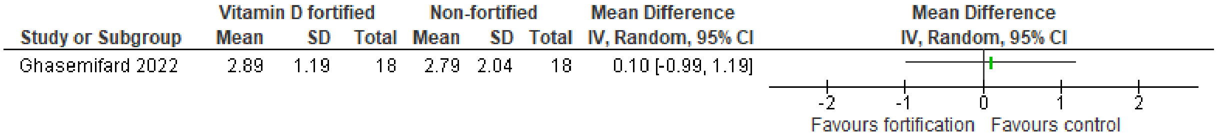

Analysis 2.4. Vitamin D fortified versus non-fortified oils and fats. Outcome: Vitamin D intake, mcg

	Vitamin D fortified			Non-fortified			Mean Difference
Study or Subgroup	Mean	SD	Total	Mean	SD	Total	IV, Random, 95% CI
Ghasemifard 2022	0.81	1.37	30	23.16	88.09	32	-22.35 [-52.88, 8.18]

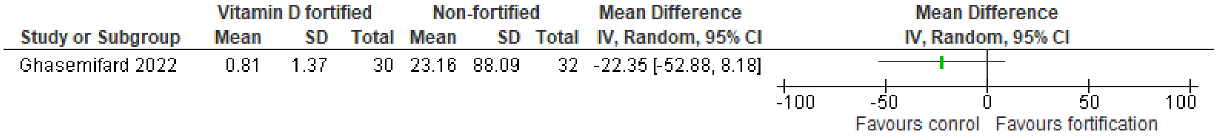

Analysis 2.5. Vitamin D fortified versus non-fortified oils and fats. Outcome: Bone alkaline phosphatase (B-ALP), IU/L

Study or Subgroup	Vitamin D fortified Mean	SD	Total	Non-fortified Mean	SD	Total	Mean Difference IV, Random, 95% CI
Ghasemifard 2022	46.92	8.15	18	41.16	9.78	18	5.76 [-0.12, 11.64]

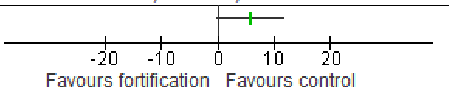

Analysis 2.6. Vitamin D fortified versus non-fortified oils and fats. Outcome: Total cholesterol (mmol/L)

Study or Subgroup	Vitamin D fortified Mean	SD	Total	Non-fortified Mean	SD	Total	Weight	Mean Difference IV, Random, 95% CI
Ghasemifard 2022	3.89	0.85	30	3.88	0.72	32	100.0%	0.01 [-0.38, 0.40]
**Total (95% CI)**			**30**			**32**	**100.0%**	**0.01 [-0.38, 0.40]**

Heterogeneity: Not applicable
Test for overall effect: Z = 0.05 (P = 0.96)

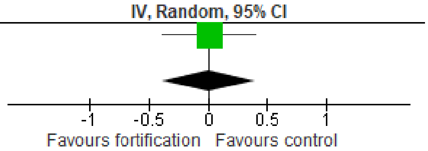

Analysis 2.7. Vitamin D fortified versus non-fortified oils and fats. Outcome: LDL cholesterol (mmol/L)

Study or Subgroup	Vitamin D fortified Mean	SD	Total	Non-fortified Mean	SD	Total	Weight	Mean Difference IV, Random, 95% CI
Ghasemifard 2022	2.2	0.6	30	2.18	0.49	32	100.0%	0.02 [-0.25, 0.29]
**Total (95% CI)**			**30**			**32**	**100.0%**	**0.02 [-0.25, 0.29]**

Heterogeneity: Not applicable
Test for overall effect: Z = 0.14 (P = 0.89)

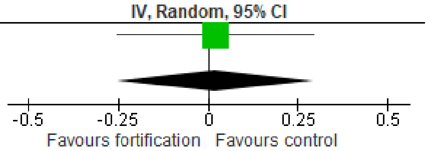

Analysis 2.8. Vitamin D fortified versus non-fortified oils and fats. Outcome: HDL cholesterol (mmol/L)

Study or Subgroup	Vitamin D fortified Mean	SD	Total	Non-fortified Mean	SD	Total	Weight	Mean Difference IV, Random, 95% CI
Ghasemifard 2022	1.16	0.18	30	1.13	0.23	32	100.0%	0.03 [-0.07, 0.13]
**Total (95% CI)**			**30**			**32**	**100.0%**	**0.03 [-0.07, 0.13]**

Heterogeneity: Not applicable
Test for overall effect: Z = 0.57 (P = 0.57)

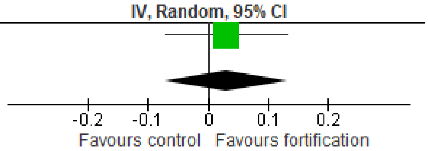

Analysis 2.9. Vitamin D fortified versus non-fortified oils and fats. Outcome: Triglyceride (mmol/L)

Study or Subgroup	Vitamin D fortified Mean	SD	Total	Non-fortified Mean	SD	Total	Mean Difference IV, Random, 95% CI
Ghasemifard 2022	1.31	0.75	30	1.29	0.7	32	0.02 [-0.34, 0.38]

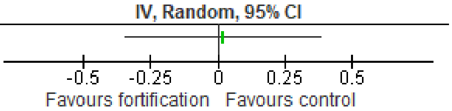

Analysis 2.10. Vitamin D fortified versus non-fortified oils and fats. Outcome: Gestational hypertension

Study or Subgroup	Vitamin D fortified Events	Vitamin D fortified Total	Non-fortified Events	Non-fortified Total	Risk Ratio M-H, Random, 95% CI
Stougaard 2018	399	35124	396	38113	1.09 [0.95, 1.26]

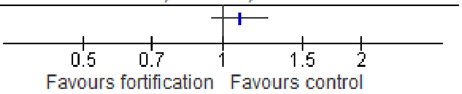

Analysis 2.11. Vitamin D fortified versus non-fortified oils and fats. Outcome: Preeclampsia

Study or Subgroup	Vitamin D fortified Events	Vitamin D fortified Total	Non-fortified Events	Non-fortified Total	Risk Ratio M-H, Random, 95% CI
Stougaard 2018	1310	35124	1370	38113	1.04 [0.96, 1.12]

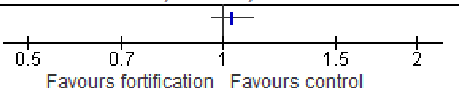

Analysis 2.12. Vitamin D fortified versus non-fortified oils and fats. Outcome: Eclampsia

Study or Subgroup	Vitamin D fortified Events	Vitamin D fortified Total	Non-fortified Events	Non-fortified Total	Risk Ratio M-H, Random, 95% CI
Stougaard 2018	267	35124	295	38113	0.98 [0.83, 1.16]

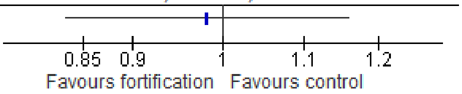

Analysis 2.13. Vitamin D fortified versus non-fortified oils and fats. Outcome: Gestational diabetes mellitus

Study or Subgroup	Vitamin D fortified Events	Vitamin D fortified Total	Non-fortified Events	Non-fortified Total	Risk Ratio M-H, Random, 95% CI
Keller 2018	297	14016	361	14855	0.87 [0.75, 1.01]

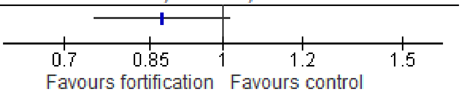

Analysis 2.14. Vitamin D fortified versus non-fortified oils and fats. Outcome: Childhood asthma

Study or Subgroup	Vitamin D fortified Events	Vitamin D fortified Total	Non-fortified Events	Non-fortified Total	Risk Ratio M-H, Random, 95% CI
Keller 2018	1427	106347	1613	115900	0.96 [0.90, 1.03]

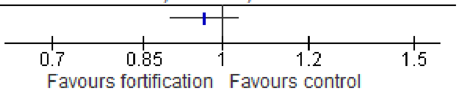

Analysis 2.15. Vitamin D fortified versus non-fortified oils and fats. Outcome: Type-1 diabetes mellitus

	Vitamin D fortified		Non-fortified		Risk Ratio
Study or Subgroup	Events	Total	Events	Total	M-H, Random, 95% CI
Jacobsen 2015	298	127207	404	134749	0.78 [0.67, 0.91]

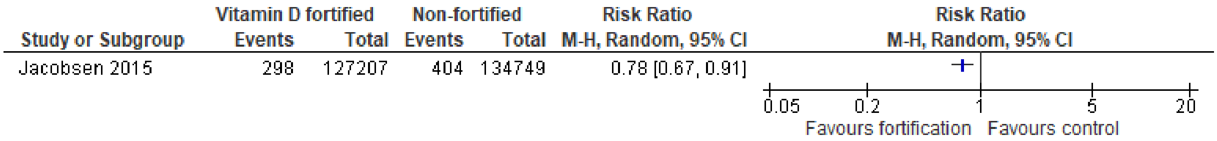

Analysis 2.16. Vitamin D fortified versus non-fortified oils and fats. Outcome: Fractures

	Vitamin D fortified		Non-fortified		Risk Ratio
Study or Subgroup	Events	Total	Events	Total	M-H, Random, 95% CI
Jacobsen 2015	12330	104406	16058	113577	0.84 [0.82, 0.85]

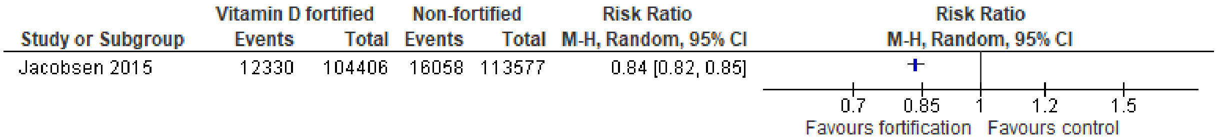

Analysis 2.17. Vitamin D fortified versus non-fortified oils and fats. Outcome: Birth weight (g)

	Vitamin D fortified			Non-fortified			Mean Difference
Study or Subgroup	Mean	SD	Total	Mean	SD	Total	IV, Random, 95% CI
Jensen 2014	3,324	549	4836	3,367	549	5716	-43.00 [-64.02, -21.98]

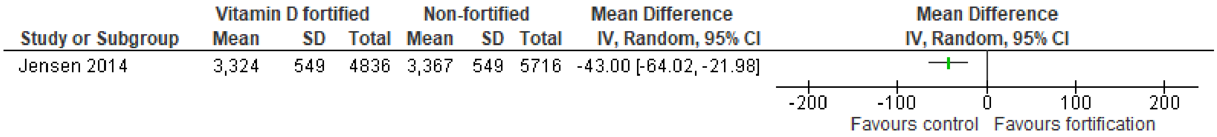

Analysis 2.18. Vitamin D fortified versus non-fortified oils and fats. Outcome: Low birth weight (<2500 g)

	Vitamin D fortified		Non-fortified		Risk Ratio
Study or Subgroup	Events	Total	Events	Total	M-H, Random, 95% CI
Jensen 2014	301	4836	308	5716	1.16 [0.99, 1.35]

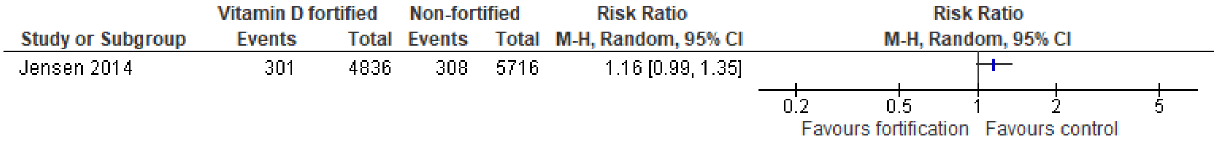

Analysis 2.19. Vitamin D fortified versus non-fortified oils and fats. Outcome: High birth weight (>4000 g)

	Vitamin D fortified		Non-fortified		Risk Ratio
Study or Subgroup	Events	Total	Events	Total	M-H, Random, 95% CI
Jensen 2014	407	4836	583	5716	0.83 [0.73, 0.93]

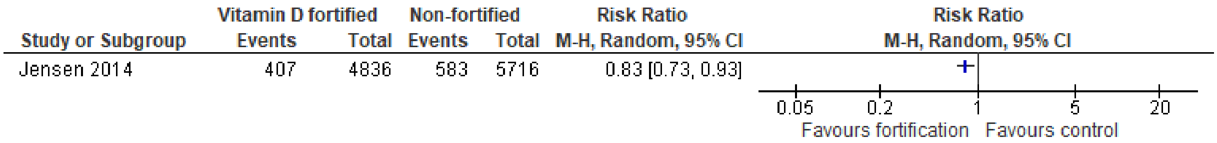

Analysis 2.20. Vitamin D fortified versus non-fortified oils and fats. Outcome: BMI (at 7 years)

	Vitamin D fortified			Non-fortified			Mean Difference
Study or Subgroup	Mean	SD	Total	Mean	SD	Total	IV, Random, 95% CI
Jensen 2014	15.9	1.7	5029	16	1.8	5803	-0.10 [-0.17, -0.03]

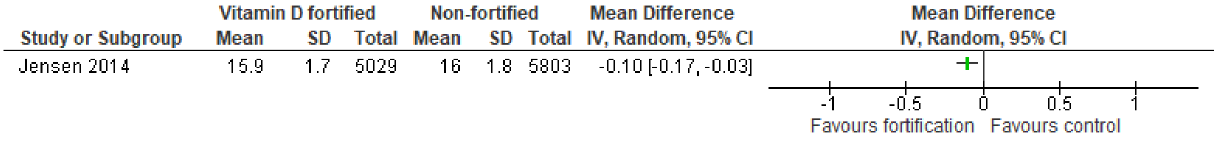

Analysis 2.21. Vitamin D fortified versus non-fortified oils and fats. Outcome: BMI z-scores

Study or Subgroup	Vitamin D fortified Mean	SD	Total	Non-fortified Mean	SD	Total	Mean Difference IV, Random, 95% CI
Jensen 2014	0.3	1.1	5029	0.3	1.2	5803	0.00 [-0.04, 0.04]

Mean Difference
IV, Random, 95% CI

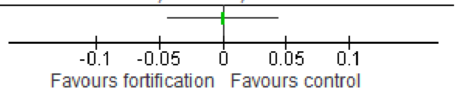

Analysis 2.22. Vitamin D fortified versus non-fortified oils and fats. Outcome: Overweight

Study or Subgroup	Vitamin D fortified Events	Total	Non-fortified Events	Total	Risk Ratio M-H, Random, 95% CI
Jensen 2014	1172	5029	1469	5803	0.92 [0.86, 0.98]

Risk Ratio
M-H, Random, 95% CI

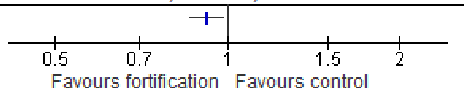

Analysis 2.23. Vitamin D fortified versus non-fortified oils and fats. Outcome: Obesity

Study or Subgroup	Vitamin D fortified Events	Total	Non-fortified Events	Total	Risk Ratio M-H, Random, 95% CI
Jensen 2014	523	5029	708	5803	0.85 [0.77, 0.95]

Risk Ratio
M-H, Random, 95% CI

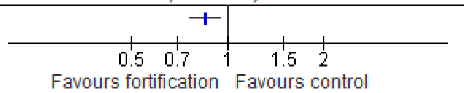

Analysis 2.24. Vitamin D fortified versus non-fortified oils and fats. Outcome: IBD incidence

Study or Subgroup	Vitamin D fortified Events	Total	Non-fortified Events	Total	Risk Ratio M-H, Random, 95% CI
Moos 2020	875	98856	1102	108044	0.87 [0.79, 0.95]

Risk Ratio
M-H, Random, 95% CI

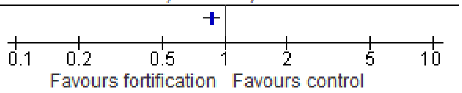

Analysis 2.25. Vitamin D fortified versus non-fortified oils and fats. Outcome: Coeliac disease

Study or Subgroup	Vitamin D fortified Events	Total	Non-fortified Events	Total	Risk Ratio M-H, Random, 95% CI
Moos 2020	148	98856	199	108044	0.81 [0.66, 1.01]

Risk Ratio
M-H, Random, 95% CI

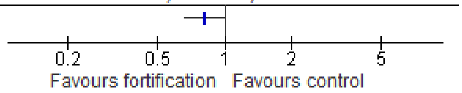

Analysis 2.26. Vitamin D fortified versus non-fortified oils and fats. Outcome: Energy intake (kcal)

Study or Subgroup	Vitamin D fortified Mean	SD	Total	Non-fortified Mean	SD	Total	Mean Difference IV, Random, 95% CI
Ghasemifard 2022	1,364.2	465.32	30	1,644.2	471.78	32	-280.00 [-513.33, -46.67]

Mean Difference
IV, Random, 95% CI

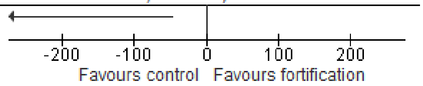

Supplement: Supplementary file 1 [file nutrients-15-05135-s001.zip › Supplementary File S6_VitaminD_results.pdf]
