# Supplementary material for: Benefits and Harms of Edible Vegetable Oils and Fats Fortified with Vitamins A and D as a Public Health Intervention in the General Population: A Systematic Review of Interventions
Source: Nutrients. 2023 Dec 18;15(24):5135. doi: 10.3390/nu15245135 (PMC10745565; doi:10.3390/nu15245135)
Supplement: Supplementary file 1 [file nutrients-15-05135-s001.zip › Supplementary File S7_Grade_tables.pdf]

Supplementary file S7: Grade assessment

Table 1. GRADE Assessment – Vitamin D fortification vs. no fortification with vitamin D (Evidence profile table for the primary outcomes)

Certainty assessment							№ of patients		Effect		Certainty	Importance
№ of studies	Study design	Risk of bias	Inconsistency	Indirectness	Imprecision	Other considerations	vitamin D fortified oils or fats	same oils or fats without vitamin D	Relative (95% CI)	Absolute (95% CI)		
**Serum 25-hydroxy vitamin D concentrations (µmol/L) (follow-up: 12 weeks)**												
1[1]	randomised trials	not serious	not serious[a]	not serious	very serious[b]	none	30	32	-	MD **6.59 nmol/L higher** (6.89 lower to 20.07 higher)	⊕⊕◯◯ Low	CRITICAL
**Vitamin D deficiency - not measured**												
-	-	-	-	-	-	-	-	-	-	-	-	CRITICAL
**Osteomalacia in elderly - not measured**												
-	-	-	-	-	-	-	-	-	-	-	-	CRITICAL
**Nutritional rickets - not measured**												
-	-	-	-	-	-	-	-	-	-	-	-	CRITICAL
**Adverse effects (hypervitaminosis, hypercalcemia, kidney stones) - not measured**												

Certainty assessment							№ of patients		Effect		Certainty	Importance
№ of studies	Study design	Risk of bias	Inconsistency	Indirectness	Imprecision	Other considerations	vitamin D fortified oils or fats	same oils or fats without vitamin D	Relative (95% CI)	Absolute (95% CI)		
-	-	-	-	-	-	-	-	-	-	-	-	CRITICAL
**Morbidity - not measured**												
-	-	-	-	-	-	-	-	-	-	-	-	CRITICAL
**Mortality - not measured**												
-	-	-	-	-	-	-	-	-	-	-	-	CRITICAL

CI: confidence interval; **MD:** mean difference; **RR:** risk ratio

Explanations

a. This is a single study so inconsistency cannot be judged.

b. Downgraded by two levels for imprecision since results are derived from one study, where total sample size was very low (n<100).

References

1.Ghasemifard, N., Hassanzadeh-Rostami, Z., Abbasi, A., Naghavi, A. M., Faghih, S.. Effects of vitamin D-fortified oil intake versus vitamin D supplementation on vitamin D status and bone turnover factors: a double blind randomized clinical trial. Clinical nutrition ESPEN; 2022.

Table 2. GRADE Assessment – Vitamin A fortification vs. no fortification with vitamin A – Secondary outcomes

Certainty assessment							№ of patients		Effect		Certainty	Importance
№ of studies	Study design	Risk of bias	Inconsistency	Indirectness	Imprecision	Other considerations	vitamin A fortified oils or fats	same oils or fats without vitamin A	Relative (95% CI)	Absolute (95% CI)		
**Dietary vitamin A intake (µg RE/day) - Randomized Studies (follow-up: 6 months)**												
1[1]	randomised trials	serious[b]	not serious[e]	not serious	serious[c]	none	268	144	-	MD **15.7 µg RE/day lower** (105.82 lower to 74.42 higher)	⊕⊕◯◯ Low	IMPORTANT
**Dietary vitamin A intake (µg RE/day) - Non-randomized Studies (follow-up: 2 months)**												
1[3]	controlled clinical trials	serious[b]	not serious[e]	not serious	very serious[d]	none	15	16	-	MD **240.6 µg RE/day higher** (175.07 higher to 306.13 higher)	⊕◯◯◯ Very low	IMPORTANT
**Liver stores of vitamin A - not measured**												
-	-	-	-	-	-	-	-	-	-	-	-	IMPORTANT
**Retinol in breast milk (µmol/L) (follow-up: 6 months)**												

Certainty assessment							№ of patients		Effect		Certainty	Importance
№ of studies	Study design	Risk of bias	Inconsistency	Indirectness	Imprecision	Other considerations	vitamin A fortified oils or fats	same oils or fats without vitamin A	Relative (95% CI)	Absolute (95% CI)		
1[2]	randomised trials	serious[b]	not serious[e]	not serious	very serious[d]	none	37	26	-	MD **0.79 µmol/L higher** (0.72 higher to 0.86 higher)	⊕◯◯◯ Very low	IMPORTANT
**Mothers with low concentrations of retinol in breast milk (<1.05 µmol/L) (follow-up: 6 months)**												
1[2]	randomised trials	serious[b]	not serious[e]	not serious	very serious[d]	none	2/62 (3.2%)	39/39 (100.0%)	**RR 0.04** (0.01 to 0.14)	**960 fewer per 1 000** (from 990 fewer to 860 fewer)	⊕◯◯◯ Very low	IMPORTANT
**Prevalence of biochemical vitamin A deficiency (measured by Modified Relative Dose Response test; MRDR) - not measured**												
-	-	-	-	-	-	-	-	-	-	-	-	IMPORTANT
**Retinol-binding protein level - not measured**												
-	-	-	-	-	-	-	-	-	-	-	-	IMPORTANT
**Beta-carotene levels - not measured**												
-	-	-	-	-	-	-	-	-	-	-	-	IMPORTANT
**Serum ferritin - not measured**												

Certainty assessment							№ of patients		Effect		Certainty	Importance
№ of studies	Study design	Risk of bias	Inconsistency	Indirectness	Imprecision	Other considerations	vitamin A fortified oils or fats	same oils or fats without vitamin A	Relative (95% CI)	Absolute (95% CI)		
-	-	-	-	-	-	-	-	-	-	-	-	IMPORTANT
**Anaemia - not measured**												
-	-	-	-	-	-	-	-	-	-	-	-	IMPORTANT
**BMI - Non-randomized Studies (follow-up: 2 months)**												
1[3]	randomised trials	serious[b]	not serious[e]	not serious	very serious[d]	none	15	16	-	MD **0.69 kg/m2 higher** (0.09 higher to 1.29 higher)	⊕◯◯◯ Very low	
**Weight-for-height (in z-scores) (follow-up: 6 months)**												
1[1]	randomised trials	serious[b]	not serious[e]	not serious	serious[f]	none	268	145	-	MD **0.02 z-scores lower** (0.21 lower to 0.17 higher)	⊕⊕◯◯ Low	
**IgG levels (μmol/L) - Non-randomized Studies (follow-up: range 2 to 5 months)**												

Certainty assessment							№ of patients		Effect		Certainty	Importance
№ of studies	Study design	Risk of bias	Inconsistency	Indirectness	Imprecision	Other considerations	vitamin A fortified oils or fats	same oils or fats without vitamin A	Relative (95% CI)	Absolute (95% CI)		
2[3,4]	controlled clinical trials	serious[a]	not serious	not serious	serious[g]	none	55	55	-	MD **1.24 µmol/L higher** (6.67 lower to 9.16 higher)	⊕⊕◯◯ Low	
**IgA levels (µmol/L) - Non-randomized Studies (follow-up: 5 months)**												
1[4]	controlled clinical trials	very serious[h]	not serious	not serious	serious[d]	none	40	40	-	MD **1.93 µmol/l higher** (0.56 higher to 3.3 higher)	⊕◯◯◯ Very low	
**IgM levels (µmol/L) - Non-randomized Studies (follow-up: 5 months)**												
1[4]	controlled clinical trials	very serious[h]	not serious	not serious	serious[d]	none	40	40	-	MD **0.03 µmol/L lower** (0.22 lower to 0.16 higher)	⊕◯◯◯ Very low	
**CRP - not reported**												
-	-	-	-	-	-	-	-	-	-	-	-	

Certainty assessment							№ of patients		Effect		Certainty	Importance
**№ of studies**	**Study design**	**Risk of bias**	**Inconsistency**	**Indirectness**	**Imprecision**	**Other considerations**	**vitamin A fortified oils or fats**	**same oils or fats without vitamin A**	**Relative (95% CI)**	**Absolute (95% CI)**		
**Type-2 diabetes mellitus**												
1[5]	observational studies	extremely serious[i]	not serious	not serious	not serious	none	1273/101178 (1.3%)	1322/92625 (1.4%)	**RR 0.88** (0.82 to 0.95)	**2 fewer per 1 000** (from 3 fewer to 1 fewer)	⨁◯◯◯ Very low	

CI: confidence interval; **MD:** mean difference; **RR:** risk ratio

Explanations

a. Downgraded by one level for RoB since 1 out of 2 included studies was rated with a high RoB, and none of the included studies was rated with a low RoB

b. Downgraded by one level for RoB since the included study was rated with some concerns for RoB

c. Downgraded by one level for imprecision. There was only one study included, but the total sample size was larger than 400 (n=412)

d. Downgraded by two levels for imprecision since results are derived from one study, where total sample size was very low (n<100).

e. This is a single study so inconsistency cannot be judged.

f. Downgraded by one level for imprecision. There was only one study included, but the total sample size was larger than 400 (n=413)

g. Downgraded by one level for imprecision since results are derived from two small studies with a low total sample size (n=110).

h. Downgraded by two levels for RoB since the included study was rated with high RoB

i. Downgraded by three levels for RoB since the study included was a birth cohort study rated with serious concerns for RoB with the ROBINS-I tool

References

1.Candelaria, L. V., Magsadia, C. R., Velasco, R. E., Pedro, M. R., Barba, C. V., Tanchoco, C. C.. The effect of vitamin A-fortified coconut cooking oil on the serum retinol concentration of Filipino children 4-7 years old. Asia Pacific journal of clinical nutrition; 2005.

2.Atalhi, N., El Hamdouchi, A., Barkat, A., Elkari, K., Hamrani, A., El Mzibri, M., Haskell, M. J., Mokhtar, N., Aguenaou, H.. Combined consumption of a single high-dose vitamin A supplement with provision of vitamin A fortified oil to households maintains adequate milk retinol concentrations for 6 months in lactating Moroccan women. Physiologie appliquee, nutrition et metabolisme [Applied physiology, nutrition, and metabolism]; 2020.

3.Marliyati, S. A., Martianto, D., Andarwulan, N., Fauzi, S.. Efficacy of non-branded cooking oil fortified with carotene from RPO on blood retinol and IgG of children aged 7-9 years. Pakistan journal of nutrition; 2016.

4.Donglan, Wang, Qingmin, Xiao, Yan, Hong, Shutian, L.,I., Weiqiang, Chen, Yingyong, Cheng. Effects of vitamin A-fortified edible oil on improving the immune function of children. Acta Nutrimenta Sinica; 2006.

5.Keller, A., Angquist, L., Jacobsen, R., Vaag, A., Heitmann, B. L.. A retrospective analysis of a societal experiment among the Danish population suggests that exposure to extra doses of vitamin A during fetal development may lower type 2 diabetes mellitus (T2DM) risk later in life. British journal of nutrition; 2017.

Table 3. GRADE Assessment – Vitamin D fortification vs. no fortification with vitamin D – Secondary outcomes

Certainty assessment							№ of patients		Effect		Certainty	Importance
**№ of studies**	**Study design**	**Risk of bias**	**Inconsistency**	**Indirectness**	**Imprecision**	**Other considerations**	**vitamin D fortified oils or fats**	**same oils or fats without vitamin D**	**Relative (95% CI)**	**Absolute (95% CI)**		
**Intake of dietary vitamin D (follow-up: 12 weeks; assessed with: mcg/day)**												
1[1]	randomised trials	not serious	not serious[a]	not serious	very serious[b]	none	30	32	-	MD **22.35 mcg lower** (52.88 lower to 8.18 higher)	⊕⊕◯◯ Low	IMPORTANT
**Serum parathyreoid hormone (PTH) (follow-up: 12 weeks; assessed with: pmol/L)**												
1[1]	randomised trials	not serious	not serious[a]	not serious	very serious[b]	none	18	18	-	MD **0.1 pmol/L higher** (0.99 lower to 1.99 higher)	⊕⊕◯◯ Low	IMPORTANT
**Bone alkaline phosphatase (B-ALP) (follow-up: 12 weeks; assessed with: IU/L)**												

Certainty assessment							№ of patients		Effect		Certainty	Importance
№ of studies	Study design	Risk of bias	Inconsistency	Indirectness	Imprecision	Other considerations	vitamin D fortified oils or fats	same oils or fats without vitamin D	Relative (95% CI)	Absolute (95% CI)		
1[1]	randomised trials	not serious	not serious[a]	not serious	very serious[b]	none	18	18	-	MD **5.76 IU/L higher** (0.12 lower to 11.64 higher)	⊕⊕◯◯ Low	IMPORTANT
**Incidence of gestational diabetes mellitus**												
1[2]	observational studies	very serious[c]	not serious[a]	not serious	serious[d]	none	297/14016 (2.1%)	361/14855 (2.4%)	**RR 0.87** (0.75 to 1.01)	**3 fewer per 1 000** (from 6 fewer to 0 fewer)	⊕◯◯◯ Very low	IMPORTANT
**Weight gain during pregnancy - not measured**												
-	-	-	-	-	-	-	-	-	-	-	-	IMPORTANT
**Preeclampsia**												
1[3]	observational studies	very serious[c]	not serious[a]	not serious	serious[d]	none	1310/35124 (3.7%)	1370/38113 (3.6%)	**RR 1.04** (0.96 to 1.12)	**1 more per 1 000** (from 1 fewer to 4 more)	⊕◯◯◯ Very low	IMPORTANT
**Gestational hypertension**												

Certainty assessment							№ of patients		Effect		Certainty	Importance
**№ of studies**	**Study design**	**Risk of bias**	**Inconsistency**	**Indirectness**	**Imprecision**	**Other considerations**	**vitamin D fortified oils or fats**	**same oils or fats without vitamin D**	**Relative (95% CI)**	**Absolute (95% CI)**		
1[3]	observational studies	very serious[c]	not serious[a]	not serious	serious[d]	none	399/35124 (1.1%)	396/38113 (1.0%)	**RR 1.09** (0.95 to 1.26)	**1 more per 1 000** (from 1 fewer to 3 more)	⨁◯◯◯ Very low	
**Eclampsia**												
1[3]	observational studies	very serious[c]	not serious[a]	not serious	serious[d]	none	267/35124 (0.8%)	396/38113 (1.0%)	**RR 0.98** (0.83 to 1.16)	**0 fewer per 1 000** (from 2 fewer to 2 more)	⨁◯◯◯ Very low	
**Preterm birth - not measured**												
-	-	-	-	-	-	-	-	-	-	-	-	IMPORTANT
**Low birth weight (<2500 g)**												
1[4]	observational studies	very serious[c]	not serious[a]	not serious	serious[d]	none	301/4836 (6.2%)	308/5716 (5.4%)	**RR 1.16** (0.99 to 1.35)	**9 more per 1 000** (from 1 fewer to 19 more)	⨁◯◯◯ Very low	IMPORTANT
**Postpartum depression - not measured**												
-	-	-	-	-	-	-	-	-	-	-	-	IMPORTANT
**Postpartum haemorrhage - not measured**												

Certainty assessment							№ of patients		Effect		Certainty	Importance
№ of studies	Study design	Risk of bias	Inconsistency	Indirectness	Imprecision	Other considerations	vitamin D fortified oils or fats	same oils or fats without vitamin D	Relative (95% CI)	Absolute (95% CI)		
-	-	-	-	-	-	-	-	-	-	-	-	IMPORTANT
**Breastmilk vitamin D content of lactating women - not measured**												
-	-	-	-	-	-	-	-	-	-	-	-	IMPORTANT
**BMI**												
1[5]	observational studies	very serious[c]	not serious[a]	not serious	not serious	none	5029	5803	-	MD **0.1 kg/m² lower** (0.17 lower to 0.03 lower)	⊕⊕◯◯ Low	IMPORTANT
**Overweight**												
1[5]	observational studies	very serious[c]	not serious[a]	not serious	not serious	none	1172/5029 (23.3%)	1469/5803 (25.3%)	**RR 0.92** (0.86 to 0.98)	**20 fewer per 1 000** (from 35 fewer to 5 fewer)	⊕⊕◯◯ Low	IMPORTANT
**Obesity**												
1[5]	observational studies	very serious[c]	not serious[a]	not serious	not serious	none	523/5029 (10.4%)	708/5803 (12.2%)	**RR 0.85** (0.77 to 0.95)	**18 fewer per 1 000** (from 28 fewer to 6 fewer)	⊕⊕◯◯ Low	IMPORTANT

Certainty assessment							№ of patients		Effect		Certainty	Importance
№ of studies	Study design	Risk of bias	Inconsistency	Indirectness	Imprecision	Other considerations	vitamin D fortified oils or fats	same oils or fats without vitamin D	Relative (95% CI)	Absolute (95% CI)		
**Type-1 diabetes mellitus**												
1[6]	observational studies	very serious[c]	not serious[a]	not serious	not serious	none	298/127207 (0.2%)	404/134749 (0.3%)	**RR 0.78** (0.67 to 0.91)	**1 fewer per 1 000** (from 1 fewer to 0 fewer)	⊕⊕◯◯ Low	IMPORTANT
**Type-2 diabetes mellitus - not measured**												
-	-	-	-	-	-	-	-	-	-	-	-	IMPORTANT
**Fracture events**												
1[7]	observational studies	very serious[c]	not serious[a]	not serious	not serious	none	12330/104406 (11.8%)	16058/113577 (14.1%)	**RR 0.84** (0.82 to 0.85)	**23 fewer per 1 000** (from 25 fewer to 21 fewer)	⊕⊕◯◯ Low	IMPORTANT
**Childhood asthma**												
1[8]	observational studies	not serious	not serious[a]	not serious	not serious	none	1427/106347 (1.3%)	1613/115900 (1.4%)	**RR 0.96** (0.90 to 1.03)	**1 fewer per 1 000** (from 1 fewer to 0 fewer)	⊕⊕◯◯ Low	IMPORTANT
**Hypertension - not measured**												
-	-	-	-	-	-	-	-	-	-	-	-	IMPORTANT

Certainty assessment							№ of patients		Effect		Certainty	Importance
**№ of studies**	**Study design**	**Risk of bias**	**Inconsistency**	**Indirectness**	**Imprecision**	**Other considerations**	**vitamin D fortified oils or fats**	**same oils or fats without vitamin D**	**Relative (95% CI)**	**Absolute (95% CI)**		
**Cardiovascular disease - not measured**												
-	-	-	-	-	-	-	-	-	-	-	-	IMPORTANT
**Respiratory infections - not measured**												
-	-	-	-	-	-	-	-	-	-	-	-	IMPORTANT
**Menopause onset - not measured**												
-	-	-	-	-	-	-	-	-	-	-	-	IMPORTANT
**IBD**												
1[9]	observational studies	very serious[c]	not serious[a]	not serious	not serious	none	875/98856 (0.9%)	1102/108044 (1.0%)	**RR 0.87** (0.79 to 0.95)	**1 fewer per 1 000** (from 2 fewer to 1 fewer)	⨁⨁◯◯ Low	
**Total cholesterol (follow-up: 12 weeks; assessed with: mmol/L)**												
1[10]	randomised trials	not serious	not serious[a]	not serious	very serious[b]	none	30	32	-	MD **0.01 mmol/L higher** (0.38 lower to 0.4 higher)	⨁⨁◯◯ Low	

Certainty assessment							№ of patients		Effect		Certainty	Importance
№ of studies	Study design	Risk of bias	Inconsistency	Indirectness	Imprecision	Other considerations	vitamin D fortified oils or fats	same oils or fats without vitamin D	Relative (95% CI)	Absolute (95% CI)		
**LDL Cholesterol (follow-up: 12 weeks; assessed with: mmol/L)**												
1[10]	randomised trials	not serious	not serious[a]	not serious	very serious[b]	none	30	32	-	MD **0.02 mmol/L higher** (0.25 lower to 0.29 higher)	⊕⊕◯◯ Low	
**HDL cholesterol (follow-up: 12 weeks; assessed with: mmol/L)**												
1[10]	randomised trials	not serious	not serious[a]	not serious	very serious[b]	none	30	32	-	MD **0.03 mmol/L higher** (0.07 lower to 0.13 higher)	⊕⊕◯◯ Low	
**Triglyceride (follow-up: 12 weeks; assessed with: mmol/L)**												
1[10]	randomised trials	not serious	not serious[a]	not serious	very serious[b]	none	30	32	-	MD **0.02 mmol/L higher** (0.34 lower to 0.38 higher)	⊕⊕◯◯ Low	
**Energy intake (kcal) (follow-up: 12 weeks)**												

Certainty assessment							№ of patients		Effect		Certainty	Importance
**№ of studies**	**Study design**	**Risk of bias**	**Inconsistency**	**Indirectness**	**Imprecision**	**Other considerations**	**vitamin D fortified oils or fats**	**same oils or fats without vitamin D**	**Relative (95% CI)**	**Absolute (95% CI)**		
1[1]	randomised trials	not serious	not serious[a]	not serious	very serious[b]	none	31	30	-	MD **280 kcal lower** (513.33 lower to 46.67 lower)	⨁⨁◯◯ Low	
**High birth weight (>4000 g)**												
1[4]	observational studies	very serious[c]	not serious[a]	not serious	not serious	none	407/4836 (8.4%)	583/5716 (10.2%)	**RR 0.83** (0.73 to 0.93)	**17 fewer per 1 000** (from 28 fewer to 7 fewer)	⨁⨁◯◯ Low	
**Coeliac disease**												
1[11]	observational studies	very serious[c]	not serious[a]	not serious	serious[d]	none	148/98856 (0.1%)	199/108044 (0.2%)	**RR 0.81** (0.66 to 1.01)	**0 fewer per 1 000** (from 1 fewer to 0 fewer)	⨁◯◯◯ Very low	

CI: confidence interval; **MD:** mean difference; **RR:** risk ratio

Explanations

a. This is a single study so inconsistency cannot be judged.

b. Downgraded by two levels for imprecision since results are derived from one study, where total sample size was very low (n<100).

c. Downgraded by two levels for RoB since the study included was a birth cohort study rated with some concerns for RoB with the ROBINS-I tool

d. Downgraded by one level for imprecision since results are derived from one study, and the confidence interval was crossing the line of no effect

References

1.Ghasemifard, N., Hassanzadeh-Rostami, Z., Abbasi, A., Naghavi, A. M., Faghih, S.. Effects of vitamin D-fortified oil intake versus vitamin D supplementation on vitamin D status and bone turnover factors: a double blind randomized clinical trial. Clinical nutrition ESPEN; 2022.

2.Keller, A., Stougard, M., Frederiksen, P., Thorsteinsdottir, F., Vaag, A., Damm, P., Jacobsen, R., B, L.,Heitmann. In utero exposure to extra vitamin D from food fortification and the risk of subsequent development of gestational diabetes: the D-tect study. Nutrition journal; 2018.

3.Stougaard, M., Damm, P., Frederiksen, P., Jacobsen, R., Heitmann, B. L.. Extra vitamin D from fortification and the risk of preeclampsia: The D-tect Study. PLoS ONE [Electronic Resource]; 2018.

4.Jensen, C. B., Berentzen, T. L., Gamborg, M., Sorensen, T. I., Heitmann, B. L.. Does prenatal exposure to vitamin D-fortified margarine and milk alter birth weight? A societal experiment. British journal of nutrition; 2014.

5.Jensen, C. B., Gamborg, M., Berentzen, T. L., Sørensen, T. I. A., Heitmann, B. L.. Prenatal exposure to vitamin-D from fortified margarine and milk and body size at age 7 years. European journal of clinical nutrition; 2015.

6.Jacobsen, R., Hypponen, E., Sorensen, T. I., Vaag, A. A., Heitmann, B. L.. Gestational and Early Infancy Exposure to Margarine Fortified with Vitamin D through a National Danish Programme and the Risk of Type 1 Diabetes: The D-Tect Study. PLoS ONE [Electronic Resource]; 2015.

7.Handel, M. N., Frederiksen, P., Osmond, C., Cooper, C., Abrahamsen, B., Heitmann, B. L.. Prenatal exposure to vitamin D from fortified margarine and risk of fractures in late childhood: period and cohort results from 222 000 subjects in the D-tect observational study. British journal of nutrition; 2017.

8.Thorsteinsdottir, F., Maslova, E., Jacobsen, R., Frederiksen, P., Keller, A., Backer, V., Heitmann, B. L.. Exposure to Vitamin D Fortification Policy in Prenatal Life and the Risk of Childhood Asthma: Results From the D-Tect Study. Nutrients; 2019.

9.Duus, K. S., Moos, C., Frederiksen, P., Andersen, V., Heitmann, B. L.. Prenatal and Early Life Exposure to the Danish Mandatory Vitamin D Fortification Policy Might Prevent Inflammatory Bowel Disease Later in Life: A Societal Experiment. Nutrients; 2021.

10.Ghasemifard, N., Nasimi, N., Hassanzadeh-Rostami, Z., Abbasi, A., Faghih, S.. Effects of vitamin D fortified canola oil on vitamin D and lipid profiles in healthy adults: A double-blind randomized trial. Iranian Journal of Nutrition Sciences and Food Technology; 2020.

11.Moos, C., Duus, K. S., Frederiksen, P., Heitmann, B. L., Andersen, V.. Exposure to the Danish Mandatory Vitamin D Fortification Policy in Prenatal Life and the Risk of Developing Coeliac Disease-The Importance of Season: A Semi Ecological Study. Nutrients; 2020.
